# Crafting the Organic–Inorganic Interface with a Bridging Architecture for Solid‐State Li‐O_2_ Batteries

**DOI:** 10.1002/advs.202503664

**Published:** 2025-06-19

**Authors:** Minghui Li, Kecheng Pan, Dulin Huang, Jing Wu, Zhenzhen Li, Yaying Dou, Zhang Zhang, Zhen Zhou

**Affiliations:** ^1^ Interdisciplinary Research Center for Sustainable Energy Science and Engineering (IRC4SE2) School of Chemical Engineering Zhengzhou University Zhengzhou 450001 China

**Keywords:** bridging architecture, composite solid electrolytes, Li‐O_2_ batteries, organic–inorganic interface

## Abstract

Solid‐state lithium‐oxygen batteries (SSLOBs) are offering unparalleled safety and exceptional electrochemical performance. Despite their promise, composite solid electrolytes (CSEs) fabricated through mechanical hybridization consistently manifest pronounced ceramic particle aggregation. In this study, a thin and flexible CSE is developed by integrating Li_10_GeP_2_S_12_ (LGPS) with poly(vinylidene fluoride‐co‐hexafluoropropylene) (PVDF‐HFP) and implementing silane coupling agents to form a bridging framework across the organic–inorganic heterojunction interfaces. The engineered CSE exhibited remarkable room‐temperature ionic conductivity reaching 1.05 × 10^−4^ S cm^−1^, superior electrochemical stability within an expanded voltage window extending to 4.9 V versus Li/Li^+^. Furthermore, lithium symmetrical cells revealed uniform lithium deposition/dissolution behavior over 3000 h. Integration of the thin‐film CSE into SSLOBs yielded devices achieving specific discharge capacities of 12874 mAh g^−1^, coupled with superior long‐term operational stability throughout 120 cycles. The enhanced interfacial adhesion forces observed between the heterogeneous phases play a pivotal role in maintaining space charge region stability, subsequently promoting accelerated lithium‐ion diffusion kinetics while optimizing charge transfer processes at the electrochemical interfaces. The systematic study presents an innovative synthetic strategy for engineering dimensionally‐confined, sulfide‐enriched CSEs.

## Graphical abstract

Through the strategic incorporation of functionalized silane coupling agents within the electrolytic matrix, a pioneering methodology is demonstrated that facilitates the formation of molecular bridges across the organic/inorganic heterogeneous interface. In situ coupling reactions transform the traditional weak physical contact between organic/inorganic composite components into stronger chemical interactions, effectively addressing the interfacial barrier issue between the inorganic and organic phases.

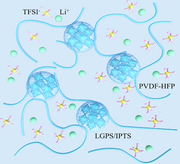

## 1 Introduction

Lithium‐oxygen batteries (LOBs), which offer an impressive theoretical specific energy of up to 3623 Wh/kg,^[^ ^1^ ^]^ encounter substantial operational limitations, predominantly manifested through electrochemical instability and inherent volatility of organic electrolytes.^[^ ^2^ ^]^ The incorporation of solid‐state electrolytes (SSEs) represents a transformative approach in circumventing these fundamental limitations, offering both unprecedented energy density and superior safety.^[^ ^3^, ^4^, ^5^ ^]^ A single solid electrolyte falls short of the necessary requirements for advanced applications. However, the strategic combination of inorganic and polymeric elements within composite solid electrolytes (CSEs) yields a remarkable synergy that enhances their performance to unprecedented levels. This amalgamation not only addresses the individual limitations of each component but also harnesses their collective strengths to achieve superior electrochemical performances.

Among various categories of SSEs, sulfide‐based architectures distinguish themselves through their extraordinary ionic conductivity profiles, exhibiting values spanning 10^−3^ to 10^−2^ S cm^−1^ at ambient temperature—a performance metric that parallels of traditional organic liquid electrolyte matrices.^[^ ^6^ ^]^ Nevertheless, the practical implementation of these materials remains constrained by their inherent mechanical fragility and the dimensional limitations of the resultant compressed specimens, whereby the propagation of microscopic fissures can precipitate catastrophic electronic short‐circuits within solid‐state electrochemical devices.^[^ ^7^ ^]^ Therefore, substantial investigative endeavors have been directed toward the engineering of dimensionally minimized, mechanically compliant sulfide‐based SSEs through the strategic incorporation of polymeric electrolyte matrices, thereby simultaneously addressing both conformational adaptability and enhanced structural integrity. Distinct from conventional inorganic SSEs, polymer electrolytes manifest substantially lower charge carrier mobility and compromised Li^+^ transport efficiency. Poly(vinylidene fluoride‐co‐hexafluoropropylene) (PVDF‐HFP), notable for its high dielectric constant (ε = 8.4) and excellent resistance to oxidation, emerges as a preeminent candidate for polymer electrolyte matrices in high‐voltage solid‐state batteries.^[^ ^8^ ^]^ The non‐crystalline segment of PVDF‐HFP (‐HFP) orchestrate expedited Li^+^ mobility, while the crystalline phase (‐VDF) domains simultaneously impart requisite mechanical fortification. Nevertheless, solvent‐deficient PVDF‐HFP‐derived membranous architectures unveil insufficient ionic conductivity and diminished Li^+^ transference number, attributed predominantly to restricted cationic mobility within the polymer matrix.

In response to these fundamental constraints, the deliberate integration of inorganic nanoscale additives within PVDF‐HFP‐derived electrolytic matrices represents a compelling approach to effectively augmenting the electrochemical stability, mechanical robustness, and overall safety of the electrolyte. For instance, Song et al. developed a quasi‐solid‐state CSE integrating PVDF‐HFP with lithium aluminum germanium phosphate (LAGP), supported by a liquid electrolyte, achieving a cycle life of 152 cycles for LOBs under a capacity cutoff of 1000 mAh g^−1^ at 0.1 mA cm^−2^.^[^ ^9^ ^]^ Zhang et al. fabricated a free‐standing composite Li^+^ conducting electrolyte with PVDF‐HFP and Li_1+x_Al_x_Ti_2‐x_(PO_4_)_3_ (LATP) via tape‐casting, showcasing promising air‐breathing and cycling capability in ambient air for hybrid Li‐air batteries.^[^ ^10^ ^]^ Le et al. innovated a composite gel electrolyte comprising PVDF‐HFP and aluminum‐doped Li_0.33_La_0.56_TiO_3_ (A‐LLTO), enveloped in a modified SiO_2_ layer, demonstrating a prolonged cycle life of 72 cycles for LOBs operating under a limited capacity mode of 1000 mAh g^−1^.^[^ ^11^ ^]^

Notwithstanding these significant advances, contemporary research initiatives have predominantly centered on optimizing the mechanical resilience and electrochemical cycling efficacy of LOBs, whereas the fundamental challenges concerning the organic‐inorganic interfacial compatibility within CSEs remain insufficiently investigated. Within CSEs, the heterogeneous interface region, where molecular‐scale interactions between the dispersed inorganic constituents and the continuous polymeric framework occur, fundamentally governs the electrochemical characteristics and functional parameters of the energy storage device. Optimal spatial distribution coupled with favorable interfacial compatibility between the inorganic additives and the polymeric matrix engenders the formation of contiguous ion‐conducting networks, which prove instrumental in facilitating stable Li^+^ migration while simultaneously suppressing the nucleation and propagation of lithium dendrites.

Our experimental approach encompasses the rational design and synthesis of an advanced composite electrolyte system through the deliberate integration of 3‐isopropoxytriethylsilane (IPTS) as an interfacial modifier within the poly(vinylidene fluoride‐co‐hexafluoropropylene)/Li_10_GeP_2_S_12_ (PVDF‐HFP/LGPS) framework, resulting in markedly enhanced chemical resilience of the LGPS component and optimized interfacial compatibility at the organic‐inorganic interface. The multifunctional organic silane coupling agent (IPTS) establishes robust covalent linkages with both the polymeric framework and inorganic moieties, orchestrating an interfacial bridging architecture that facilitates the molecular integration of the PVDF‐HFP matrix with the Li_10_GeP_2_S_12_ phase. The innovative molecular modification boosts interfacial compatibility and adhesion mechanisms at the organic‐inorganic boundaries, culminating in optimized Li^+^ pathways and elevated electrochemical performances of the SSE system. The as‐prepared PILS exhibits superior Li^+^ conductivity of 1.05 × 10^−4^ S cm^−1^ at ambient temperature. Consequently, SSLOBs incorporating this thin PILS membrane demonstrate exceptional cycling endurance (120 cycles) and a remarkable specific capacity (12 874 mAh g^−1^). Notably, the assembled Li|PILS|LiFePO_4_ cells achieve stable cycling over 968 cycles at 0.5 C, with an impressive initial discharge capacity of 150.3 mAh g^−1^ at room temperature and a capacity retention rate of 93%. This investigation elucidates an innovative interfacial engineering strategy that optimizes polymer‐ceramic heterojunction characteristics within CSEs through strategic in situ molecular coupling protocols, establishing a paradigm for the fabrication of ultrathin, high‐performance sulfide‐based electrolyte architectures and broadening the technological horizons of LOBs.

## 2 Results and Discussion

### 2.1 Characterization of PILS Electrolytes

**Figure 1:**
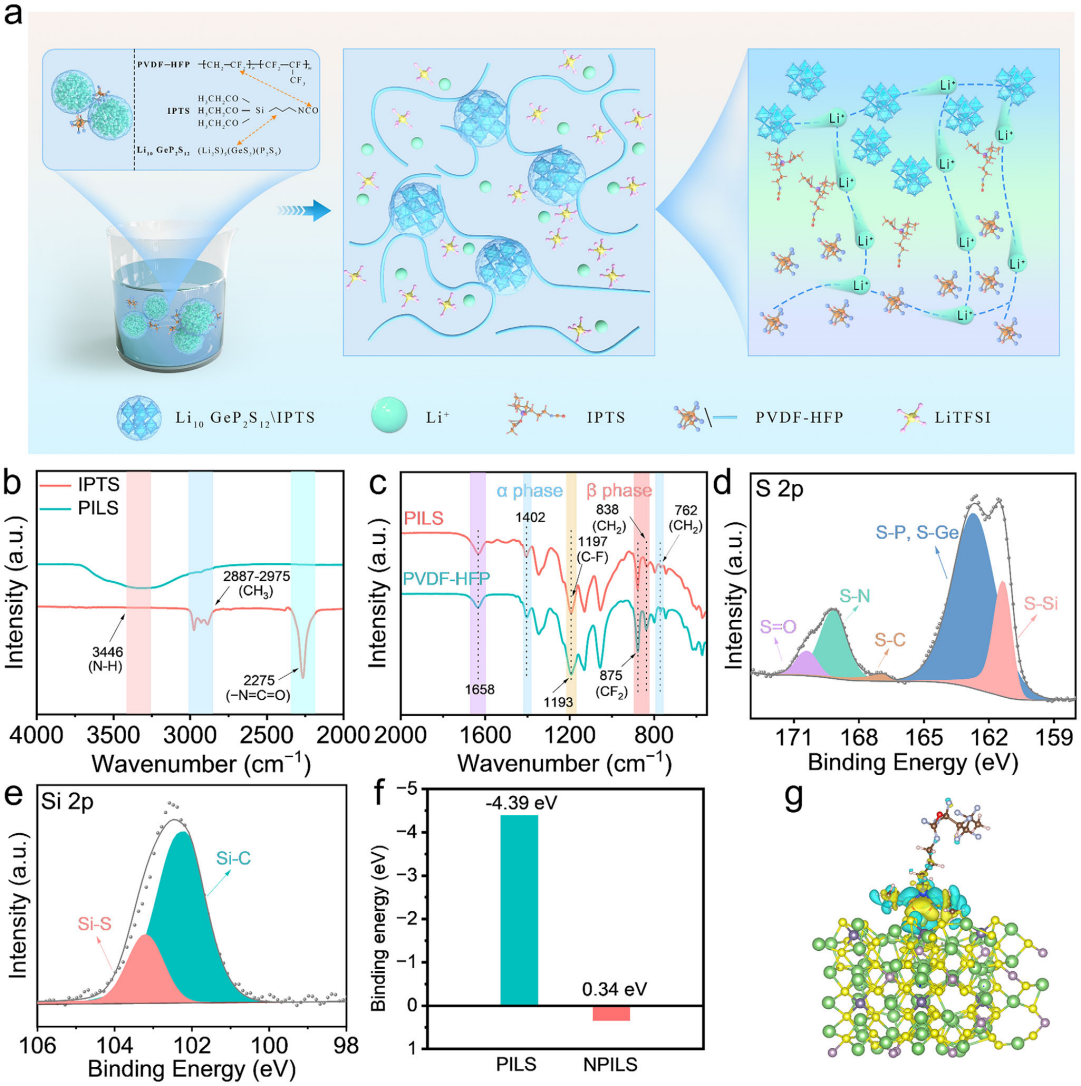
a) Schematic diagram of the chemical reaction between IPTS, LGPS and PVDF‐HFP. FTIR spectra of PILS, IPTS and PVDF‐HFP for b) 2000–4000 cm^−1^ and c) 600–2000 cm^−1^. XPS for d) S 2p and e) Si 2p of PILS. f) The adsorption energy of PILS and NPILS (PVDF‐HFP/LGPS without IPTS). g) Charge density difference calculation of PILS structure.

In light of the characteristic affinity between isocyanate functionalities and lewis acidic polymer backbone structures, IPTS was identified as an optimal coupling agent for the modification of PVDF‐HFP chains through selective nucleophilic addition protocols, with the mechanistic pathway illustrated schematically in **Figure** **1**a. The approach demonstrates remarkable efficacy in both enhancing and preserving Li^+^ transport kinetics through the LGPS framework, concurrent with a notable reduction in crystallite agglomeration dynamics. Simultaneously, ceramic and polymer materials are linked via robust chemical bonds within the PILS, creating a pathway for swift Li^+^ transport.^[^ ^12^ ^]^ Consequently, the electrolyte potentially harbors three distinct Li^+^ transport conduits, contributing to superior ionic conductivity, reduced energetic barriers for ion transport, and optimized Li^+^ transference numbers.

In the Fourier transform infrared (FTIR) spectrum of IPTS (Figure 1b), two discernible peaks are attributed to the isocyanate and ethyl functional groups. Specifically, the peak at 2275 cm^−1^ indicates the isocyanate group, while the bands from 2887 to 2975 cm^−1^ correspond to the ethyl groups.^[^ ^13^ ^]^ However, the original vibrational signatures were supplanted by emergent absorption bands centered at 3446 cm^−1^, characteristic of N‐H stretching vibrations,^[^ ^14^ ^]^ thus substantiating the mechanistic involvement of these functional groups in molecular interactions with the electrolyte species, ultimately facilitating the genesis of PILS. Furthermore, the characteristic absorbance bands observed at 762 and 1402 cm^−1^ signify the presence of the α‐phase, while those at 875 and 838 cm^−1^ are ascribed to the symmetric stretching vibrations of the CF_2_ and CH_2_ group,^[^ ^15^ ^]^ as depicted in Figure 1c. Furthermore, the emergence of a peak at 1658 cm^−1^ suggests an interaction between the C‐H group of PVDF‐HFP and the CF_3_ group of TFSI^−^.^[^ ^16^ ^]^

To substantiate the intimate chemical integration between LGPS and PVDF‐HFP, the prepared PILS was analyzed through X‐ray photoelectron spectroscopy (XPS), with the findings presented in Figure 1d,e. The XPS for the C 1s (Figure S1a, Supporting Information) exhibits peaks at 284.8, 286.8, 290.8, and 292.5 eV, corresponding to the C‐C, C‐O, C = O, and C─F bonds, respectively.^[^ ^17^ ^]^ In the S 2p spectrum (Figure 1d; Figure S1b, Supporting Information), the peaks at 161.5, 163, 167, 169, and 170.5 eV are attributed to the S‐Si, S‐P/S‐Ge, S‐C, S‐N, and S = O groups.^[^ ^18^ ^]^ The presence of S‐Si bonds, unequivocally confirms the heterogeneous chemical bonding between IPTS and the inorganic LGPS particulate matrix. Characteristic peaks corresponding to S‐P/S‐Ge indicate the efficacious integration of LGPS superionic conductor matrix. Meanwhile, peaks of S‐C, S‐N, and S = O are assigned to lithium bis(trifluoromethanesulfonyl)imide (LiTFSI).^[^ ^19^ ^]^ Deconvolution of the high‐resolution Si 2p photoelectron spectrum reveals two predominant spectral components: a characteristic emission at 102.1 eV, attributable to Si‐C covalent linkages within the organosilane moiety of IPTS, and a higher‐energy feature centered at 103.2 eV, diagnostic of Si─S interfacial bonds (Figure 1e; Figure S1c, Supporting Information).^[^ ^20^ ^]^ This spectroscopic evidence further corroborates the establishment of robust chemical connectivity between the functionalized silane coupling agent and the sulfide‐based solid electrolyte matrix. The complementary spectroscopic evidence derived from FTIR and XPS convergently demonstrates that the IPTS serves as an efficacious molecular bridge, facilitating robust interfacial integration between the inorganic Li_10_GeP_2_S_12_ superionic conductor and the fluoropolymer (PVDF‐HFP) matrix architecture. The integration of IPTS not only mitigates the aggregation of LGPS but also enhances ionic conductivity by establishing a seamless route for Li^+^ transport. The multifunctional silane coupling agent serves as an architecturally sophisticated molecular scaffold within the electrolyte matrix, facilitating expeditious ion transport through precisely engineered channels while simultaneously enhancing the comprehensive electrochemical performance of these CSEs.

Density Functional Theory (DFT) calculations were performed to elucidate the mechanistic underpinnings of IPTS‐mediated interfacial phenomena. Possible intricate configurations in the PILS based on simulations are shown in Figure S2 (Supporting Information). In Figure 1f, the interaction energy for the LGPS/PVDF‐HFP system without IPTS was determined to be 0.34 eV, indicating a weak interaction that hampers Li^+^ transport through the biphasic interface. In contrast, the adsorption energy for the PILS architecture was significantly reduced to ‐4.39 eV. These computational findings highlight a notable increase in the affinity between the ceramic and polymer components facilitated by the IPTS, greatly strengthening interfacial adhesion and mechanical integrity. Furthermore, the inherent electronic heterogeneity between the constituent phases manifests as a pronounced band edge discontinuity, consequently inducing the formation of a space charge region at the heterojunction interface. Through quantum mechanical calculations of charge density difference (CDD) distributions in both pristine and IPTS‐functionalized PVDF‐HFP/LGPS heterosystems, we elucidate the fundamental mechanisms governing interfacial electronic interactions. The spatial distribution of electronic charge redistribution, illustrated in Figure 1g and Figure S3 (Supporting Information), reveals regions of electron accumulation (depicted in yellow) and electron depletion (rendered in blue), demarcating the charge transfer topology.^[^ ^21^ ^]^ Relative to its unfunctionalized counterpart, the silane‐modified heterosystem demonstrates markedly enhanced charge density perturbations at the PVDF‐HFP/LGPS interface, suggesting amplified charge transfer processes and strengthened interphase interactions. The interfacial space charge region facilitates accelerated Li^+^ transport dynamics and coordinates enhanced charge‐transfer kinetics across the heterojunction.^[^ ^22^ ^]^ Additionally, the locally conjugated structure on the polymer side also aids in Li^+^ transfer within the space charge layer. To further substantiate the advantageous effects of IPTS on ionic conductivity enhancement within the system, we quantified the energetic barriers impeding Li^+^ diffusion across the PVDF‐HFP/LGPS interface, conducting comparative analyses under conditions both incorporating and devoid of IPTS functionalization. Our calculations reveal a significant reduction in the diffusion barrier when IPTS is present, elucidating the mechanistic role of this silane coupling agent in facilitating expedited Li^+^ transport across the heterogeneous interface (Figure S4, Supporting Information). This reduction in energy barriers highlights the favorable conditions for ion migration provided by the IPTS‐functionalized interface, which is crucial for the system's performance in battery applications. Consequently, DFT calculations unequivocally demonstrate that bifunctional silane coupling agents serve as molecular scaffolds, establishing robust covalent interconnections between inorganic and polymeric constituents, thereby orchestrating the uniform spatial distribution of inorganic moieties throughout the polymeric framework while concomitantly attenuating LGPS agglomeration phenomena. The extraordinary interfacial compatibility engenders unprecedented Li^+^ transport kinetics across the phase boundaries, thereby revealing in substantially elevated ionic conductivity throughout the PILS hierarchical framework.

**Figure 2:**
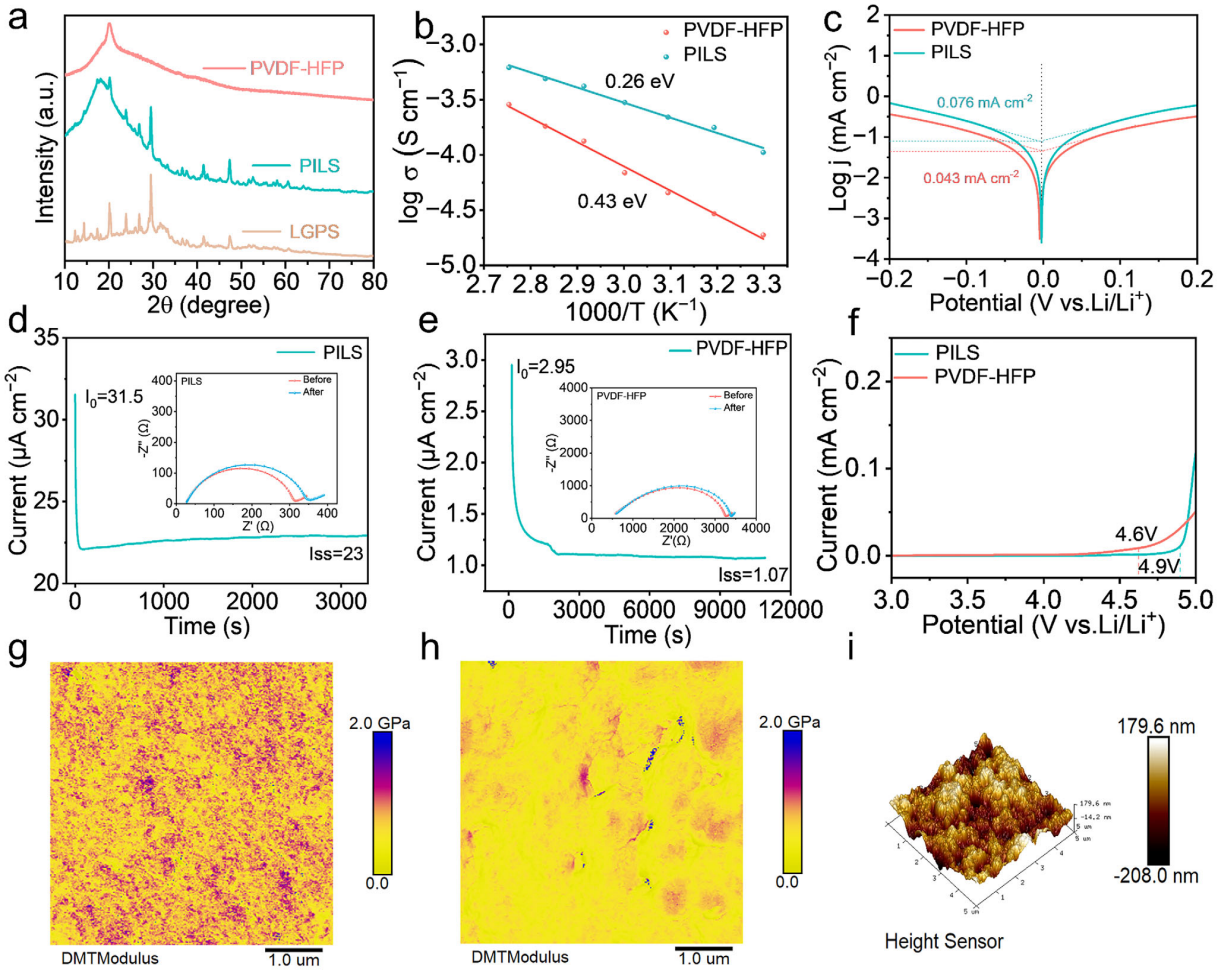
a) XRD patterns. b) Arrhenius plot from 30 to 90 °C. c) Tafel plots of symmetrical cells to obtain the exchange current densities. The chronoamperometry test for d) PILS and e) PVDF‐HFP. The inset corresponds to AC impedance spectra before and after DC polarization. f) LSV curves of PILS and PVDF‐HFP. Young's modulus of g) PILS and h) PVDF‐HFP. i) The AFM height images of PILS.

Additionally, from the X‐ray diffraction (XRD) patterns (**Figure** **2**a) of LGPS, PVDF‐HFP, and PILS, the diffraction peaks at 18.2° and 20.2°, attributed to PVDF‐HFP, are clearly discernible. Notably, LGPS is highly susceptible to moisture and prone to decomposition in most solvents, which significantly reduces its ionic conductivity.^[^ ^23^ ^]^ Structural analysis reveals that, all characteristic diffraction peaks of LGPS observed in PILS can be unambiguously indexed to a pure cubic‐phase crystalline architecture, demonstrating complete concordance with the XRD patterns of pristine LGPS specimens. The preservation of LGPS's crystallographic architecture following the infiltration protocol provides compelling evidence that the incorporation of silane coupling agents effectively attenuates the material's inherent vulnerability to atmospheric and solvent‐induced degradation (Figure S5, Supporting Information). Additionally, although the characteristic diffraction signatures of PVDF‐HFP persist unaltered, a discernible attenuation in peak intensity is manifested, suggesting that the incorporation of inorganic constituents diminishes the crystallinity of the polymeric matrix. XRD analyses performed on CSEs devoid of silane coupling agents (Figure S6, Supporting Information) reveal a marked disappearance of the characteristic LGPS diffraction signatures, thereby substantiating the instrumental role of silane functionalization in preserving the structural integrity of the LGPS phase. Raman spectroscopy confirmed the structural integrity of LGPS within PILS (Figure S7, Supporting Information). Moreover, thermogravimetric analysis (TGA) plots illustrated in Figure S8 (Supporting Information) reveal the remarkable thermal durability of PILS, surpassing that of traditional organic liquid electrolytes known for their flammability. Comprehensive microstructural characterization via scanning electron microscopy (SEM) and transmission electron microscope (TEM) coupled with energy‐dispersive X‐ray spectroscopy (EDS) analyses elucidated the homogeneous spatial distribution of LGPS particles within the PVDF‐HFP polymeric framework (Figures S9 and S10, Supporting Information). Additionally, particle size distribution analysis further assesses the effect of the modification process on particle size (Figure S11, Supporting Information). This unprecedented interfacial compatibility, in conjunction with the meticulously optimized physicochemical integration between the ceramic and polymeric constituents, culminates in the formation of a hierarchically organized 3D network of unimpeded Li^+^ transport channels.

Figure S12 (Supporting Information) elucidates that PILS exhibits significantly lower impedance at room temperature, corresponding to an ionic conductivity of 1.05 × 10^−4^ S cm^−1^. This value is attributed to the inherently high conductivity of LGPS and is superior to that of PVDF‐HFP, which registers at 2.3 × 10^−5^ S cm^−1^. Furthermore, the ionic conductivity of PILS increases to 1.08 × 10^−3^ S cm^−1^ at 90 °C. The activation energies of PILS and PVDF‐HFP were calculated to be 0.26 eV and 0.43 eV through Arrhenius equation (Figure 2b), respectively, confirming a low Li^+^ transport energy barrier and superior ionic conductivity of PILS. To evaluate the kinetics of Li plating and stripping, exchange current densities were computed to gain insights into the electrochemical behavior at the electrode surface. PILS demonstrated an exchange current density of 0.076 mA cm^−2^, which markedly surpasses that of PVDF‐HFP (0.043 mA cm^−2^) (Figure 2c). The notably enhanced exchange current density observed in PILS architectures signifies superior interfacial charge‐transfer kinetics, displaying in accelerated electrochemical reaction rates at the electrode‐electrolyte interface. As high Li^+^ transference number in favor of promoting diffusion and deposition of Li^+^, the Li|PILS|Li and Li|PVDF‐HFP|Li cell was characterized by chronoamperometry measurements. The Li^+^ transference numbers of PILS and PVDF‐HFP were found to be 0.47 and 0.11, respectively (Figure 2d,e). The homogeneous dispersion of LGPS within the PVDF‐HFP matrix and the bridge structure constructed by the silane coupling agent contribute to the enhanced Li^+^ transference number by establishing a swift and uninterrupted pathway for Li^+^ transport. Moreover, PVDF‐HFP can form interactions with Li^+^, effectively restricting anion mobility and encouraging the dissociation of LiTFSI, consistent with Raman spectroscopy, which will be discussed below.

Particularly noteworthy, the PILS system exhibited unprecedented electrochemical stability, maintaining structural integrity without discernible oxidative degradation up to 4.9 V versus Li/Li^+^, thereby substantially surpassing the conventional PVDF‐HFP, which manifests anodic decomposition phenomena at potentials exceeding 4.6 V (Figure 2f). This improvement is attributed to the incorporation of IPTS, which fosters stronger interfacial interactions between LGPS and PVDF‐HFP and the inherent exceptional electrochemical stability of LGPS. The homogeneous distribution of LGPS nanoparticles throughout the polymeric matrix, coupled with enhanced interfacial engineering, synergistically facilitates uniform Li^+^ flux distribution, thereby substantially minimizing deleterious ionic accumulation at the electrode/electrolyte heterojunction. This mitigation helps maintain a consistent and minimal current density, thereby fortifying the stability of PILS under high‐voltage conditions. Atomic force microscopy (AFM) reveals that the Young's modulus of PILS was found to be impressively high at 2 GPa (Figure 2g), exceeding that of PVDF‐HFP, which measured a comparative modulus at 1.5 GPa (Figure 2h). Furthermore, the surface roughness of PILS (Figure 2i) exhibited a lower root‐mean‐square roughness (R_q_) of 57.5 nm compared with PVDF‐HFP (R_q_, 141 nm, Figure S13, Supporting Information). The diminished surface roughness exhibited by PILS indicates enhanced topographical uniformity, thereby facilitating superior electrode‐electrolyte interfacial contact and consequently augmenting the overall electrochemical kinetics and performance.

**Figure 3:**
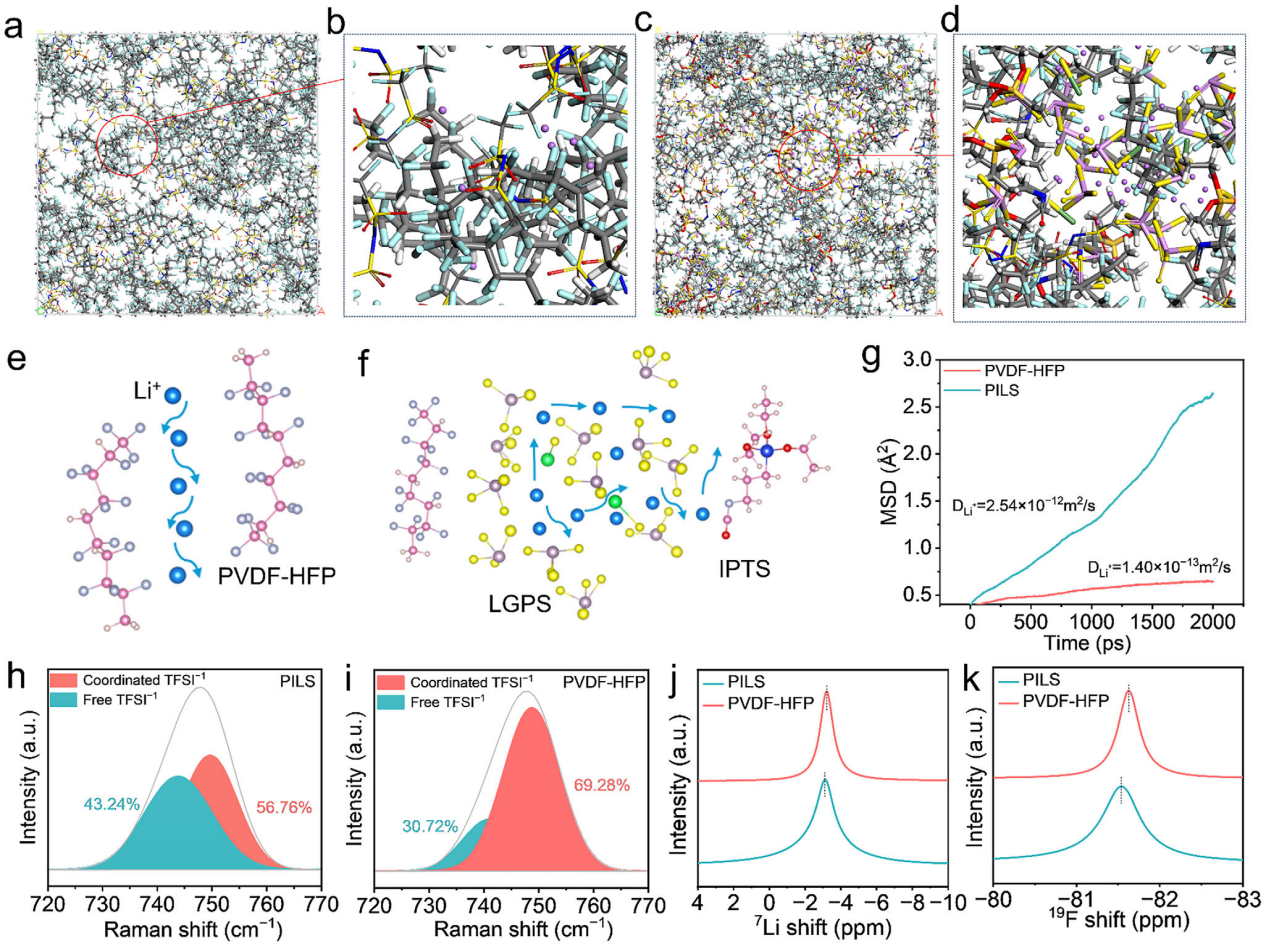
Simulated structures of PVDF‐HFP a,b) and PILS c,d). Schematic diagram of Li^+^ transport for e) PVDF‐HFP and f) PILS. g) MSD versus diffusion time of Li^+^ transport of PVDF‐HFP and PILS. Raman spectra of h) PILS and i) PVDF‐HFP. Solid‐state j) ^7^Li and k) ^19^F SSNMR spectra of PILS and PVDF‐HFP.

Molecular dynamics (MD) simulations were conducted to probe the underlying mechanisms facilitating enhanced ionic mobility (Figure S14, Supporting Information). Representative molecular configurations extracted from the MD trajectories are visualized in **Figure** **3**a,b for the PVDF‐HFP matrix, while analogous structural snapshots of the PILS architecture are depicted in Figure 3c,d. Functionalization via silane coupling agents engenders the development of sophisticated cross‐linking networks within the PILS matrix, culminating in enhanced Li^+^ migration channels at the interface, with the resultant supramolecular organization depicted in Figure 3e,f. Moreover, the introduction of LGPS serves as a critical structure‐directing agent, intensifying the electrostatic and coordination interactions between TFSI^−^ moieties and the PVDF‐HFP polymer framework, thereby yielding markedly improved ionic conductivity. The spatiotemporal dynamics of Li^+^ diffusion was evaluated via mean square displacement (MSD) analysis at ambient temperature (298 K) across both architectures, with the resultant diffusion profiles elucidated in Figure 3g. Quantitative analysis revealed that the Li^+^ diffusion coefficient in the PILS framework reached 2.5 × 10^−12^ m^2^ s^−1^, demonstrating an order of magnitude enhancement compared with the PVDF‐HFP matrix (1.4 × 10^−13^ m^2^ s^−1^). This pronounced disparity in ionic transport kinetics underscores the exceptional charge carrier mobility within the PILS architecture. These theoretical predictions are substantiated by independent experimental measurements, as detailed in the impedance analysis presented in Figure S12 (Supporting Information). Collectively, MD simulations indicate that the C = N bond moiety in the silane coupling agent and the LGPS group displays a critical structural determinant in facilitating enhanced ionic transport phenomena.^[^ ^24^ ^]^ The resultant formation of these precisely engineered interfacial transport networks offers a molecular‐level rationale for the marked enhancement in ionic conductivity observed throughout the PILS framework, illuminating the underlying structure‐function correlation. Raman shifts of the N─S bond in the range of 725–760 cm^−1^ can be divided into dissociated and undissociated LiTFSI, respectively (Figure 3h,i).^[^ ^25^ ^]^ Notably, spectroscopic analyses show that PILS exhibits a significantly higher proportion of dissociated TFSI^−^ anions compared with their coordinated counterparts, with dissociation levels substantially surpassing those observed in PVDF‐HFP matrices in environments lacking LGPS functionalization. This discovery suggests that the incorporation of LGPS extends the dispersion of negative charges within PILS, with the highly electronegative fluoride moieties of PVDF‐HFP serving as conduits for Li^+^ transportation.^[^ ^26^ ^]^ These electrostatically active domains generate localized anion‐exclusion zones, consequently facilitating directional Li^+^ transport through strategically aligned ionic conducting channels, which function as molecular bridges for efficient cation migration. The Li^+^ interfacial dynamics and local ionic environments are studied through solid‐state nuclear magnetic resonance (SSNMR). This analysis revealed a significant downfield shift in the ^7^Li signal for PILS compared with PVDF‐HFP, as shown in Figure 3j. The observed perturbation in electronic density profiles surrounding the Li^+^ coordination sphere indicates weakening of the nitrogen‐mediated dative bonds from TFSI^−^ ligands, consequently promoting the dissolution of LiTFSI clusters through enhanced ionic dissociation pathways.^[^ ^27^ ^]^ Analysis of the ^19^F NMR spectroscopic data reveals a characteristic deshielding effect in the TFSI^−^ resonances (Figure 3k), indicative of hydrogen bonding between the electronegative fluorine sites of TFSI^−^ and the proton‐donating segments of the PVDF‐HFP copolymer matrix.^[^ ^28^ ^]^ The established non‐covalent associations serve as crucial structural determinants in regulating TFSI^−^ mobility, ultimately modulating the charge carrier dynamics and overall ionic transport efficiency across the PILS matrix.

**Figure 4:**
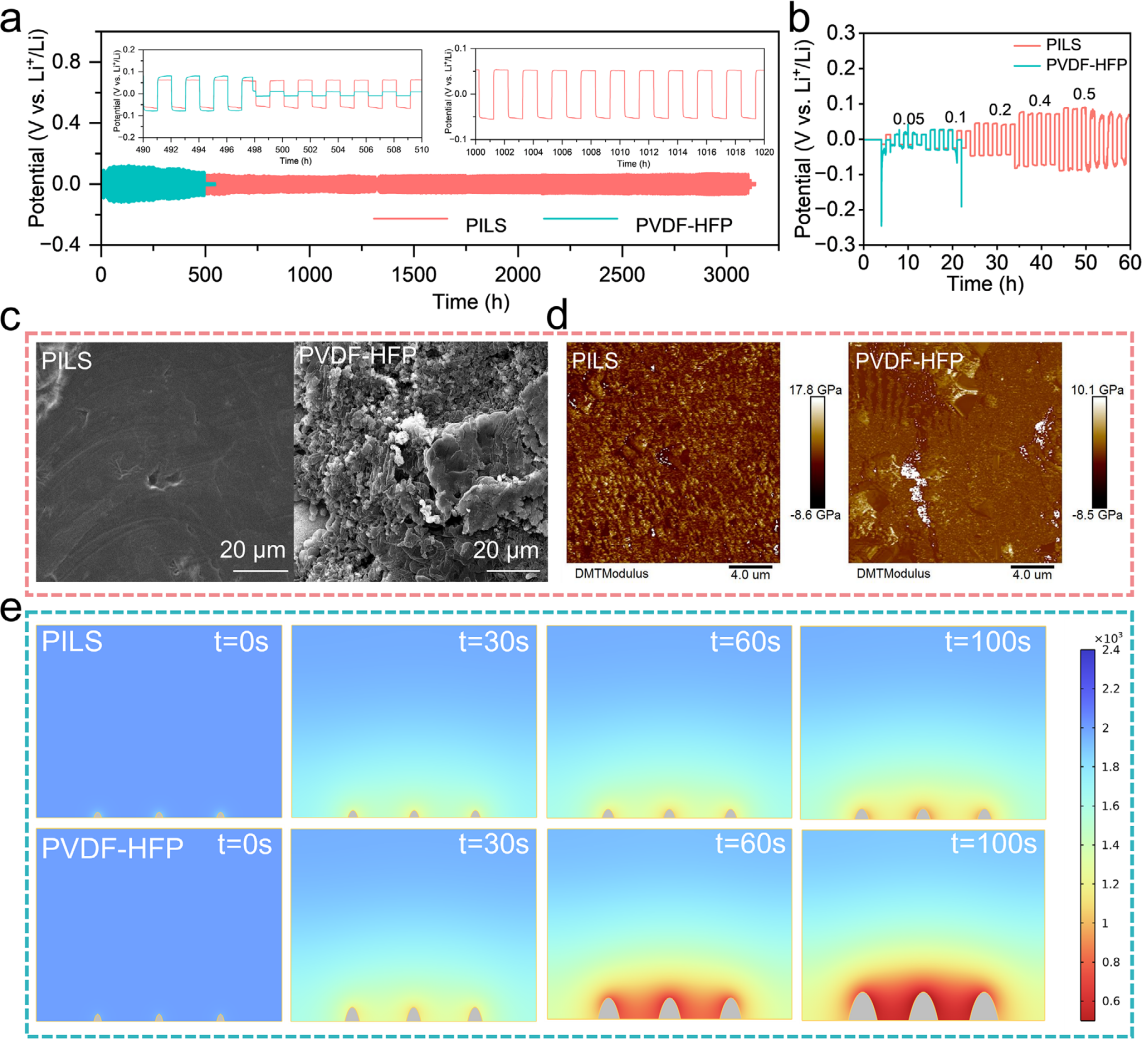
Galvanostatic cycles for electrolytes in symmetric Li cells. a) PVDF‐HFP and PILS with a constant current density of 0.1 mA cm^−2^ at 25 °C. The inset is the detailed voltage plateau of PVDF‐HFP and PILS in different cycles. b) The CCD test of Li|PILS|Li and Li|PVDF‐HFP|Li symmetric cells. c) SEM images of Li metal after 50 cycles. d) Surface map of Young's modulus of topological Li metal after 50 cycles. e) Finite element method simulation of Li^+^ concentration profiles and Li‐metal deposition at the selected simulation time of Li electrodes with PILS and PVDF‐HFP.

The electrochemical polarization and interfacial stability were further evaluated in symmetric Li cells. As shown in **Figure** **4**a, the symmetric Li cell equipped with PILS exhibited strikingly stable voltage profiles over an extended period of 3000 h under a current density of 0.1 mA cm^−2^ at 25 °C, without noticeable voltage oscillation. This impressive electrochemical stability underscores the exemplary interfacial compatibility exhibited between PILS and the lithium metal anode, facilitating homogeneous lithium nucleation and dendrite‐free deposition. In contrast, the symmetric Li cell with PVDF‐HFP could only maintain steady operation for 500 h before experiencing a sudden drop in polarization voltage under identical conditions. Furthermore, electrochemical impedance spectroscopy (EIS) was exploited to analyze the symmetric cells with PILS and PVDF‐HFP after 10, 50, and 100 cycles, as shown in Figure S15 (Supporting Information). The Li/PILS/Li cell revealed negligible increases in charge transfer resistance, whereas the Li/PVDF‐HFP/Li cell showed a gradual escalation with each subsequent cycle. Integration of PILS within the asymmetric Li/SSEs/Cu cell configuration manifested extraordinary interfacial stability and exceptional cycling durability (Figure S16, Supporting Information). The galvanostatic cycling profiles (Figure 4b) reveal that PILS‐modified symmetric cells sustained stable electrochemical operation at markedly increased current densities (0.4 mA cm^−2^), whereas the PVDF‐HFP counterparts displayed operational limitations, maintaining electrochemical stability only under reduced current loads (0.1 mA cm^−2^). The elevated critical current density (CCD) exhibited by PILS can be attributed to its superior mechanical robustness coupled with the presence of highly ordered, interconnected ionic transport pathways, which synergistically facilitate uniform lithium electrodeposition and dissolution kinetics. Furthermore, the homogeneous dispersion of LGPS particles within PVDF‐HFP results in a compact and dense electrolyte architecture. Conversely, the stochastic pore distribution characteristic of PVDF‐HFP systems compromises efficient Li^+^ transport kinetics, resulting in anisotropic metal deposition morphologies and non‐uniform stripping behavior during cycling. Such irregularities can compromise the integrity of the solid electrolyte interface (SEI) on the Li metal surface, hastening the growth of Li dendrites and posing a considerable safety hazard. Figure 4c demonstrates pronounced architectural heterogeneity among the electrode interfaces when subjected to distinct electrolyte membrane configurations. The lithium metal interface subjected to the PILS‐mediated environment exhibited remarkable morphological preservation, maintaining pristine surface characteristics devoid of dendritic protrusions or structural anomalies. Oppositely, the anode with PVDF‐HFP showed a rugged, fragmented structure, marked by myriad fissures and formations of “dead Li” symptoms of dendritic growth and subpar electrochemical performance. The strategic integration of LGPS particles within the PILS framework establishes an interconnected ionic transport architecture, facilitating expeditious and isotropic Li^+^ flux, whereas the cooperative effects between the fluoropolymer matrix and inorganic additives promote geometrically regular and thermodynamically stable lithium nucleation and growth. As shown in Figure S17 (Supporting Information), aligned with SEM results, the 3D AFM image of Li deposited in PVDF‐HFP shows a porous architecture with an intricate dendritic pattern and a height variance of ≈6.00 µm. In contrast, Li deposited in the modified electrolyte consists of micron‐sized grains with a much smaller height differential of less than 1.08 µm.

The SEI layer formed with PILS demonstrated a higher Young's modulus than that formed with PVDF‐HFP (Figure 4d), suggesting a more resilient and enduring SEI capable of better withstanding the stress associated with Li deposition and stripping cycles. The enhanced interfacial mechanics of the passivation layer synergistically contribute to prolonged cycle life and mitigated morphological instabilities, as evidenced by the electrochemical performance of PILS‐modified lithium symmetric cells. Finite element method (FEM) simulations elucidated the phenomenon of concentration polarization arising from the different migration velocities of Li^+^ and electrons. Whereas conventional PVDF‐HFP matrices failed to suppress dendrite proliferation, the PILS framework exhibited substantially enhanced Li^+^ accumulation in interfacial regions, consequently promoting homogeneous charge distribution along the lithium metal interface. A comparative visualization illustrates a pronounced reduction in Li dendrite formation with PILS compared with PVDF‐HFP after a 100‐s plating duration (Figure 4e; Figure S18, Supporting Information). Collectively, the cumulative experimental observations elucidate that PILS effectuates a homogeneous spatial distribution of localized current density, thereby orchestrating more uniform lithium electrodeposition morphologies.

**Figure 5:**
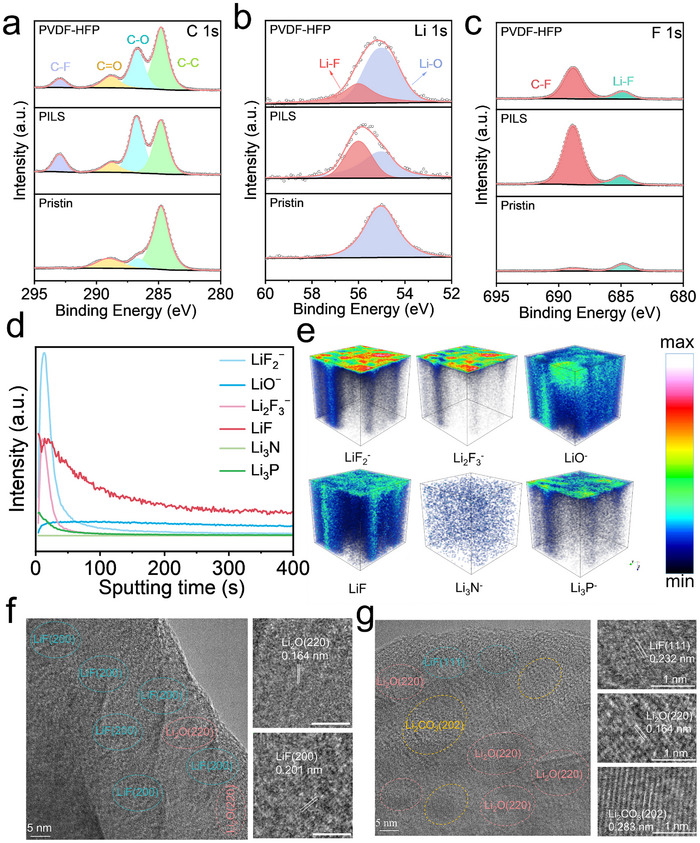
Interfacial and structural characterizations of cycled Li metal anodes. XPS of the SEI with PILS and PVDF‐HFP for a) C 1s, b) Li 1s, and c) F 1s. d) TOF‐SIMS depth profiling of several secondary ion fragments on the SEI with PILS. e) TOF‐SIMS 3D reconstruction of the sputtered volume on the SEI with PILS. Cryo‐electron microscopy (Cryo‐EM) and Fast Fourier transform (FFT) images of cycled Li anodes with f) PILS and g) PVDF‐HFP.

In symmetrical lithium‐metal configurations incorporating PILS, the SEI exhibits markedly enhanced structural integrity and morphological uniformity, consequently facilitating superior electrochemical cycling stability and diminished polarization phenomena. XPS was employed to study the chemical composition on the surface of the Li anode (**Figure** **5**a–c). In the C 1s spectra, the peaks assigned to C‐C, C‐O, C = O, and C‐F originate from the decomposition of LiTFSI. In the Li 1s spectra, peaks at 56.5 and 55.3 eV are attributed to LiF and Li‐O, respectively.^[^ ^29^ ^]^ In the F 1s spectra, two peaks for inorganic LiF and organic C‐F species appear at 685.5 and 687.5 eV, respectively.^[^ ^25^ ^]^ Typically, the LiF moiety concentration within the PILS framework substantially exceeds that observed in PVDF‐HFP matrices, whereas the corresponding Li‐O species exhibit notably diminished abundance. These compositional disparitiesstrongly suggest the predominant role of LiF in facilitating the formation and stabilization of a robust SEI architecture. Its inherently low electronic conductivity helps impede the infiltration of Li dendrites into the electrolyte, ensuring consistent current density distribution throughout cycling.^[^ ^30^ ^]^ Furthermore, time‐of‐flight secondary ion mass spectrometry (TOF‐SIMS) measurements elucidated the spatial distribution of various chemical fragments within the SEI. The inorganic‐rich SEI, characterized by a higher intensity of LiF^2−^, Li^2^F^3−^, LiO^−^, LiF, Li_3_N^−^, and Li_3_P^−^, reflects the composition influenced by PILS. The elevated concentration of inorganic constituents facilitates the development of a more thermodynamically favorable and kinetically efficient lithium electrodeposition process (Figure 5d,e). As shown in Figure 5f, the cycled electrode with PILS clearly displays two types of lattice fringes with distances of ≈1.64 and 2.01 Å, corresponding to the (220) planes of Li₂O and (200) planes of LiF, respectively.^[^ ^31^ ^]^ In comparison, the cycled electrode employing PVDF‐HFP reveals an SEI enriched with a higher concentration of Li_2_CO_3_ (Figure 5g; Figure S19, Supporting Information). The experimental observations herein demonstrate that anodes immersed in the PILS electrolyte, preferentially facilitate the formation of an inorganic‐rich SEI with predominant concentrations of LiF and Li_2_O species. Experimental findings demonstrate that the structurally rigid and highly ordered molecular architecture of PILS orchestrates the formation of a thermodynamically stable and spatially uniform SEI, effectively suppressing dendrite nucleation phenomena and deleterious parasitic reactions at the electrode‐electrolyte interface.

### 2.2 Electrochemical Performance of Li‐O_2_ Batteries with PILS Electrolytes

**Figure 6:**
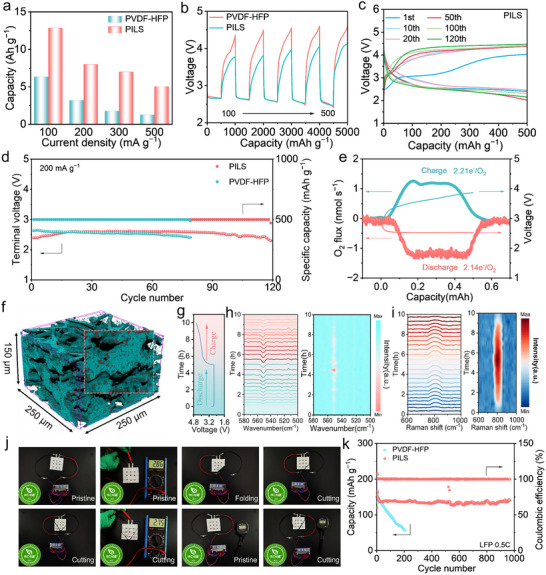
a) Deep discharge profiles of SSLOBs based on PILS and PVDF‐HFP. b) Rate performance of SSLOBs based on PILS and PVDF‐HFP. c) The voltage‐capacity curves of SSLOBs based on PILS with different number of cycles at 200 mA g^−1^ with a cut‐off capacity of 500 mAh g^−1^ d) Voltage versus cycle number on the discharge terminal of SSLOBs based on PILS and PVDF‐HFP. e) In situ DEMS of the discharge and charge processes of SSLOBs based on PILS. f) Computed tomography reconstruction with volume rendering reveals the 3D microstructure of the cathode after discharge. g) Galvanostatic charge/discharge curves of SSLOBs for in situ FTIR and Raman. In situ FTIR h) and Raman i) spectra, taken on the cathodes of SSLOBs based on PILS at different discharge/charge depths to illustrate the reversible Li_2_O_2_ formation/decomposition. j) Solid‐state Li‐O_2_ pouch cells with PILS lighting LED lamps after folding and cutting. k) Capacity retention performance and coulombic efficiency of LiFePO_4_||Li cell assembled with PILS and PVDF‐HFP at 0.5 C.

To systematically evaluate the inherent advantages of PILS architecture, we fabricated SSLOBs incorporating either PILS or PVDF‐HFP as binding matrices, following the protocol delineated in the experimental methodology. The stability of PVDF‐HFP polymer in LOBs has also been validated (Figure S20, Supporting Information). The morphology and microstructure of the as‐prepared reduced graphene oxide (rGO) cathode are presented in Figure S21 (Supporting Information). Compelling electrochemical data, as evidenced in **Figure** **6**a and Figure S22 (Supporting Information), the SSLOBs with PILS achieved a substantial discharge capacity of 12 874 mAh g^−1^ at a current density of 100 mA g^−1^, representing a remarkable 100% enhancement over their PVDF‐HFP counterparts, which delivered 6337 mAh g^−1^ under identical operating conditions. Significantly important, SSLOBs fabricated with PILS maintained an impressive discharge capacity of 5000 mAh g⁻1 when subjected to the elevated current densities of 500 mA g⁻¹, exemplifying the robust electrochemical performance and superior charge transport capabilities of the PILS under high‐rate operating conditions.^[^ ^32^, ^33^, ^34^ ^]^ Consistently, the PILS cell demonstrated a marked reduction in overpotential during constant current discharging/charging (Figure 6b). The galvanostatic intermittent titration technique (GITT) analyses revealed a marked reduction in charging potential for PILS‐functionalized batteries, providing quantitative validation of the enhanced electrochemical performance afforded by PILS modification of the cathode interface (Figure S23, Supporting Information). Figure 6c,d illustrates that the SSLOBs with PILS display enhanced cycling performance compared with PVDF‐HFP and those certain reported electrolytes (Table S1, Supporting Information).^[^ ^35^, ^36^, ^37^, ^38^, ^39^ ^]^ The PILS‐based battery sustained over 120 cycles (exceeding 600 h), whereas the PVDF‐HFP battery only achieved 78 cycles. Furthermore, SSLOBs functionalized with PILS demonstrated markedly superior electrochemical characteristics to their PVDF‐HFP counterparts when subjected to high‐rate cycling conditions, evidencing enhanced charge transport kinetics and improved current handling capabilities (Figure S24, Supporting Information). Also, the battery could cycle stably 468 times with RuO_2_/CNT cathode (Figure S25, Supporting Information). The exhaustive analyses conclusively establish that the strategic implementation of silane coupling agents orchestrates the evolution of an exceptionally resilient organic‐inorganic interfacial architecture, which synergistically potentiates both ionic conductivity and Li^+^ transference coefficients, ultimately culminating in dramatically enhanced electrochemical performance in SSLOBs.

Additionally, the detected amounts of consumed and released oxygen during discharge/charge showed an e^−^/O_2_ ratio approaching 2.0 through in situ differential electrochemical mass spectrometry (DEMS), indicating the high reversibility of the SSLOBs with PILS (Figure 6e; Figure S26, Supporting Information). XRD analysis of the discharge products in LOBs with PILS and PVDF‐HFP detected only the presence of Li_2_O_2_, implying that the choice of SSEs does not influence the formation of discharge products (Figure S27, Supporting Information). Notably, the PILS battery exhibited a pronounced Li_2_O_2_ peak, indicating higher discharge capacity and more discharge product formation. Additionally, the formation and decomposition of Li_2_O_2_ are well reversible during discharge and recharge (Figure S28, Supporting Information). Subsequently, the cathode surface showed thin film‐like discharge remnants that largely dissipated following the recharging process (Figure S29, Supporting Information). Following deep discharge, the 3D rendered image derived from computed tomography (CT) demonstrate a uniform layering of Li_2_O_2_ discharge products on the cathode framework, which are intimately interconnected and predominantly form along the fibrous carbon substrate (Figure 6f). This observation suggests that the regions enriched with the three‐phase interface are propitious for Li_2_O_2_ nucleation. Despite the dense accumulation of discharge products in Figure S30 (Supporting Information), their volumetric proportion within the entire structure remains modest. Nevertheless, the extensive distribution of these products confers a larger cumulative interfacial area, which is advantageous for the subsequent decomposition processes. The robust interfacial contact between the cathode and electrolyte ensures efficient lithium ion and electron transport, which is instrumental in achieving a high‐flux reactive interface and, consequently, enhances the reaction kinetics. Complementing these analyses, XPS provided compelling evidence of reversible formation and decomposition of Li_2_O_2_ (Figure S31, Supporting Information). The Li 1s peak at 55.0 eV and the 56.1 eV correlate to Li_2_O_2_ and the Li‐deficient phase Li_2‐x_O_2_, respectively, persisting throughout the electrochemical cycling process.^[^ ^40^ ^]^ To quantitatively analyze the discharge products, TiOSO_4_‐based UV/vis chemical titration was applied (Figure S32, Supporting Information).^[^ ^20^ ^]^ A high Li_2_O_2_ yield of up to 92.1% was obtained for SSLOBs with PILS, and a residual Li_2_O_2_ yield of 1.5% was calculated after the charging process, further validating the high reversibility and efficiency of the battery system. The in situ FTIR and Raman analyses of the discharge‐charge cycle further corroborate the high reversibility of the discharge products, as illustrated in Figures 6g–i and Figure S33 (Supporting Information). The detailed spectroscopic data unveils a gradual increase in the peak area associated with Li₂O₂ during the discharge phase, followed by a subsequent reduction post‐charging (Figure 6h). In the FTIR spectrum, the peaks at 545 cm^−1^ are attributed to Li_2_O_2_.^[^ ^41^ ^]^ For the Raman spectra, the band at 810 cm^−1^, characteristic of Li_2_O_2_, exhibited heightened intensity in tandem with the increased discharge capacity, attributable to the O‐O stretching mode of Li_2_O_2_ (Figure 6i).^[^ ^42^, ^43^ ^]^

To visually evaluate the safety of PILS, a LOB with a blue light‐emitting diode (LED) was fabricated (Figure 6j). The pouch cell, even after being subjected to folding and cutting, maintained the illumination of the LED lamps. These rigorous mechanical analyses authenticate the structural resilience of the LOBs pouch cell configuration under dynamic stress parameters, substantiating both its safety protocols and operational stability (Figure S34, Supporting Information). High‐resolution infrared thermography revealed a homogeneous thermal distribution across the cell surface, signifying exceptional thermodynamic stability and efficient heat management, which are indicative of superior electrochemical performance (Figure S35, Supporting Information). Capitalizing on the distinctive solution‐processability attributes of PILS, we engineered a Li|PILS|LiFePO_4_ electrochemical assembly. The resultant cell demonstrated exceptional electrochemical durability, sustaining robust performance through 968 galvanostatic cycles at a charge‐discharge rate of 0.5 C (Figure 6k; Figure S36–S38, Supporting Information). Meanwhile, Li‐CO_2_ electrochemical cells incorporating PILS as the electrolyte medium demonstrated viable operational functionality (Figure S39, Supporting Information). Experimental evidence has established PILS as a highly efficacious medium for Li^+^ transport phenomena, yielding remarkable electrochemical characteristics in both SSLOBs and lithium metal‐based energy storage architectures. Implementation of this ionic transport medium within these electrochemical configurations demonstrates synergistic benefits, manifesting in both amplified energy density metrics and enhanced operational safety protocols, while concurrently extending cycle stability—attributes that collectively position this material as a compelling prospect for advanced energy storage paradigms.

## 3 Conclusion

The fabrication of a mechanically compliant and densified PILS membrane with a thickness of 40 µm, accomplished through the strategic incorporation of organosilane coupling agents into conventional hybrid organic‐inorganic composite matrices, constitutes a notable breakthrough in solid‐state electrochemical energy storage systems. This pioneering methodology demonstrates a synergistic integration of enhanced ionic transport properties, exhibiting a conductivity of 1.05×10^−4^ S cm^−1^, coupled with an exceptional Li^+^ transference coefficient of 0.47. The augmented interfacial cohesion between organic and inorganic moieties within the PILS architecture, mediated through robust covalent linkages established via in situ coupling mechanisms, engenders exceptional interfacial compatibility and uniform phase distribution, consequently optimizing lithium‐ion transport kinetics. Meanwhile, the symmetrical Li batteries with PILS displayed excellent cycling performance of surpassing 3000 h at 0.1 mA cm^−2^. Concurrently, PILS‐based LOBs present an outstanding high specific capacity of 12 874 mAh g^−1^, with the ability to consistently endure over 120 cycles without deterioration. Multi‐modal in situ characterization techniques, including DEMS, vibrational spectroscopy in both the mid‐infrared and Raman regions, have elucidated the mechanistic underpinnings of reversible redox processes within PILS‐architected SSLOBs, unambiguously confirming the cyclical generation and dissolution of Li_2_O_2_ species during galvanostatic cycling operations. In addition, pouch cells significantly amplifies the practical viability of these systems in the realm of high‐energy LOB applications. The elucidated molecular‐level understanding of the hierarchical interactions at organic‐inorganic interfaces provides insights into interfacial engineering paradigms, facilitating the strategic development of advanced CSEs that synergistically enhance both safety metrics and electrochemical compatibility—critical parameters for the evolution of SSLOBs and emerging energy storage platforms.

## 4 Experimental Section

All experimental details are shown in the Supporting Information.

## Conflict of Interest

The authors declare no conflict of interest.

## Supporting information



Supporting Information

## Data Availability

The data that support the findings of this study are available on request from the corresponding author. The data are not publicly available due to privacy or ethical restrictions.

## References

[1] P. G. Bruce , S. A. Freunberger , L. J. Hardwick , J.‐M. Tarascon , Nat. Mater. 2012, 11, 19. 10.1038/nmat3191 22169914

[2] F. Li , T. Zhang , H. Zhou , Energy Environ. Sci. 2013, 6, 1125.

[3] S. Xia , X. Wu , Z. Zhang , Y. Cui , W. Liu , Chem 2019, 5, 753.

[4] Y. Su , X. Rong , H. Li , X. Huang , L. Chen , B. Liu , Y. S. Hu , Adv. Mater. 2023, 35, 2209402. 10.1002/adma.202209402 36341499

[5] Y. Xiao , K. Turcheniuk , A. Narla , A.‐Y. Song , X. Ren , A. Magasinski , A. Jain , S. Huang , H. Lee , G. Yushin , Nat. Mater. 2021, 20, 984. 33686276 10.1038/s41563-021-00943-2

[6] Q. Zhang , D. Cao , Y. Ma , A. Natan , P. Aurora , H. Zhu , Adv. Mater. 2019, 31, 1901131. 10.1002/adma.201901131 31441140

[7] S. Kalnaus , N. J. Dudney , A. S. Westover , E. Herbert , S. Hackney , Science 2023, 381, abg5998. 10.1126/science.abg5998 37733866

[8] A. L. Ahmad , U. R. Farooqui , N. A. Hamid , Polymer 2018, 142, 330.

[9] G.‐C. Song , T. Dam , H.‐B. Na , J. Kim , C.‐J. Park , J. Energy Storage 2023, 72, 108744.

[10] S.‐H. Lu , H.‐C. Lu , J. Power Sources 2021, 489, 229431.

[11] H. T. Le , D. T. Ngo , R. S. Kalubarme , G. Cao , C. N. Park , C. J. Park , ACS Appl. Mater. Interfaces 2016, 8, 20710. 27463563 10.1021/acsami.6b05301

[12] A. Gupta , J. Sakamoto , Electrochem. Soc. Interfaces 2019, 28, 63.

[13] Z. Zhang , S. Zhang , S. Geng , S. Zhou , Z. Hu , J. Luo , Energy Storage Mater. 2022, 51, 19.

[14] J. H. Park , M.‐J. Kwak , C. Hwang , K.‐N. Kang , N. Liu , J.‐H. Jang , B. A. Grzybowski , Adv. Mater. 2021, 33, 2101726. 10.1002/adma.202101726 34288151

[15] H. Wang , J. Chen , P. Pang , Y. Bai , Z. Zheng , T. Huang , K. Jiang , Y. Zhao , G. Zhu , H. Xu , J. Power Sources 2024, 599, 234167.

[16] Y. Wei , W. Chen , X. Ge , J. Liang , Z. Xing , Q. Zhang , Z.‐X. Wang , Polymer 2023, 289, 126501.

[17] Y. Jin , Q. He , G. Liu , Z. Gu , M. Wu , T. Sun , Z. Zhang , L. Huang , X. Yao , Adv. Mater. 2023, 35, 2211047. 10.1002/adma.202211047 36906926

[18] K. Pan , L. Zhang , W. Qian , X. Wu , K. Dong , H. Zhang , S. Zhang , Adv. Mater. 2020, 32, 2000399. 10.1002/adma.202000399 32173931

[19] W. Yu , Z. Yu , Y. Cui , Z. Bao , ACS Energy Lett. 2022, 7, 3270.

[20] A. Kondori , M. Esmaeilirad , A. M. Harzandi , R. Amine , M. T. Saray , L. Yu , T. Liu , J. Wen , N. Shan , H.‐H. Wang , A. T. Ngo , P. C. Redfern , C. S. Johnson , K. Amine , R. Shahbazian‐Yassar , L. A. Curtiss , M. Asadi , Science 2023, 379, 499. 36730408 10.1126/science.abq1347

[21] Z. Li , H.‐M. Huang , J.‐K. Zhu , J.‐F. Wu , H. Yang , L. Wei , X. Guo , ACS Appl. Mater. Interfaces 2019, 11, 784. 30525410 10.1021/acsami.8b17279

[22] H. Zhang , F. Chen , O. Lakuntza , U. Oteo , L. Qiao , M. Martinez‐Ibañez , H. Zhu , J. Carrasco , M. Forsyth , M. Armand , Angew. Chem., Int. Ed. 2019, 58, 12070. 10.1002/anie.201905794 PMC6771960 31259482

[23] X. Wang , L. Ye , C.‐W. Nan , X. Li , ACS Appl. Mater. Interfaces 2022, 14, 46627. 36197083 10.1021/acsami.2c12920

[24] T. Zhou , J. Wang , L. Lv , R. Li , L. Chen , S. Zhang , H. Zhang , B. Ma , J. Huang , B. Wu , L. Chen , T. Deng , X. Fan , Energy Environ. Sci. 2024, 17, 9185.

[25] L. Tang , B. Chen , Z. Zhang , C. Ma , J. Chen , Y. Huang , F. Zhang , Q. Dong , G. Xue , D. Chen , C. Hu , S. Li , Z. Liu , Y. Shen , Q. Chen , L. Chen , Nat. Commun. 2023, 14, 2301. 37085534 10.1038/s41467-023-37997-6 PMC10121557

[26] J.‐F. Liu , Z.‐Y. Wu , F. J. Stadler , Y.‐F. Huang , Angew. Chem., Int. Ed. 2023, 62, 202300243. 10.1002/anie.202300243 36970953

[27] Q. Ruan , M. Yao , J. Lu , Y. Wang , J. Kong , H. Zhang , S. Zhang , Energy Storage Mater. 2023, 54, 294.

[28] J. Zou , H. Kou , R. Chang , X. Zhou , J. Yang , J. Tang , Y. Zhang , J. Power Sources 2024, 614, 235028.

[29] Y. Cao , Y. Sun , C. Guo , W. Sun , Y. Wu , Y. Xu , T. Liu , Y. Wang , Angew. Chem., Int. Ed. 2024, 63, 202316208. 10.1002/anie.202316208 37990065

[30] K. Liu , A. Pei , H. R. Lee , B. Kong , N. Liu , D. Lin , Y. Liu , C. Liu , P.‐C. Hsu , Z. Bao , Y. Cui , J. Am. Chem. Soc. 2017, 139, 4815. 28303712 10.1021/jacs.6b13314

[31] B. Han , Z. Zhang , Y. Zou , K. Xu , G. Xu , H. Wang , H. Meng , Y. Deng , J. Li , M. Gu , Adv. Mater. 2021, 33, 2100404. 10.1002/adma.202100404 33899278

[32] D. Zhang , R. Li , T. Huang , A. Yu , J. Power Sources 2010, 195, 1202.

[33] H. Ouyang , S. Min , J. Yi , X. Liu , F. Ning , Y. Xu , Y. Jiang , B. Zhao , J. Zhang , ACS Appl. Mater. Interfaces 2022, 14, 53648. 36411718 10.1021/acsami.2c13807

[34] Z. Guo , C. Li , J. Liu , Y. Wang , Y. Xia , Angew. Chem., Int. Ed. 2017, 56, 7505. 10.1002/anie.201701290 28524448

[35] J. Wang , Y. Yin , T. Liu , X. Yang , Z. Chang , X. Zhang , Nano Res. 2018, 11, 3434.

[36] K.‐N. Gao , H.‐R. Wang , M.‐H. He , Y.‐Q. Li , Z.‐H. Cui , Y. Mao , T. Zhang , J. Power Sources 2020, 463, 228179.

[37] S. Song , X. Qin , Y. Ruan , W. Li , Y. Xu , D. Zhang , J. Thokchom , J. Power Sources 2020, 461, 228146.

[38] L. Shi , G. Wang , J. Li , M. Wu , Z. Wen , ACS Sustainability Chem. Eng. 2021, 9, 13883.

[39] X.‐X. Wang , X.‐W. Chi , M.‐L. Li , D.‐H. Guan , C.‐L. Miao , J.‐J. Xu , Chem 2023, 9, 394.

[40] Y. Xia , L. Wang , G. Gao , T. Mao , Z. Wang , X. Jin , Z. Hong , J. Han , D.‐L. Peng , G. Yue , Nano‐Micro Lett. 2024, 16, 258. 10.1007/s40820-024-01476-4 PMC11286616 39073728

[41] X. Chi , M. Li , J. Di , P. Bai , L. Song , X. Wang , F. Li , S. Liang , J. Xu , J. Yu , Nature 2021, 592, 551. 33883734 10.1038/s41586-021-03410-9

[42] Y. Qiao , S. Wu , Y. Sun , S. Guo , J. Yi , P. He , H. Zhou , ACS Energy Lett. 2017, 2, 1869.

[43] Y. He , L. Ding , J. Cheng , S. Mei , X. Xie , Z. Zheng , W. Pan , Y. Qin , F. Huang , Y. Peng , Z. Deng , Adv. Mater. 2023, 35, 2308134. 10.1002/adma.202308134 37823718

