# Supplementary material for: Crafting the Organic–Inorganic Interface with a Bridging Architecture for Solid‐State Li‐O2 Batteries
Source: Adv Sci (Weinh). 2025 Jun 19;12(30):e03664. doi: 10.1002/advs.202503664 (PMC12376606; doi:10.1002/advs.202503664)
Supplement: Supplementary file 1 — Supporting Information [file ADVS-12-e03664-s001.docx]

Supporting Information

Crafting the Organic-Inorganic Interface with a Bridging Architecture for Solid-State Li-O_2_ Batteries

*Minghui Li, Kecheng Pan, Dulin Huang, Jing Wu, Zhenzhen Li, Yaying Dou, Zhang Zhang*, Zhen Zhou**

**Experimental Procedures**

**Chemicals**

All chemicals including polyvinylidene fluoride-hexafluoropropylene copolymer (PVDF-HFP, average Mw=400,000, average Mn=130,000, aladdin), Li_10_GP_2_S_12_ (LGPS, Hefei Kejing Material Technology Co., Ltd., 99.99%), 3-isocyanatopropyltriethoxysilane (IPTS, Macklin, 95%), lithium bis((trifluoromethyl)sulfonyl)azanide (LiTFSI, aladdin, 99.95%), polyvinylidene difluoride (PVDF, aladdin, 99.9%), rGO (Hefei Kejing Material Technology Co., Ltd., 99.99%), tetrahydrofuran (THF, Energy Chemical, 99.9%), N-1-methyl-2-pyrrolidone (NMP, Macklin, 99.9%). LiFePO_4_ (LFP, Kejing) and Super P (Kejing) were kept in the vacuum under 80 °C and dried for 24 h.

**Materials Synthesis**

**Preparation of PILS and PVDF-HFP Electrolyte**:

The PILS electrolyte was prepared via an in-situ coupling reaction. PVDF-HFP and LiTFSI (in the mass ratio of 1:1) were first dissolved in THF with a polymer concentration of 10 wt%, followed by mechanical stirring at 60 °C for 24 h to obtain a homogeneous solution. LGPS was then added into the homogeneous solution with the LGPS weight percentage of 10 wt% in the total amount of PVDF-HFP and LGPS. After that, IPTS was added into the solution. The mixed solution was then stirred at 60 °C for 24 h to hold the coupling reaction. After the reaction was fully progressed, the resultant homogeneous solution was cast onto a glass plate with a doctor blade, and then dried in a vacuum oven for 24 h at 60 °C to remove the THF solvent. The NPILS electrolyte was prepared by the same method without adding IPTS. The PVDF-HFP electrolyte was prepared by the same method without adding IPTS and LGPS.

**Characterizations**

XRD patterns were recorded using an Ultima IV X-ray diffractometer (Ultima IV, Rigaku, Japan) with Cu Ka radiation. Morphological analyses were performed on a JEOL JSM-6390 SEM equipped with an element mapping EDS and a JEM-2200FS TEM operated at 200 kV. FTIR spectroscopy was recorded on a Bruker VERTEX70 FTIR spectrometer with KBr pellets. XPS was performed using a ESCALAB 250 spectrometer. Thermogravimetric analysis (TGA) was performed on a TGA 550 thermogravimetric analyzer in N_2_ atmosphere with a heating rate of 10 ℃ min^−1^. Solid-state NMR experiments were conducted with an Agilent 600 DD2 600 MHz NMR spectrometer at room temperature. EIS was performed through a CHI660 Electrochemical workstation and Solartron 1260A Analytical. In situ DEMS analysis was performed with an a-DEMS 100 instrument. Raman spectroscopy was measured on a Thermo Scientific DXR(USA). In-situ IR and Raman use LIB-ATR and LA-Raman from Beijing Scistar Technology Co. Ltd. In situ CT testing was measured on Bruker skysCan2211. AFM was performed with Bruker Dimension Icon. TOF-SIMS was performed using ION-TOF TOF.SIMS5. The cryotransmission electron microscope used Glacios from Thermo Fisher Scientific company.

**Electrochemical Measurements**

**Solid-state Li−O_2_ batteries preparation:**

The cathode films (the loading amount of the rGO is 0.2 mg/cm^2^) were punched into round pellets with a diameter of 12 mm. SSLOBs were assembled in an Ar-filled glove box and sealed in a 2,032-coin battery for testing with a Li anode, a SSEs membrane and a rGO cathode. The charging/discharging curves were collected under oxygen atmosphere through a Land CT2001A battery-testing system.

**Solid-state Li−metal batteries preparation:**

The LiFePO_4_ (LFP) cathodes were prepared using a coating method consisting of 80 wt% of active material, 10 wt% of Super P and 10 wt% of polyvinylidene difluoride (PVDF), the loading is controlled to be around 2 mg cm^−2^.

**Ionic conductivity:** The ionic conductivities of PILS and PVDF-HFP for Li-ion blocking SS/SSEs/SS symmetric batteries were measured through the electrochemical impedance instrument (Solartron 1260A Analytical) with a frequency range of 10^−2^ -10^7^ Hz and a temperature control system. The ionic conductivities (σ) were determined according to the following equation:

$$\sigma=\frac{L}{R_{b}\times S}(1)$$

where L was the pellet thickness, R indicated the resistance, and S presented the contact area with the electrodes. The activation energy (Ea) was calculated from the slope of the Arrhenius plot.

$$\sigma=A\exp(-E_{a}/RT)(2)$$

where A represents the pre-exponential factor, E_a_ signifies the activation energy for ionic conduction, R denotes the Boltzmann constant, and T is the absolute temperature.

**Li-ion transference number:** The Li-ion transference number (t_Li+_) was evaluated using a potentiostatic polarization method at room temperature. The DC flowing through the Li/SSEs/Li symmetric battery and the AC impedance before and after polarization were measured to determine the t_Li+_ value of PILS and PVDF-HFP as follows:

$$t_{Li^{+}}=\frac{I_{s}(\Delta V-I_{0}R_{0})}{I_{0}(\Delta V-I_{s}R_{s})}(3)$$

where I_S_ was the steady-state current, I_0_ indicated the initial current, ∆V presented the applied potential, and R_0_ and R_S_ were the interfacial resistances before and after polarization, respectively.

**LSV:** The electrochemical window of PILS and PVDF-HFP were tested on batteries with a working electrode of stainless and a reference electrode of metallic Li with an electrochemical workstation under a sweep rate of 0.1 mV/s in a voltage range from 3.0 V to 5.0 V (versus Li/Li^+^) at room temperature.

**Simulations and calculations**

**DFT calculations:** The DFT calculations were performed using the Vienna ab initio Simulation Package (VASP).^[1]^ The Perdew-Burke-Ernzerhof (PBE) functional of generalized gradient approximation (GGA) with projector augmented wave (PAW) was applied to describe the electronic structures of materials.^[2, 3]^ An energy cutoff of 400 eV is adopted for the plane-wave basis. The vacuum layers are set to ~40 Å to decouple the interaction between periodic images. The Brillouin zones are sampled using Gamma-centered k-mesh of 1 × 1 × 1. The energy convergence criterion of geometry relaxation is set to 10^–5^ eV. The rest atomic layers and adsorbates are free to relax until the net force per atom is less than 0.05 eV/Å. The DFT-D3 method is used to describe the van der Waals interaction.^[4]^ The VASPKIT code is used for the post-processing of the VASP computational data.^[5]^ The structures were visualized using the VESTA package.^[6]^

The differential charge density is calculated according to, Δρ = ρ_AB_ − ρ_A_ − ρ_B_, where ρ_AB_, ρ_A_, and ρ_B_ represent the total charge density, the substrate charge density, and the surface adsorbate charge density, respectively. Yellow and blue colors indicate the charge accumulation and depletion, respectively.

The adsorption energy is calculated according to the following equation: *E*_ads_ = E_AB_ − E_A_ − E_B_, E_AB_ is the total energy of the structural PVDF-HFP and the linker molecule IPTS together on the substrate LGPS, E_A_ is the energy of PVDF-HFP and the linker IPTS molecule, and E_B_ is the energy of the LGPS base. In another model, E_AB_ is the total energy of the PVDF-HFP molecule adsorbed alone on the substrate LGPS, E_A_ is the energy of the isolated PVDF-HFP molecule, and E_B_ is the energy of the LGPS substrate.

**Governing equations:** The modeling of the ion accumulation-diffusion process was performed in COMSOL Multiphysics software with diluted species transport module and electrical module. Specifically, built a rectangular simulation model with dimensions of 10*8 um, and the distribution of electric field and Li^+^ for the Li anode were predicted with a 2D Nernst-Planck formulation accounting for diffusion and migration in the bulk electrolyte. To demonstrate the initial rough surface effect, three initial protrusions are set on the negative electrode surface to represent the nucleation sites of Li^+^. The transient electrochemical process was considered in this model to understand the dynamics distribution of electric field and Li^+^. The governing equations for anode considering mass balance, species transport, electro-neutrality, and current conservation are given as:

$\frac{\text{∂}\text{c}_{\text{i}}}{\text{∂t}}\text{ + }\text{∇⋅}\text{J}_{\text{i}}\text{= }\text{R}_{\text{i}} (4)$

$$\text{J}_{\text{i}}\text{= -}\text{D}_{\text{i}}\text{∇}\text{c}_{\text{i}} \text{- }\text{z}_{\text{i}}\text{u}_{\text{i}}\text{F}\text{c}_{\text{i}}\text{∇}\text{ϕ}_{\text{l}} (5)$$

$$\sum_{\text{i}} \text{z}_{\text{i}}\text{c}_{\text{i}}\text{= 0}\text{ (6)}$$

$$\nabla\text{⋅}\text{i}_{\text{l}}\text{=F}\sum_{\text{i}} \text{z}_{\text{i}}\text{R}_{\text{i}} (7)$$

where c𝑖 is the concentration, Ji is the mass flux for each species, Ri = $\frac{\text{-}\text{ν}_{\text{i}}\text{i}_{\text{loc}}}{\text{2F}}$ is the electrochemical reaction source term, Di is the diffusion coefficient, ui is the mobility, zi is the charge number, $\text{i}_{\text{l}}\text{= }\sum_{\text{m}} \text{i}_{\text{loc,m}}$is the current density of electrolyte (m indicates the charge transfer electrode reaction), $\text{ϕ}_{\text{l}}$ is the electrolyte potential, $\text{ν}_{\text{i}}$ is the stoichiometric coefficient, iloc is the local current density at the electrode surface, and F is the Faraday constant.

**MD simulation:** Molecular dynamic simulations were carried out with the Gromacs 2024. The Optimized Potentials for Liquid Simulations all atom (OPLS-AA) force fields were adopted for electrolyte. The model assembly is performed using Packmol.

Cubic cells of size 5.26 nm were used for PVDF-HFP: (including 105 LiTFSI, 140 PVDF-HFP). Cubic cells of size 5.67 nm were used for PILS: (including 105 LiTFSI, 140 PVDF-HFP, 5 LGPS and 40 IPTS). A modified velocity-rescaling thermostat was used to maintain the temperature at 298K, and setting the coupling time constant to 0.1 ps. A semi-isotropic Parrinello-Brahmanbaria was used to maintain the pressure at l bar. The Particle Mesh Ewald method was used to deal with electrostatic interactions. The total simulation time is 20 ns to obtain the equal product. The final 2 ns of the production run were used to generate MSD results.

**Results and Discussion**

**
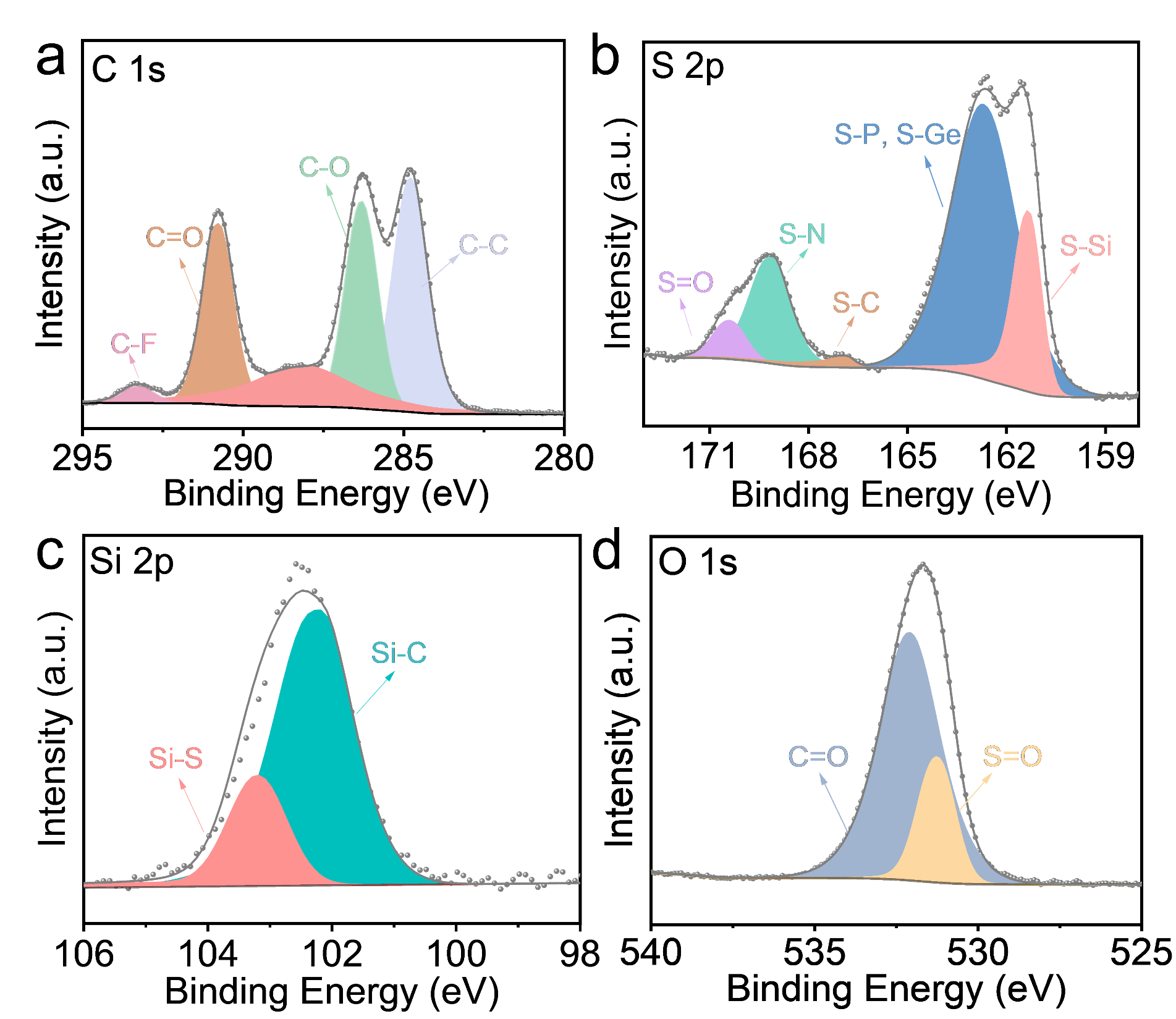
**

Figure S1. XPS spectra for (a) C 1s, (b) S 2p, (c) Si 2p and (d) O 1s of PILS.

As illustrated in Fig. S1d, the O 1s spectrum reveals two distinct peaks at 532.4 and 532.9 eV, signifying the presence of the O=S bond from LiTFSI and the O-C bond from IPTS, respectively.


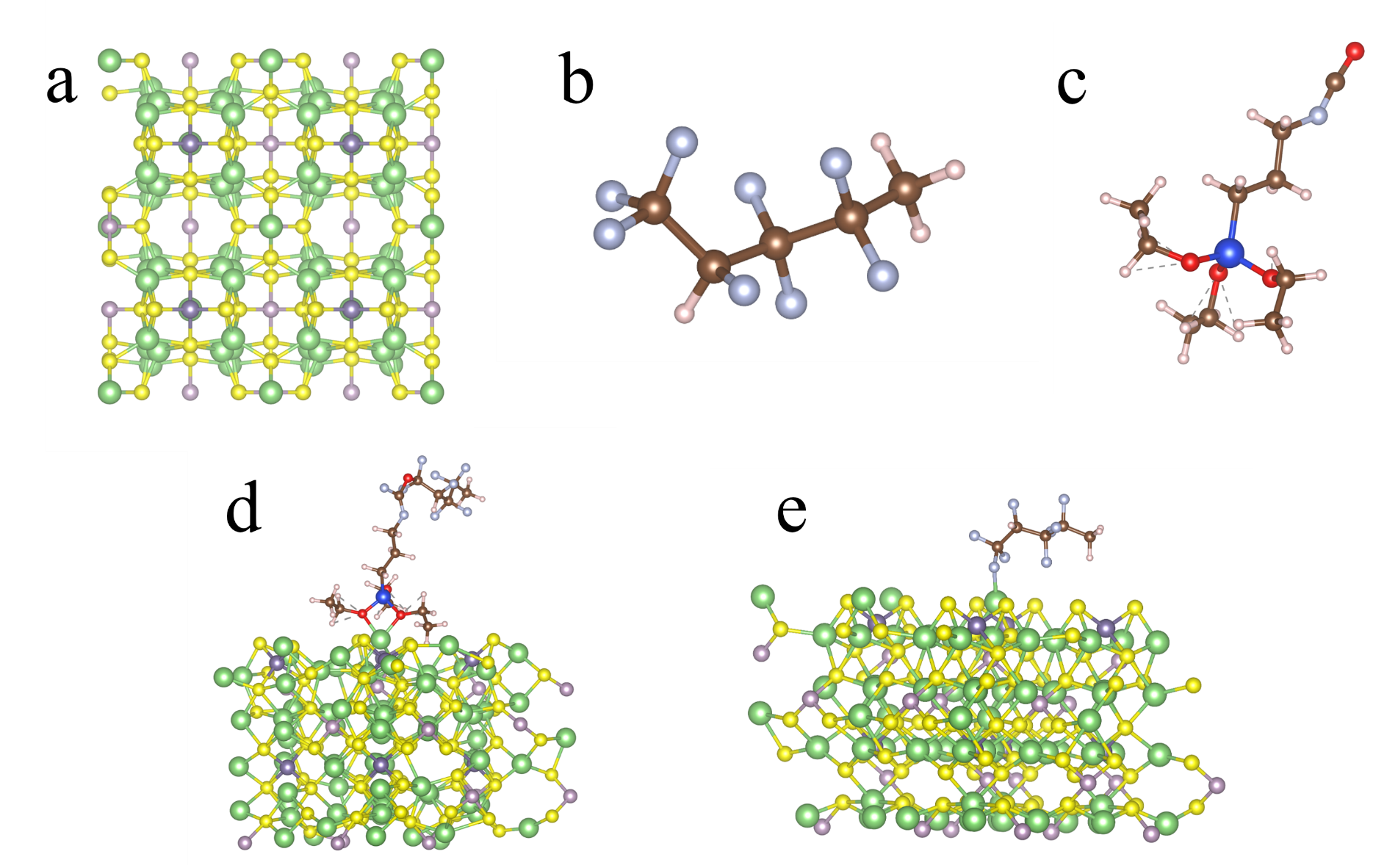


Figure S2. The DFT calculation structure of (a) LGPS, (b) PVDF-HFP and (c) IPTS. The interaction of LGPS/PVDF-HFP structure (d) with IPTS and (e) without IPTS.


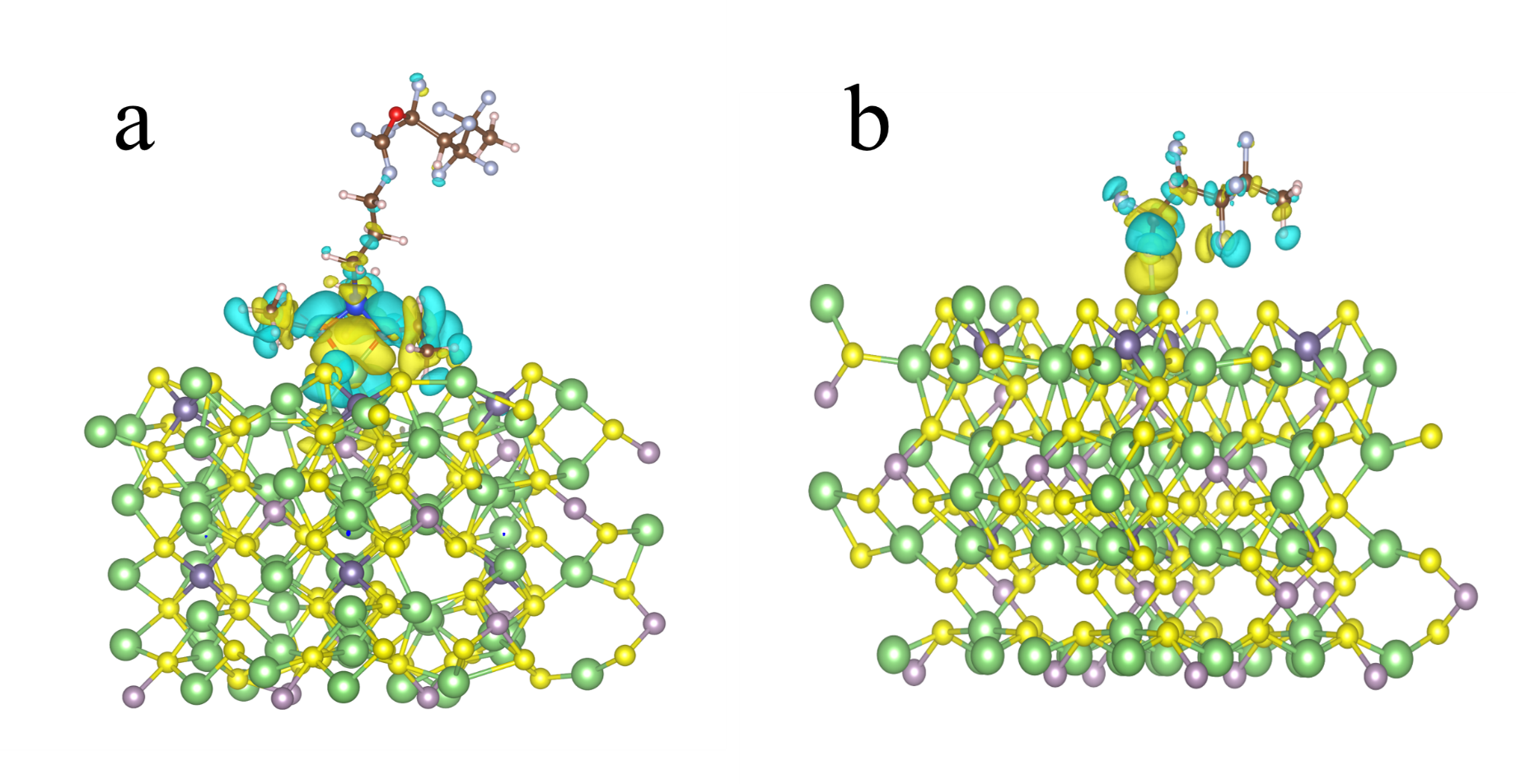


Figure S3. CDD calculation of LGPS/PVDF-HFP structure (a) with IPTS and (b) without IPTS.


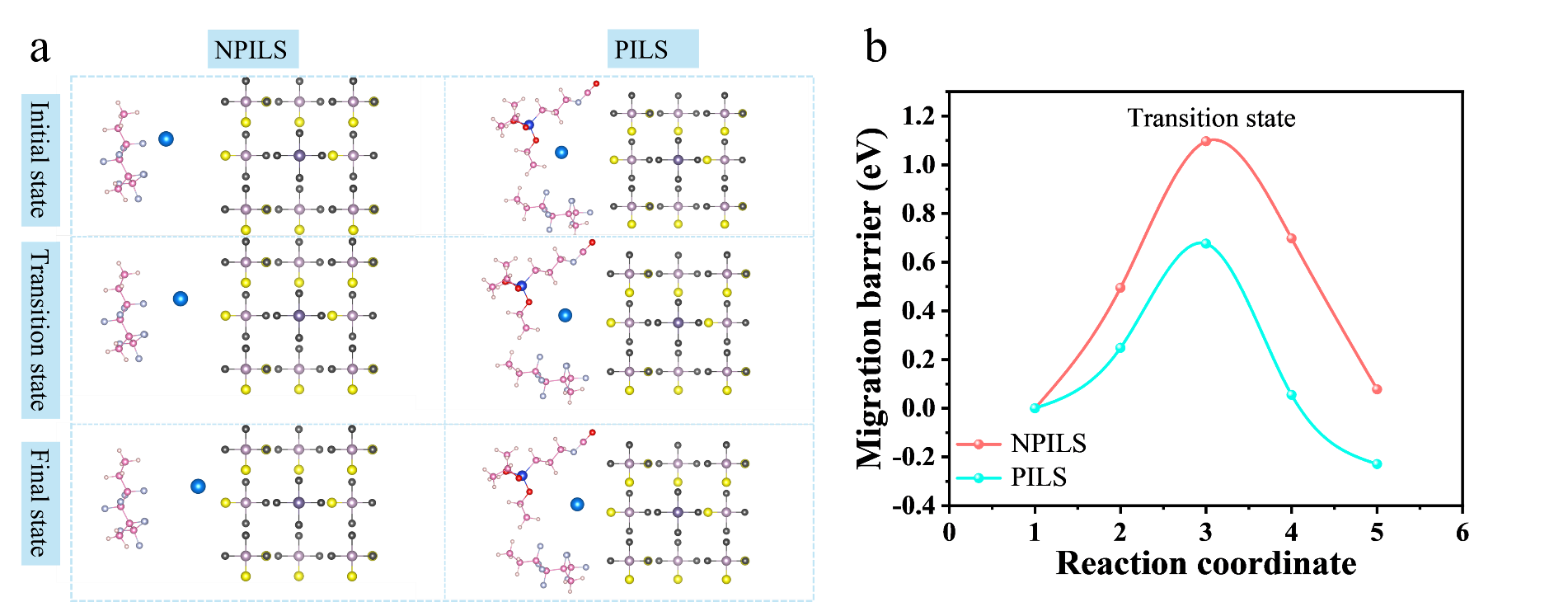


**Figure S4**. (a) Schematic diagram of lithium-ion migration in PVDF-HFP/LGPS at different states in NPILS (PVDF-HFP/LGPS without IPTS) and PILS electrolytes. (b) Lithium-ion migration barriers in two electrolytes.


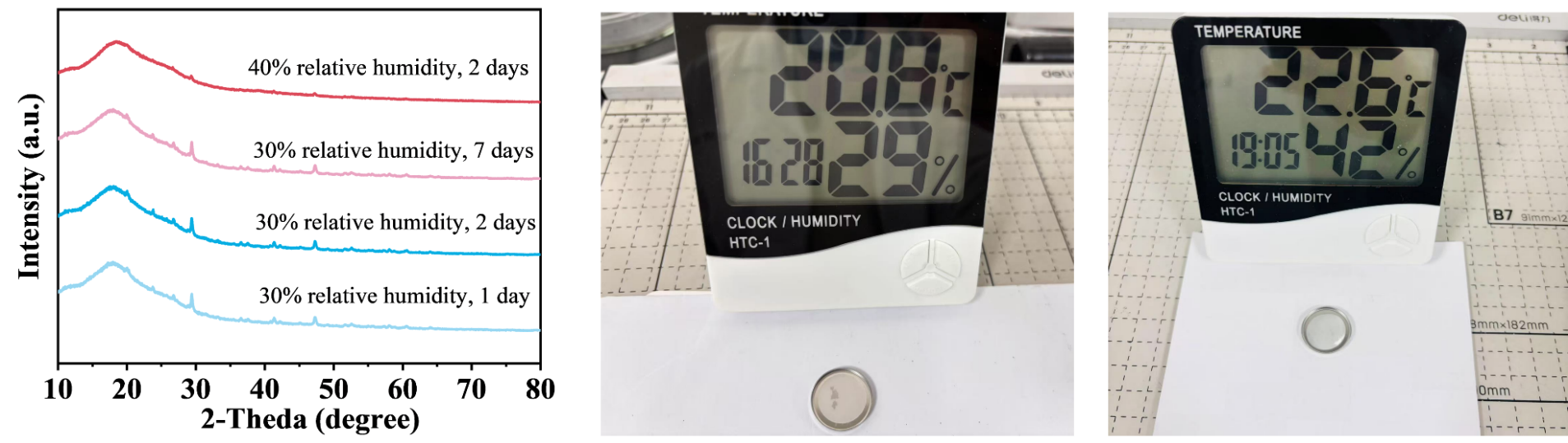


**Figure S5.** XRD patterns of the PILS samples exposed in around 30% and 40% humidity for different durations.


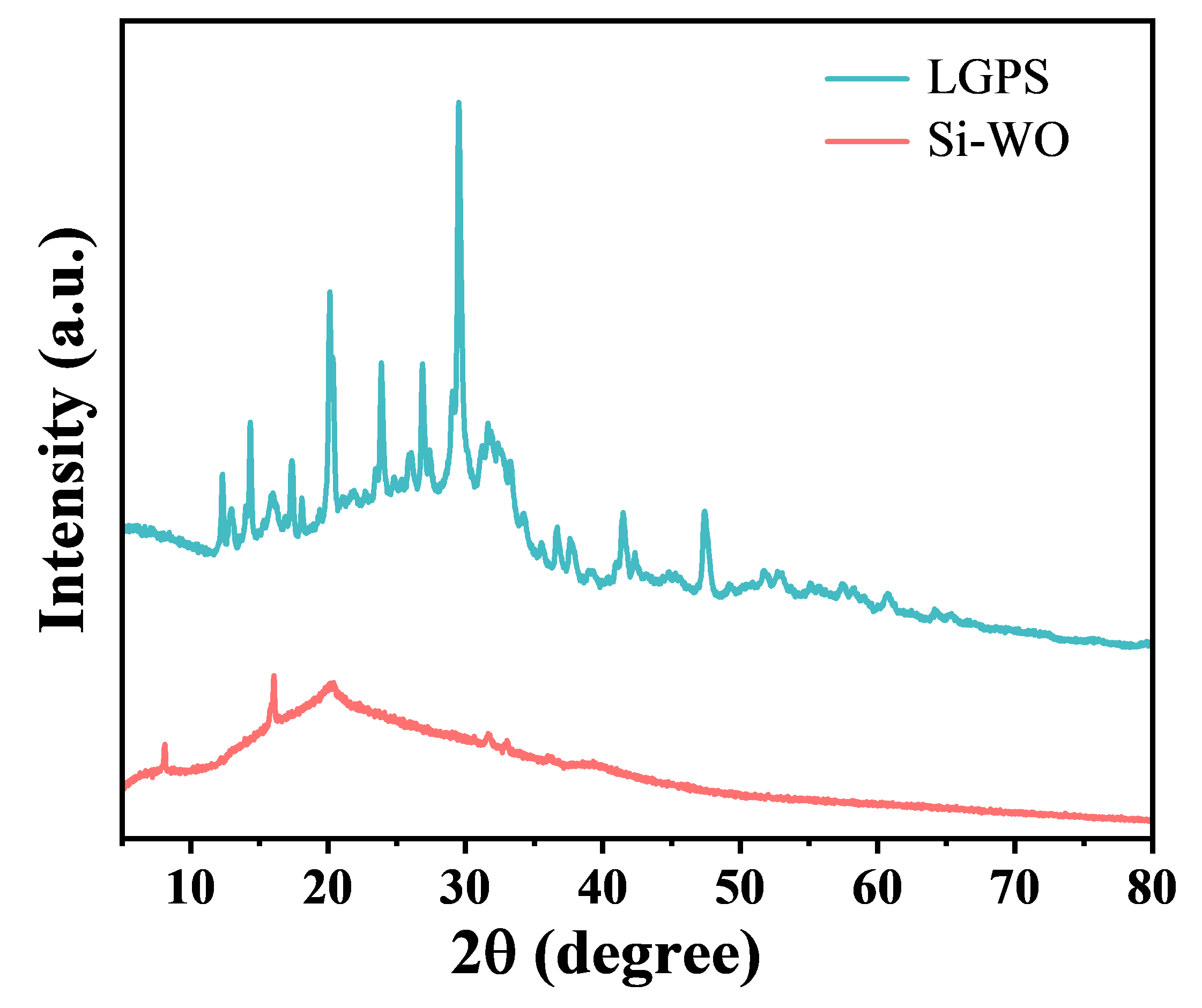


**Figure S6**. XRD pattern of LGPS and PILS without IPTS.


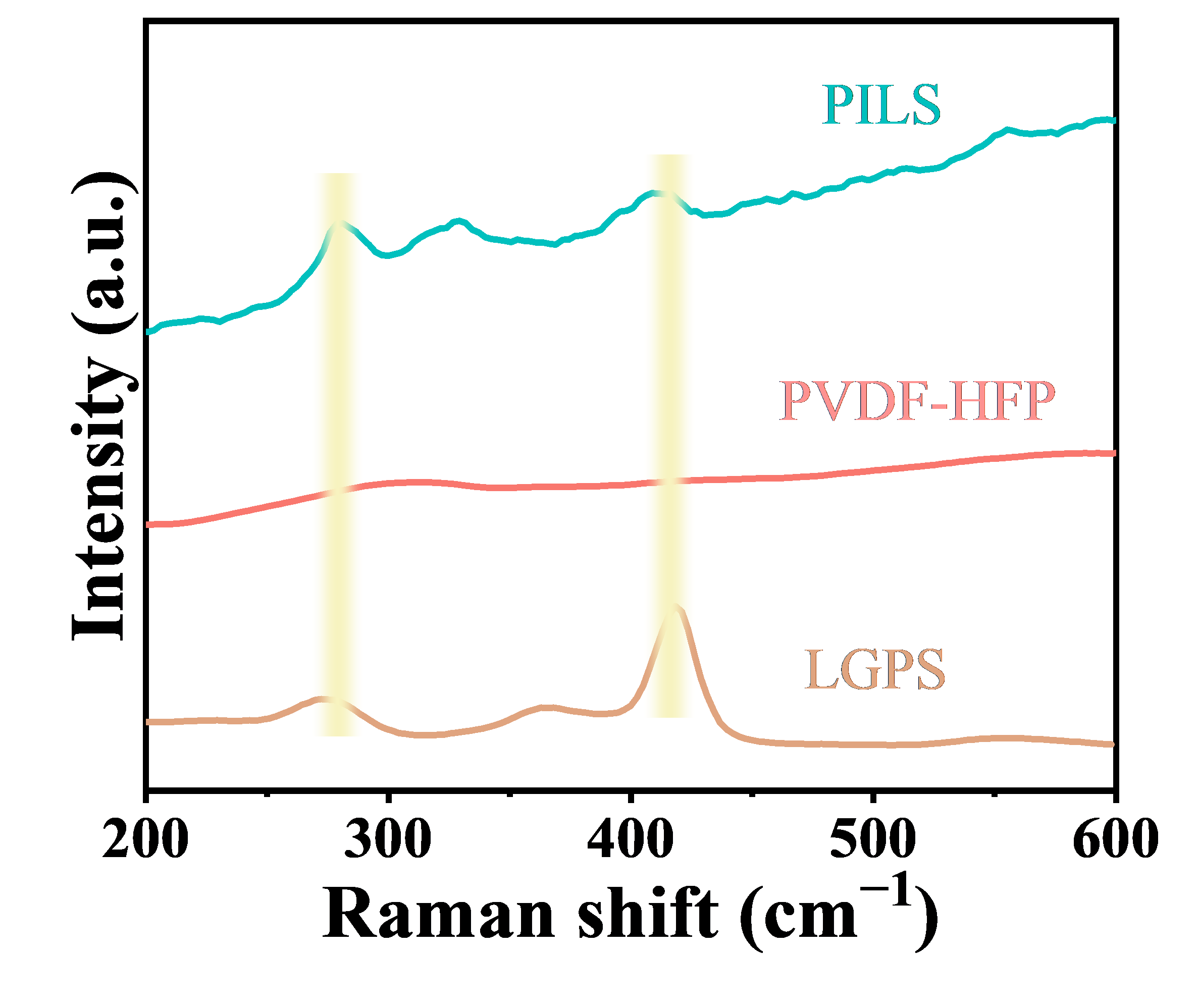


**Figure S7**. Raman spectra of PILS, LGPS powder and PVDF-HFP.

To further investigate the influence of solvents or polymers on the integrity of LGPS, Raman spectroscopy was employed to scrutinize for any unintended side reactions. The Raman spectrum of the PILS, as depicted in **Fig. S7**, exhibits the characteristic peak positions of the PS_4_^3−^ group (420 cm^−1^),^[7]^ emblematic of the LGPS phase. This observation signifies that the local structural integrity of LGPS is preserved within the PILS matrix, corroborating the structural findings from the XRD analysis.


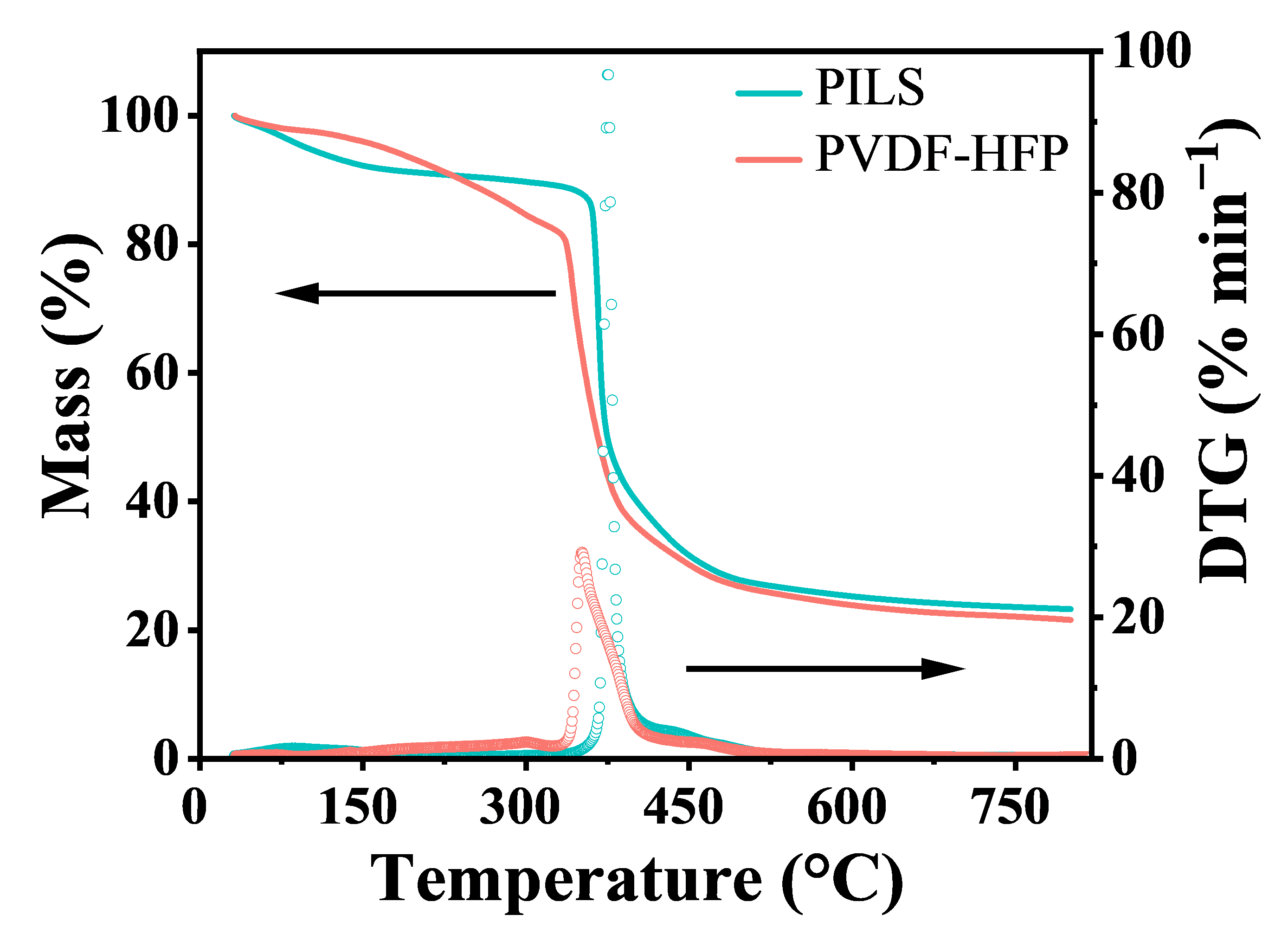


**Figure S8**. TGA and DTG curves of PILS and PVDF-HFP.

Thermal stability is a critical parameter for ensuring the safety of LOBs. The thermogravimetric analysis (TGA) curves depicted in **Fig. S8** reveal that both the PVDF-HFP and the PILS exhibit two distinct weight-loss stages. The initial 10% weight loss temperature for PILS, which is attributed to the evaporation of residual tetrahydrofuran (THF) solvent and additives within the polymer phase, is observed to be slightly higher than that of the PVDF-HFP. The weight loss sharply appearing at about 350°C, slightly higher than that of PVDF-HFP (starting from about 330°C), refers to the thermal decomposition of polymer and lithium salt. These findings underscore the exceptional thermal stability of PILS, which surpasses that of conventional organic liquid electrolytes that are prone to flammability. This enhanced stability suggests that PILS could be a viable candidate for ensuring the stable and safe operation of LOBs across a broader temperature range. The final plateau in the TGA curves, corresponding to about 20% of the remaining weight, is primarily associated with the residual inorganic components of LGPS particles within the PILS matrix and the polymer. This remaining weight provides compelling evidence of PILS’s superior thermal stability and its potential safety advantages, which are particularly beneficial for the robustness and reliability of LOB systems.


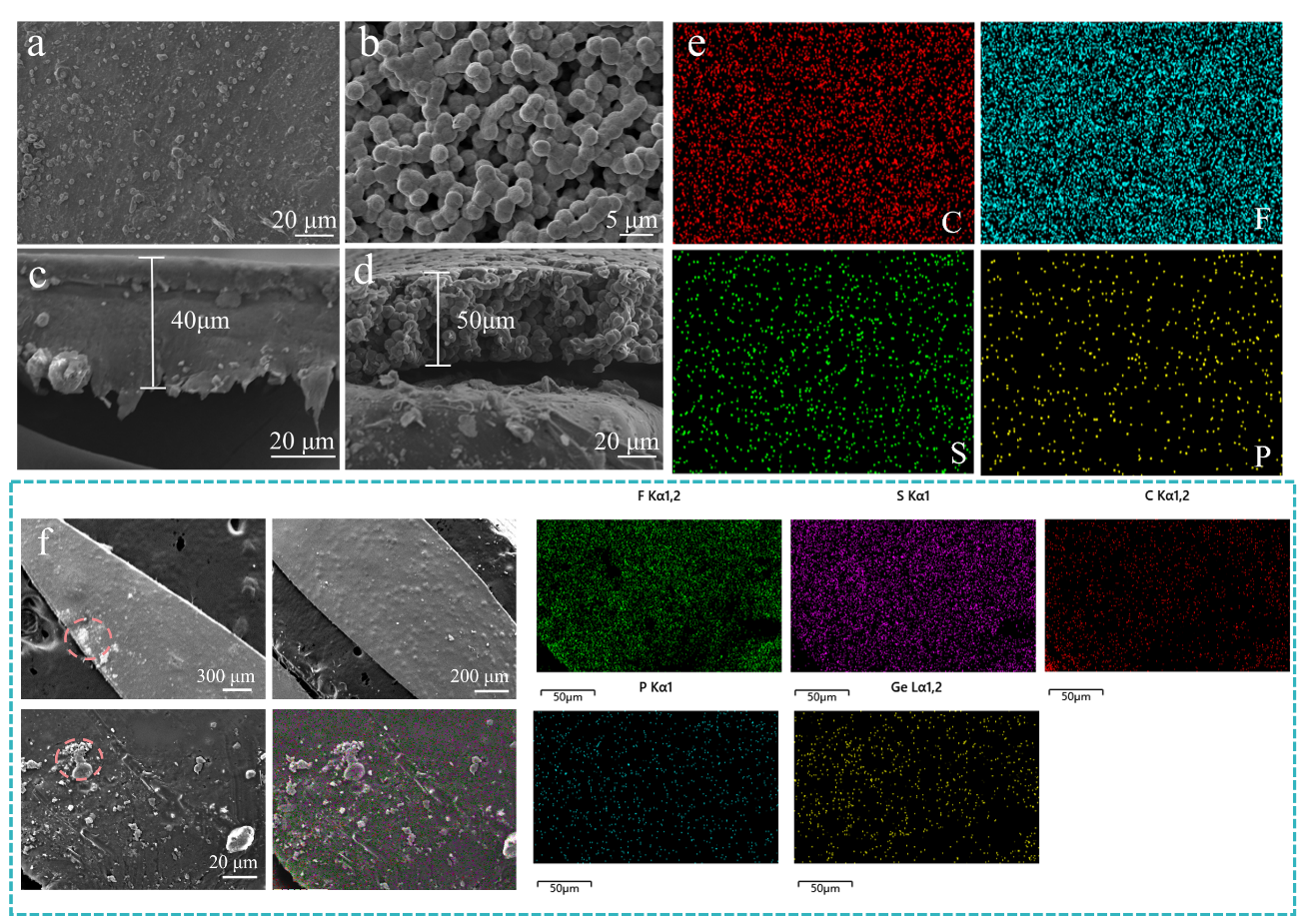


Figure **S9**. SEM image of PILS and PVDF-HFP. The top-view image of (a) PILS and (b) PVDF-HFP. The cross-section-view image of (c) PILS and (d) PVDF-HFP. (e) The corresponding EDS mapping images of PILS. (f) SEM and the corresponding EDS mapping images of PVDF-HFP/LGPS without IPTS. The red circle represents the clustered LGPS.

The top-view SEM image of the PILS in **Fig. S9a** clearly reveals a dense and homogeneous structure, indicating that the sulfide particles are closely packed and firmly integrated within the polymer matrix. The EDS mapping images of C, F, S, and P elements within the PILS, as shown in **Fig. S9e**, further confirm the uniform dispersion of LGPS throughout the PVDF-HFP matrix, with no observable aggregation of nanoparticles. This uniform distribution is crucial for maintaining the electrolyte’s performance and structural integrity. In contrast, the top-view SEM image of the pristine PVDF-HFP, depicted in **Fig. S9b**, exhibits a porous and uneven surface, which is conducive to the infiltration process of LGPS particles. The cross-sectional SEM images presented in **Fig. S9c** and **Fig. S9d** reveal that both the PILS and the PVDF-HFP have a thickness of approximately 40 μm. The PVDF-HFP displays a porous structure, consistent with its surface morphology. Moreover, the cross-sectional view of the PILS highlights a compact structure, indicating that LGPS is well-integrated and adheres seamlessly to the PVDF-HFP. In **Fig. S9f**, where the silane coupling agent is not applied, we can observe that the LGPS ceramic powder is not well-dispersed, leading to the formation of aggregates.


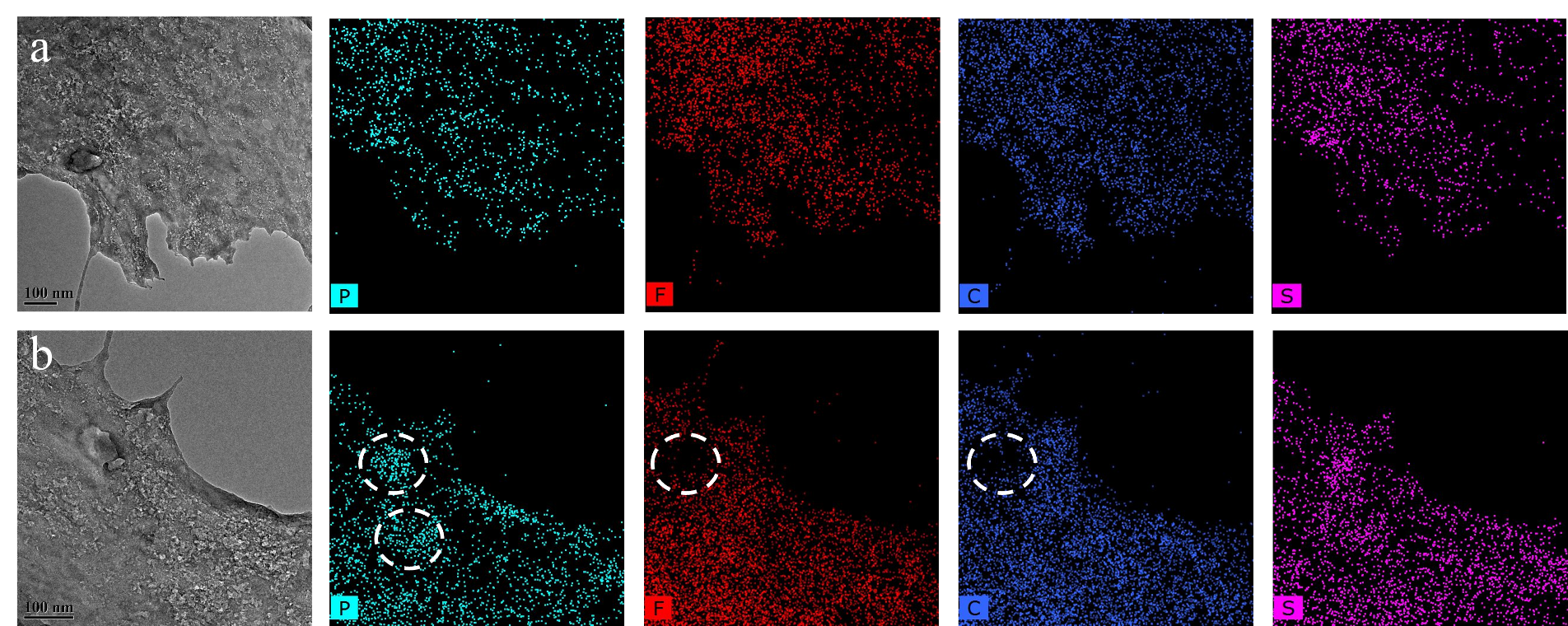

**Figure S10**. TEM and the corresponding EDS mapping images of (a) PILS and (b) PVDF-HFP/LGPS without IPTS. The white circle represents the clustered LGPS.


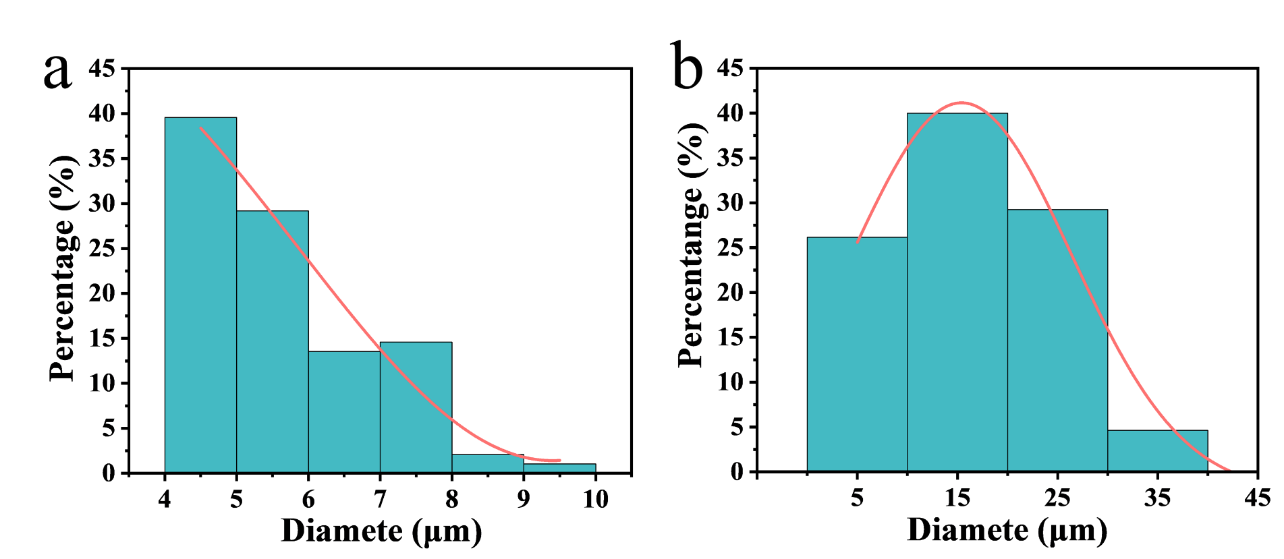


**Figure S11**. Particle size distribution of (a) PILS and (b) PVDF-HFP/LGPS without IPTS.


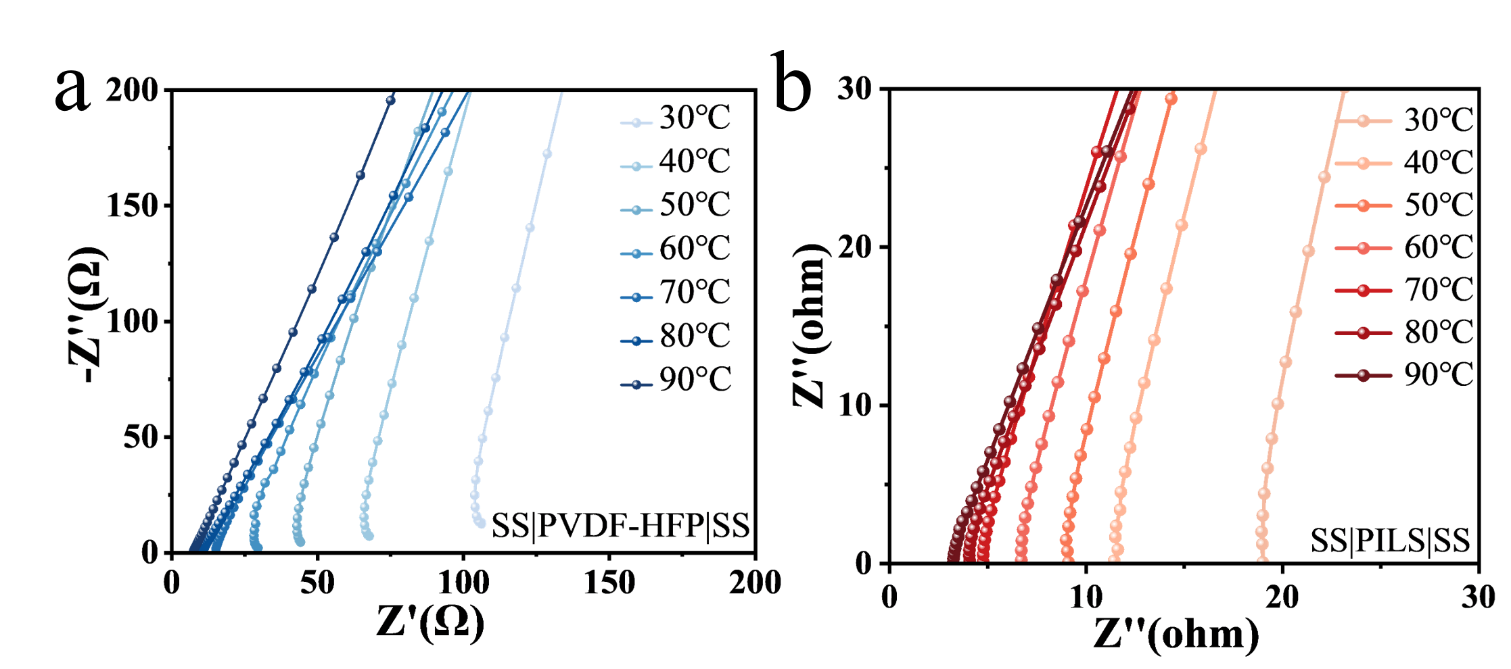


**Figure S12**. EIS spectra of (a) PVDF-HFP and (b) PILS from 30 to 90 °C.


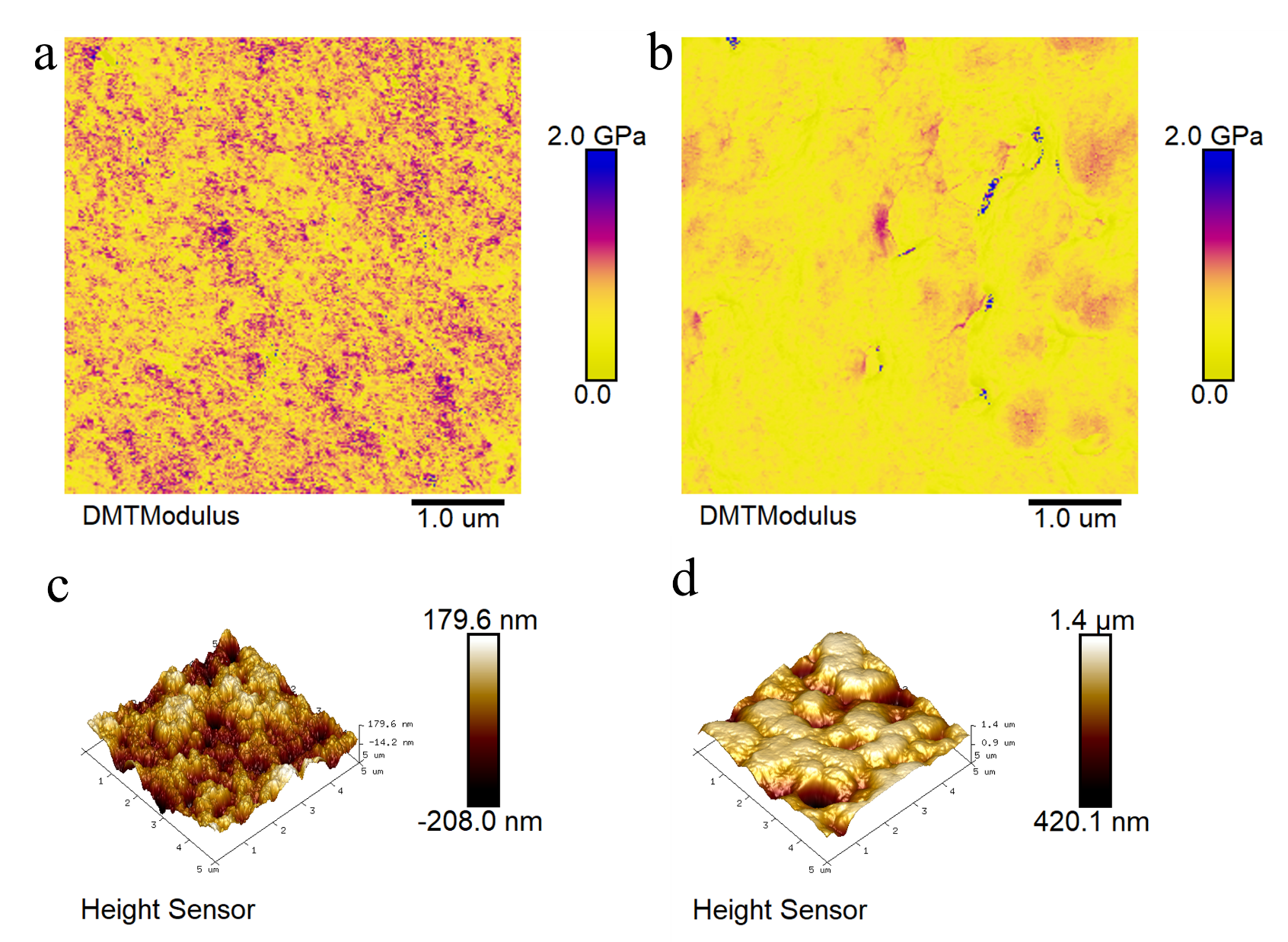


**Figure S13**. Young’s modulus of (a) PILS and (b) PVDF-HFP. The AFM height images of (c) PILS and (d) PVDF-HFP.


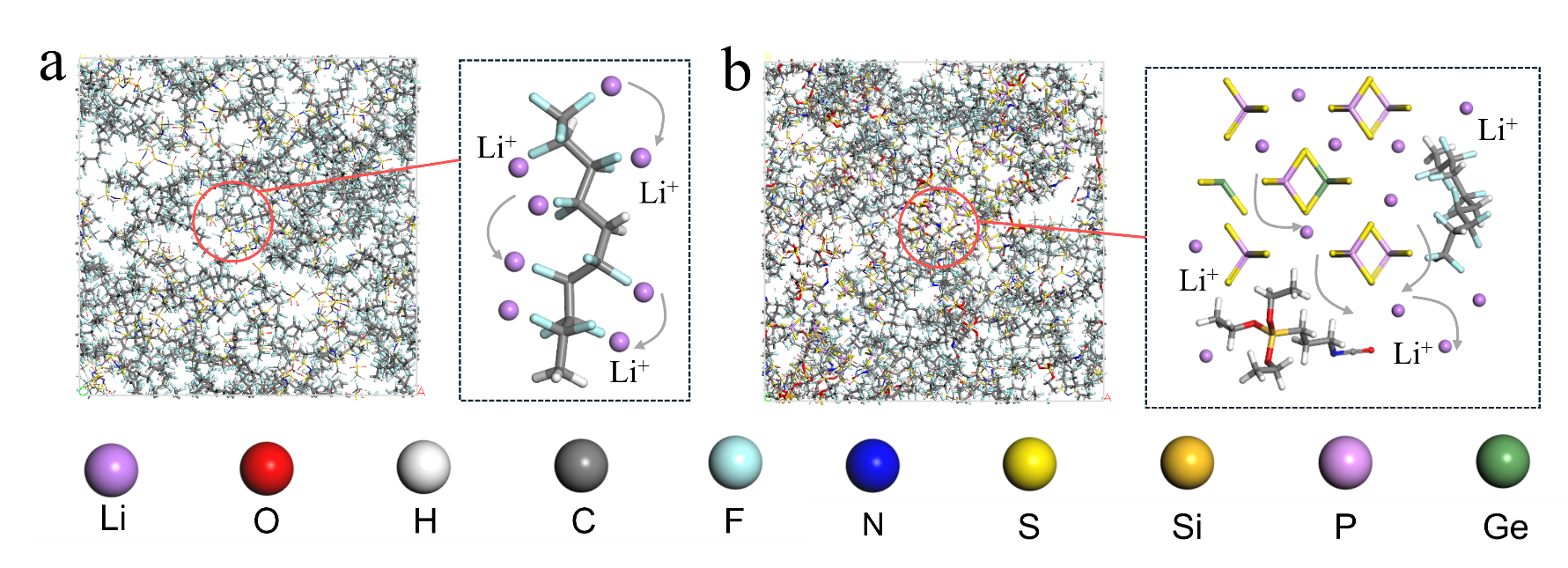


**Figure S14**. Simulated structure of (a) PVDF-HFP and (b) PILS SSEs.


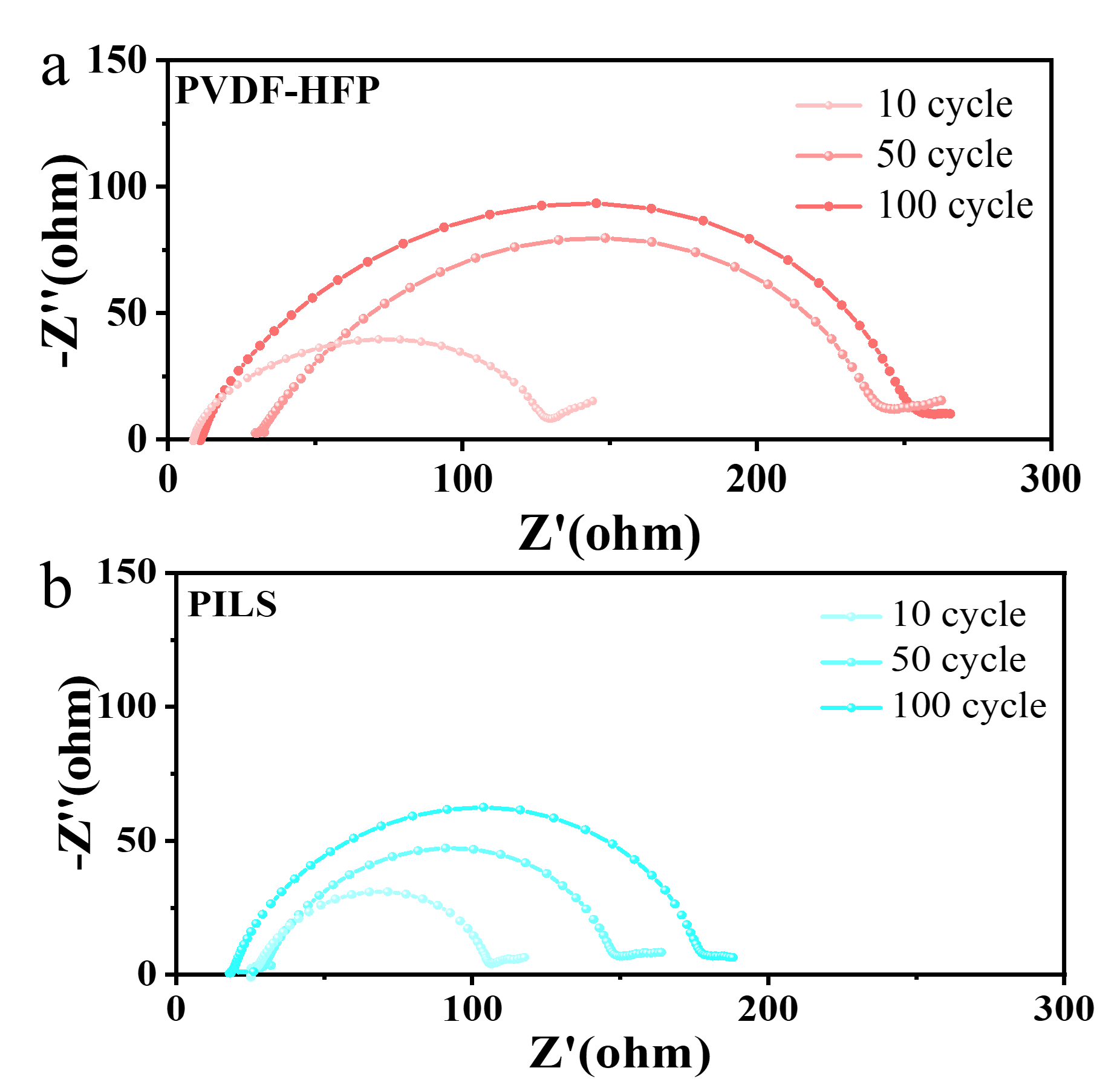


**Figure S15**. Nyquist plots for Li/SSEs/Li symmetric cells after 10, 50, and 100 cycles.

Furthermore, electrochemical impedance spectroscopy (EIS) was employed to analyze the symmetric cells with PILS and PVDF-HFP after 10, 50, and 100 cycles, as shown in **Fig. S15**. The EIS spectra were fitted using equivalent circuits, where the semicircle in the high-frequency region corresponds to the resistance of Li^+^ transport through the interfacial layer, and the semicircle in the mid-frequency region represents bulk Li^+^ transport. The Li/PILS/Li cell exhibited only minor increases in charge transfer resistance, whereas the Li/PVDF-HFP/Li cell showed a progressive increase as the cycling continued. These results demonstrated the enhanced cycling stability of the PILS, which is attributed to the formation of a stable solid electrolyte interphase (SEI) and a uniform Li^+^ flux across the surface of the lithium metal.


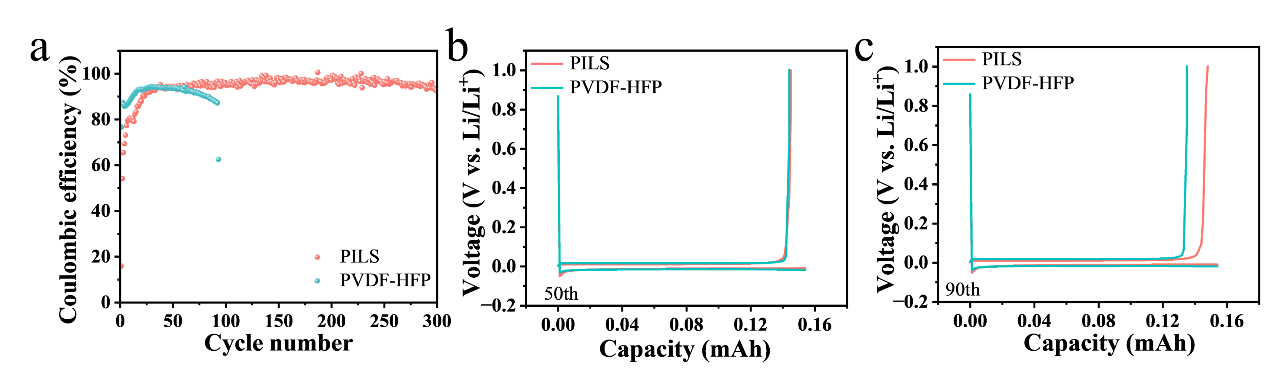


**Figure S16**. Cycling performance of the asymmetric Li||Cu cells. (a) Coulombic efficiencies of PILS and PVDF-HFP as a function of cycle number at 0.1 mA cm^−2^ with 0.1 mAh cm^−2^. (b) The corresponding potential–capacity curves of Li||Cu cells using PILS and PVDF-HFP at (b) 50th and (c) 90th cycles.

The asymmetric Li/SSE/Cu cell, integrated with the PILS separator, demonstrated exceptional stability and cyclability (**Fig. S16**). Specifically, at a current density of 0.1 mA cm^−2^ and an area capacity of 0.1 mAh cm^−2^, the Coulombic efficiency (CE) of the Li/PILS/Cu cell demonstrated consistent performance over 300 cycles, as depicted in **Fig. S16a**. This stability is attributed to the effective suppression of lithium dendrite growth, which is a critical factor in enhancing the longevity of lithium-based batteries. In stark contrast, the CE of the Li/PVDF-HFP/Cu cell, which lacks the advanced features of the PILS, exhibited significant fluctuations and a precipitous decline after only 100 cycles. This decline is primarily due to the uncontrolled growth of lithium dendrites, which can lead to internal short circuits and ultimately compromise the cell's performance and safety.


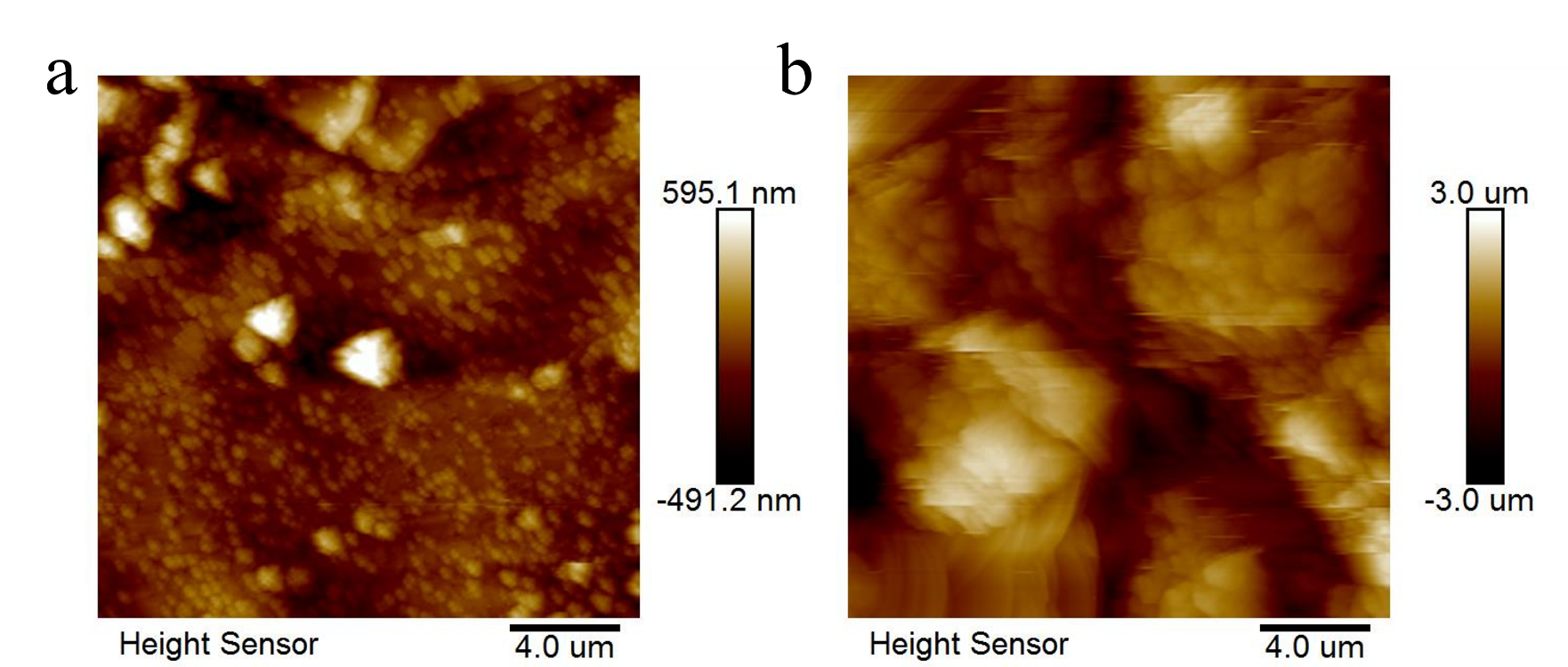


**Figure S17**. The AFM images of the deposited Li metal anode with (a) the PILS and (b) the PVDF-HFP, respectively.


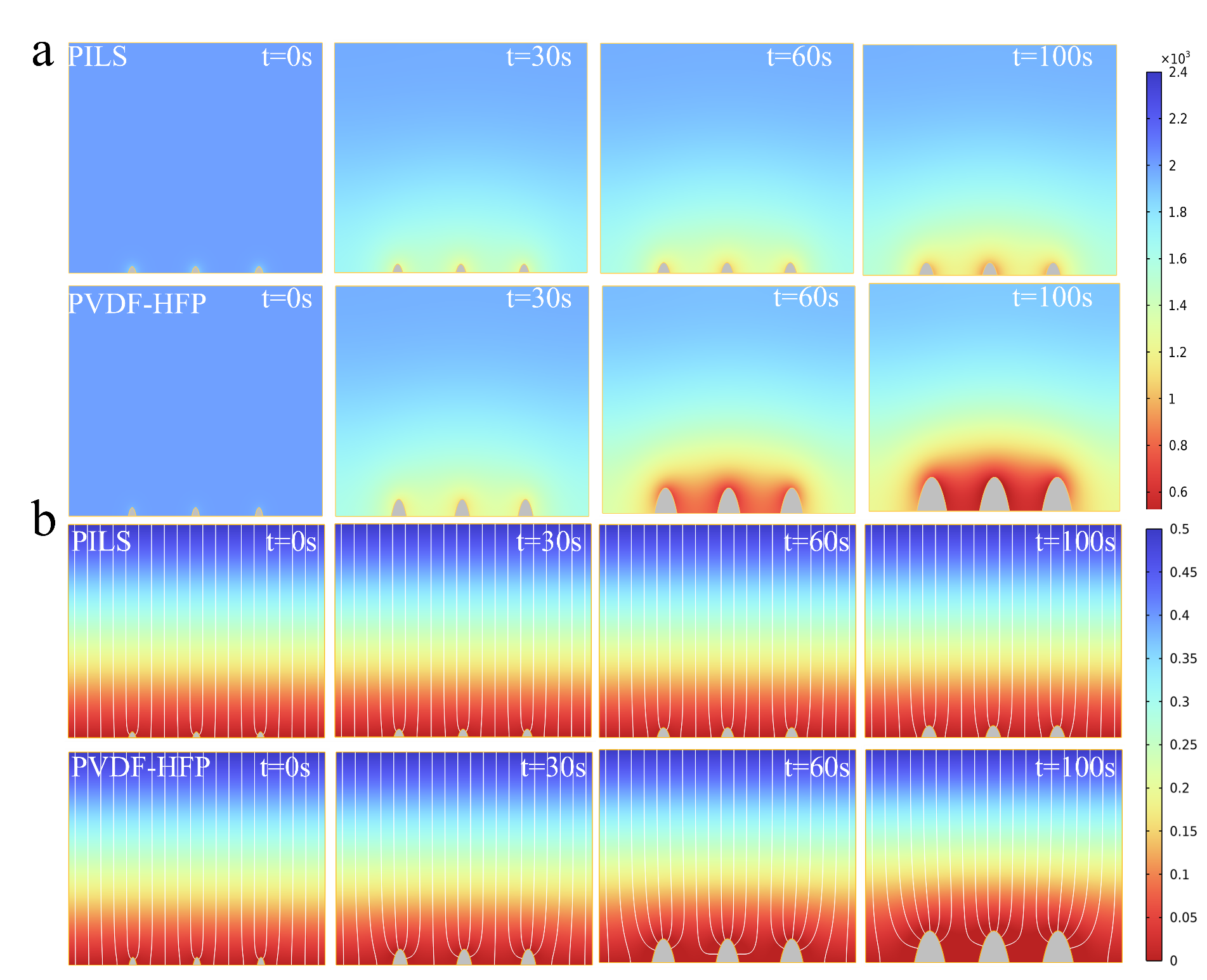


**Figure S18**. FEM simulation of (a) Li^+^ concentration profiles and (b) electric potential distribution at the selected simulation time of Li electrode with PILS and PVDF-HFP.


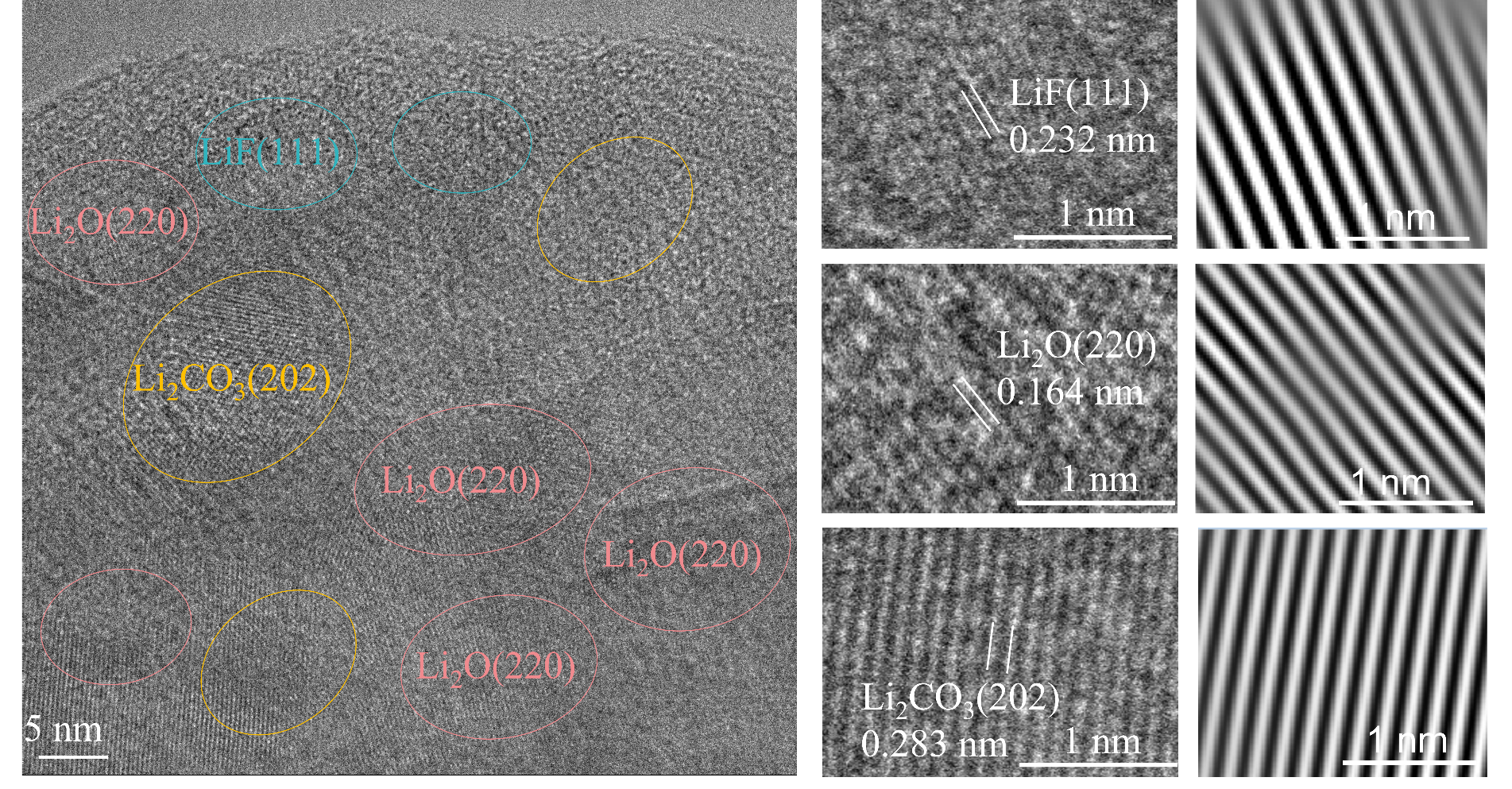


**Figure S19**. TEM and FFT images of cycled Li anode with PVDF-HFP.

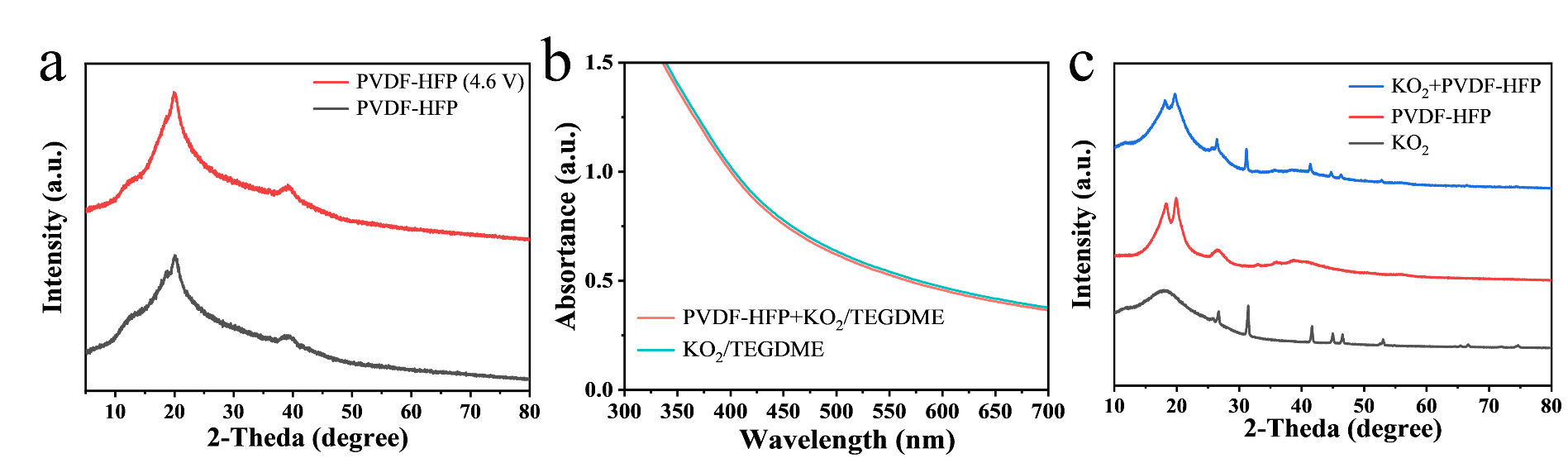

**Figure S20**. (a) XRD patterns of PVDF-HFP before and after the LSV test. (b) UV–vis spectrophotometry of a KO_2_-containing electrolyte before and after the addition of PVDF-HFP. (c) XRD patterns of KO_2_ and the mixture of KO_2_ and PVDF-HFP.


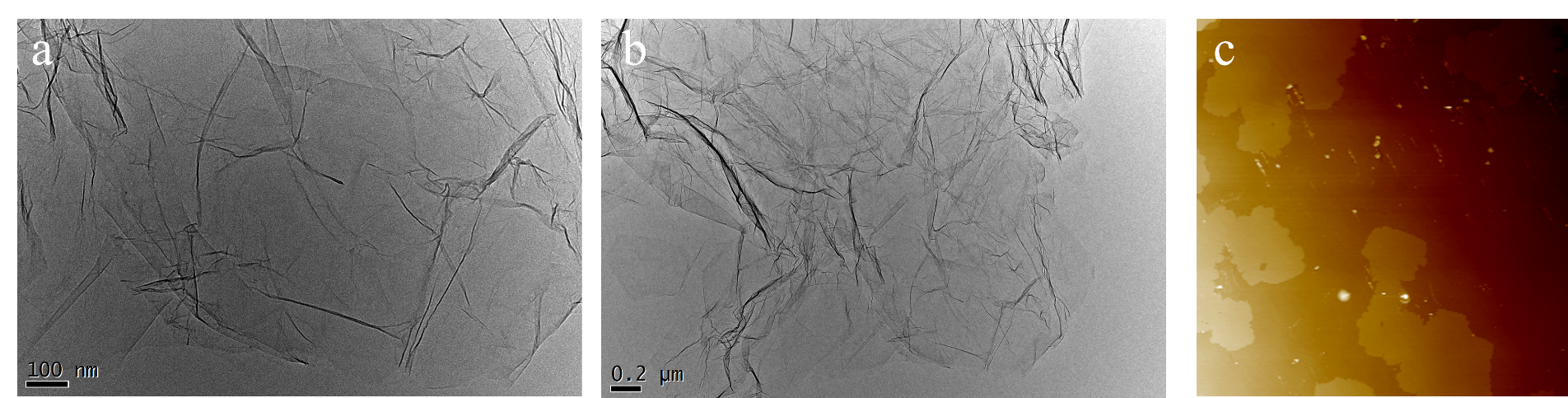


**Figure S21**. (a-b) TEM image and (c) AFM pattern of rGO.


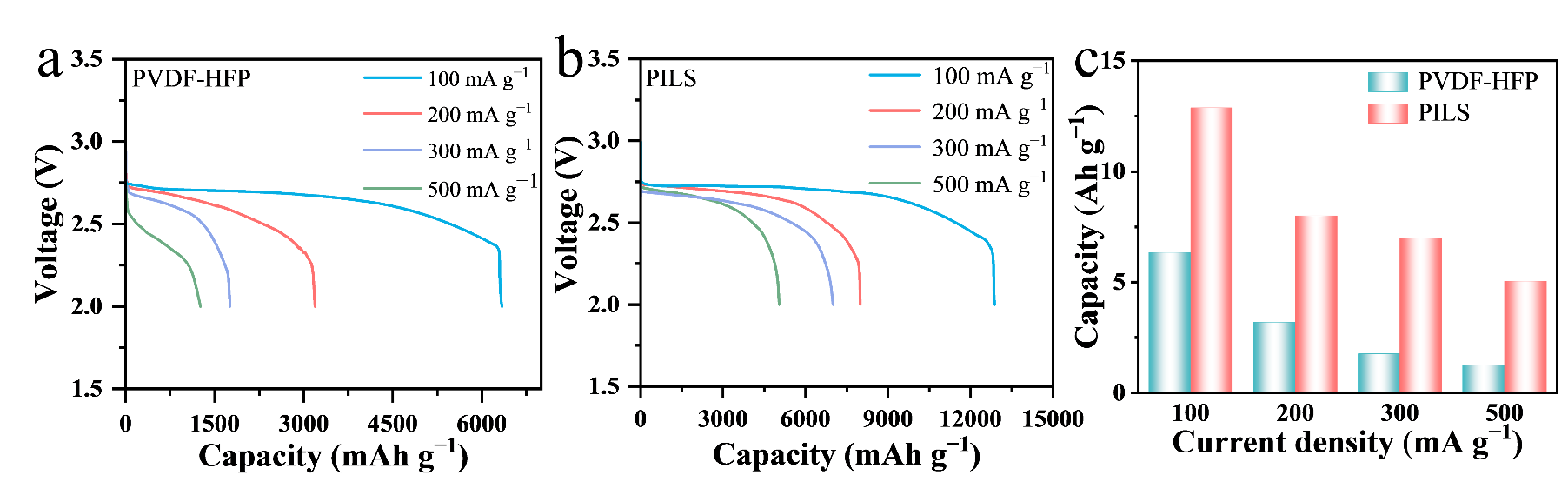


**Figure S22**. The deep discharge profiles of SSLOBs based on (a) PILS and (b) PVDF-HFP at different current density.


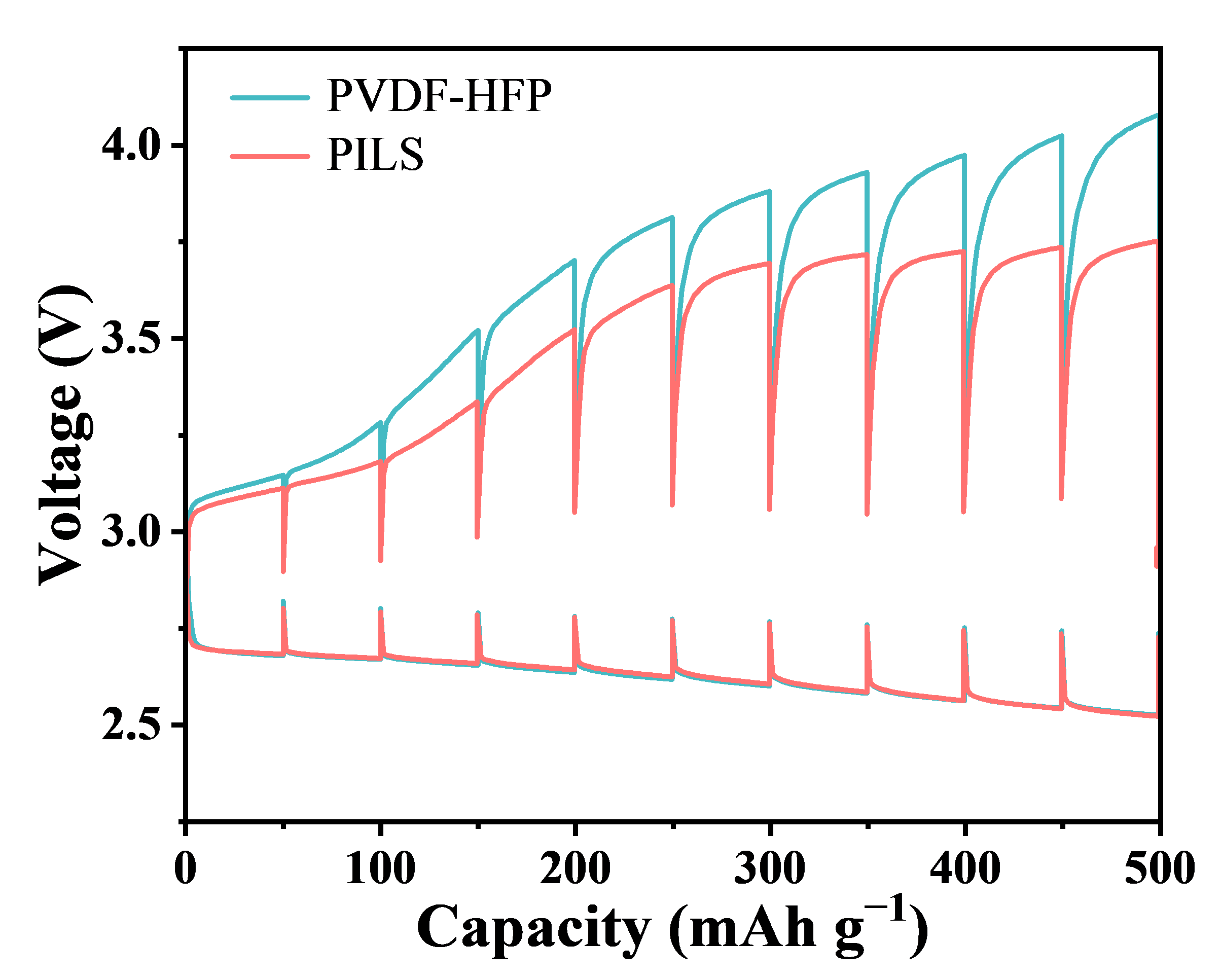


**Figure S23**. Comparison of GITT results to SSLOBs based on PVDF-HFP and PILS.

The galvanostatic intermittent titration technique (GITT) was utilized to substantiate the lower charge voltage of the battery equipped with PILS during the charge phase, as depicted in **Fig. S23**. The SSLOBs underwent discharge at a consistent current density of 200 mA g^−1^ for a duration of 30 min, subsequent to which a 40 min rest period was observed, thereby highlighting the enhanced catalytic performance of the cathode augmented with PILS.


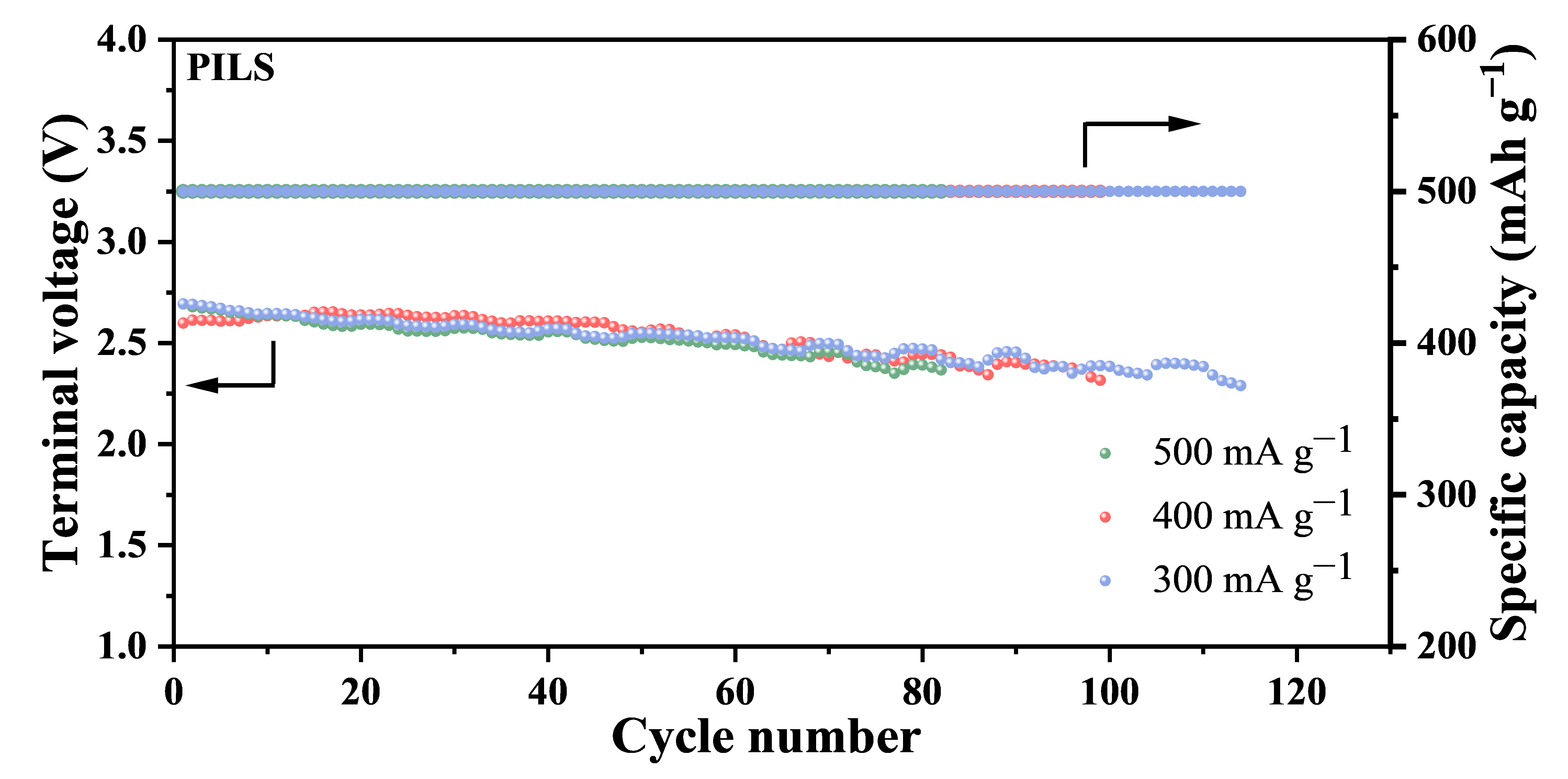


**Figure S24**. Voltage versus cycle number on the discharge terminal of SSLOBs based on PILS at 300 mA g^−1^, 400 mA g^−1^, and 500 mA g^−1^ respectively with a cut-off capacity of 500 mAh g^−1^.

As shown in **Fig. S24**, with a capacity limit of 500 mAh g^−1^, the battery achieved 117 and 82 cycles at current densities of 300 mA g^−1^ and 500 mA g^−1^, respectively. This performance is significantly superior to the PVDF-HFP, which only managed 78 cycles at a current density of 200 mA g^−1^. The ability of the PILS to sustain over 100 cycles at higher current densities underscores a significant improvement in the cycling performance of LOBs constructed with PILS.


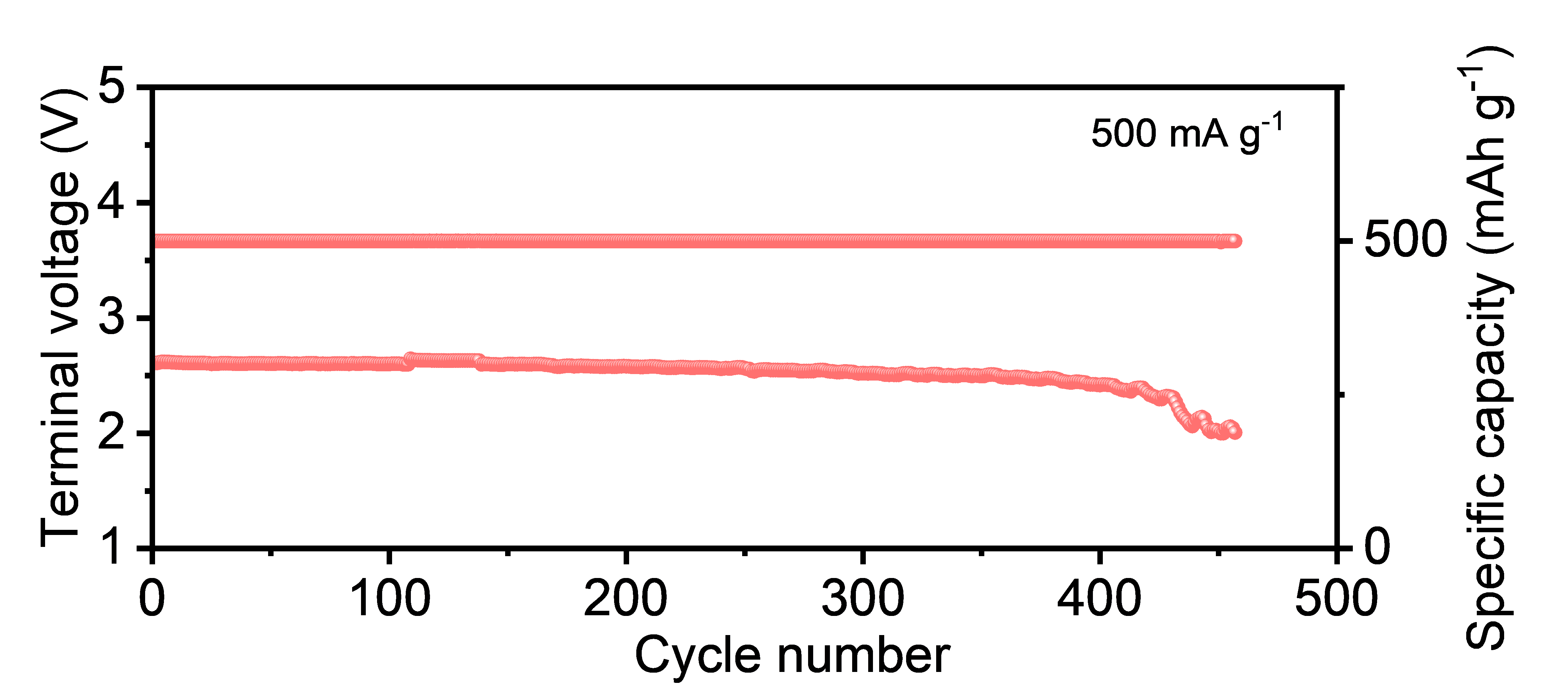


**Figure S25**. Voltage versus cycle number on the discharge terminal of SSLOBs based on PILS at 500 mA g^−1^ with RuO_2_/CNT cathode.


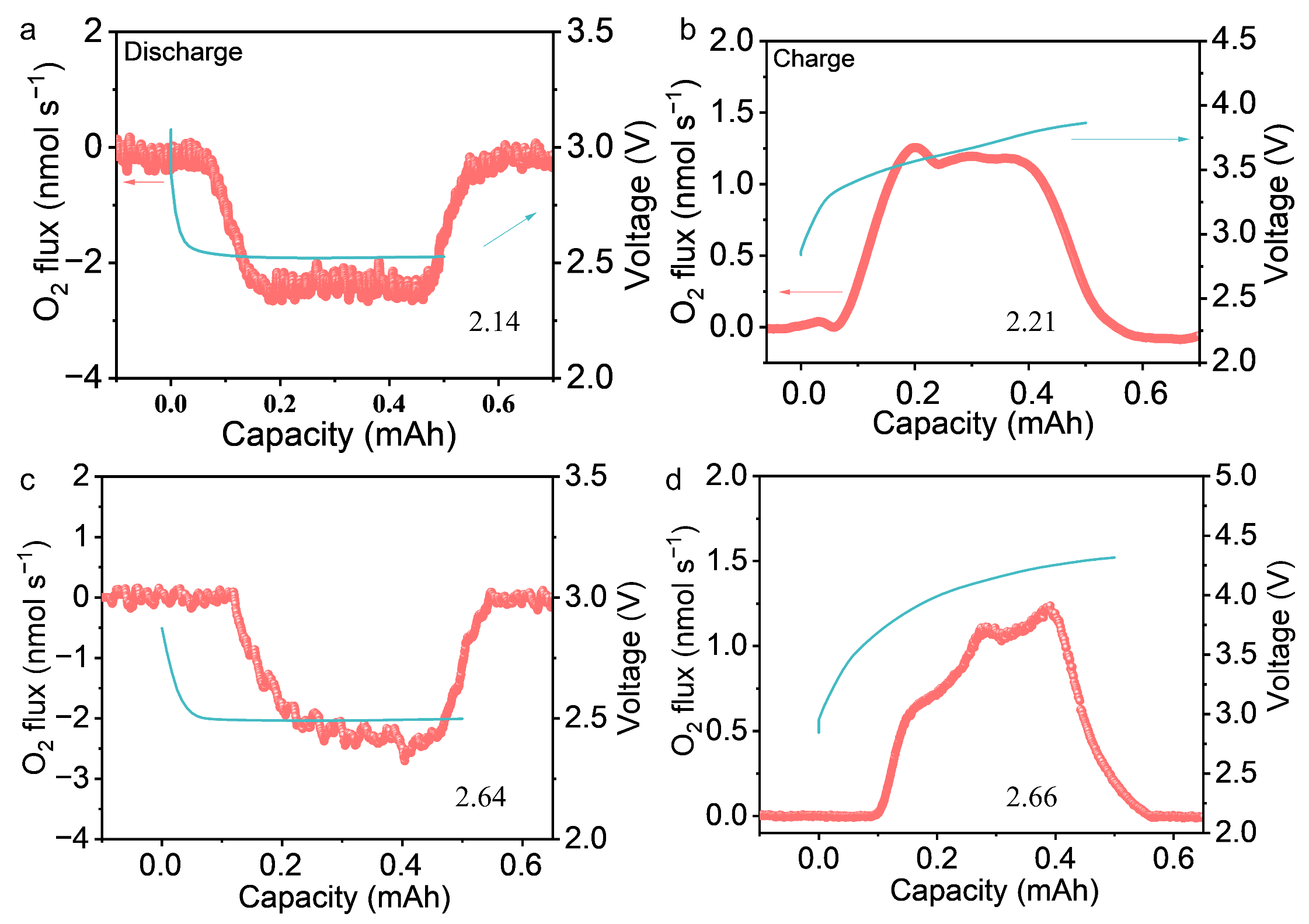


**Figure S26**. In situ DEMS results of the (a) discharge and (b) charge processes of SSLOBs based on PILS and (c) discharge and (d) charge processes of SSLOBs based on PVDF-HFP.

In-situ differential electrochemical mass spectrometry (DEMS) was applied to detect gaseous products during the discharging/charging process. The detected amounts of consumed and released oxygen during discharge/charge showed an e^-^/O_2_ ratio approaching 2.0, indicating the high reversibility of the SSLOBs with PILS. Analysis of parallel experimental conditions revealed that SSLOBs fabricated with PVDF-HFP demonstrated analogous oxygen evolution-reduction capabilities, though with notably attenuated reaction kinetics compared to PILS-modified systems (**Fig. S26**).


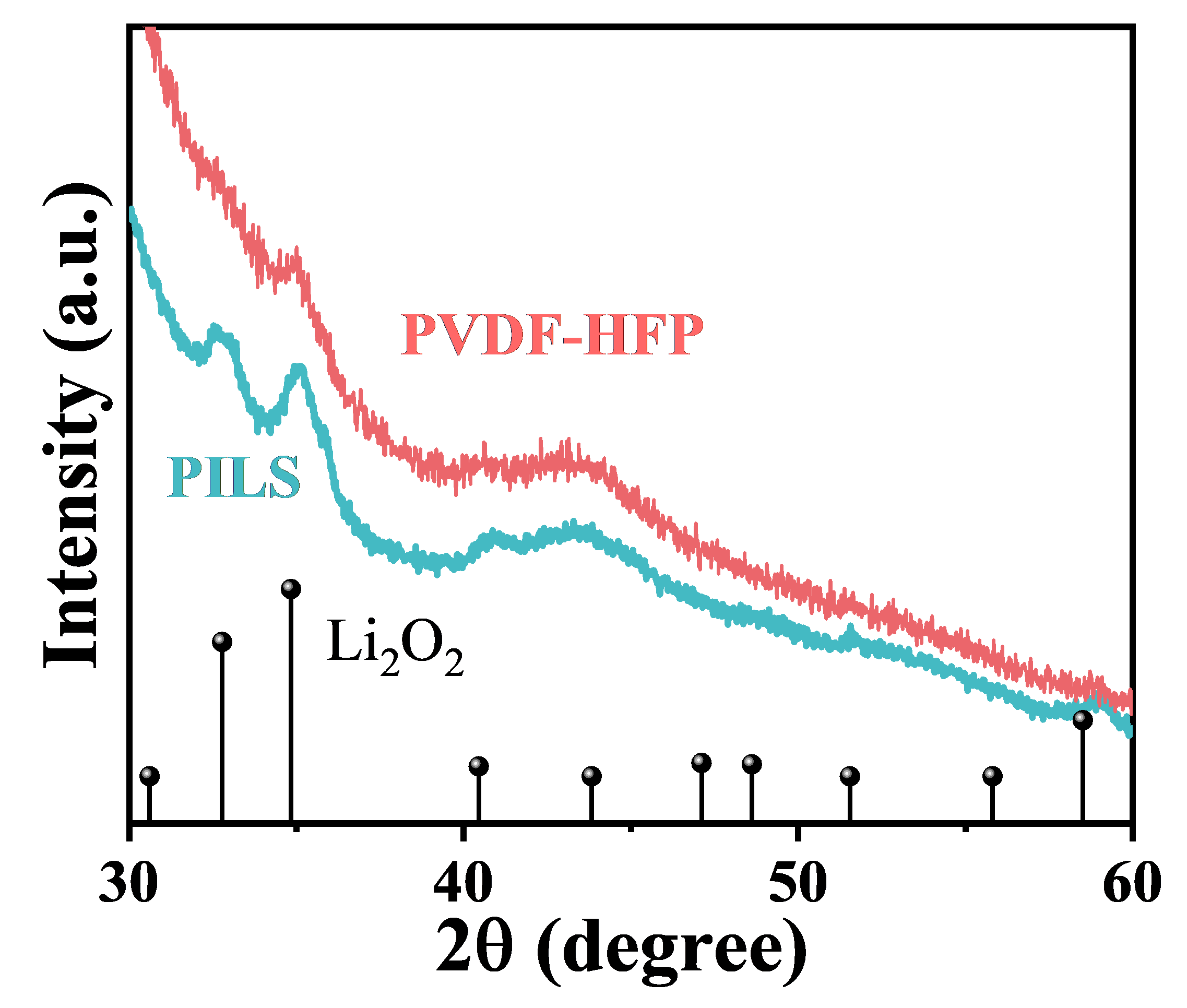


**Figure S27**. XRD of the cathode for deep discharge of SSLOBs based on PILS and PVDF-HFP.

As illustrated in **Fig. S27**, XRD was employed to analyze the composition of the discharge products on the cathode of LOBs utilizing PILS and PVDF-HFP after discharge. The detected discharge products were exclusively Li_2_O_2_, indicating that the solid electrolyte had no impact on the discharge products of the battery. However, compared to the PVDF-HFP, the Li_2_O_2_ peak in the Li-O_2_ battery with PILS is more pronounced, likely due to the higher discharge capacity and increased discharge products associated with PILS.


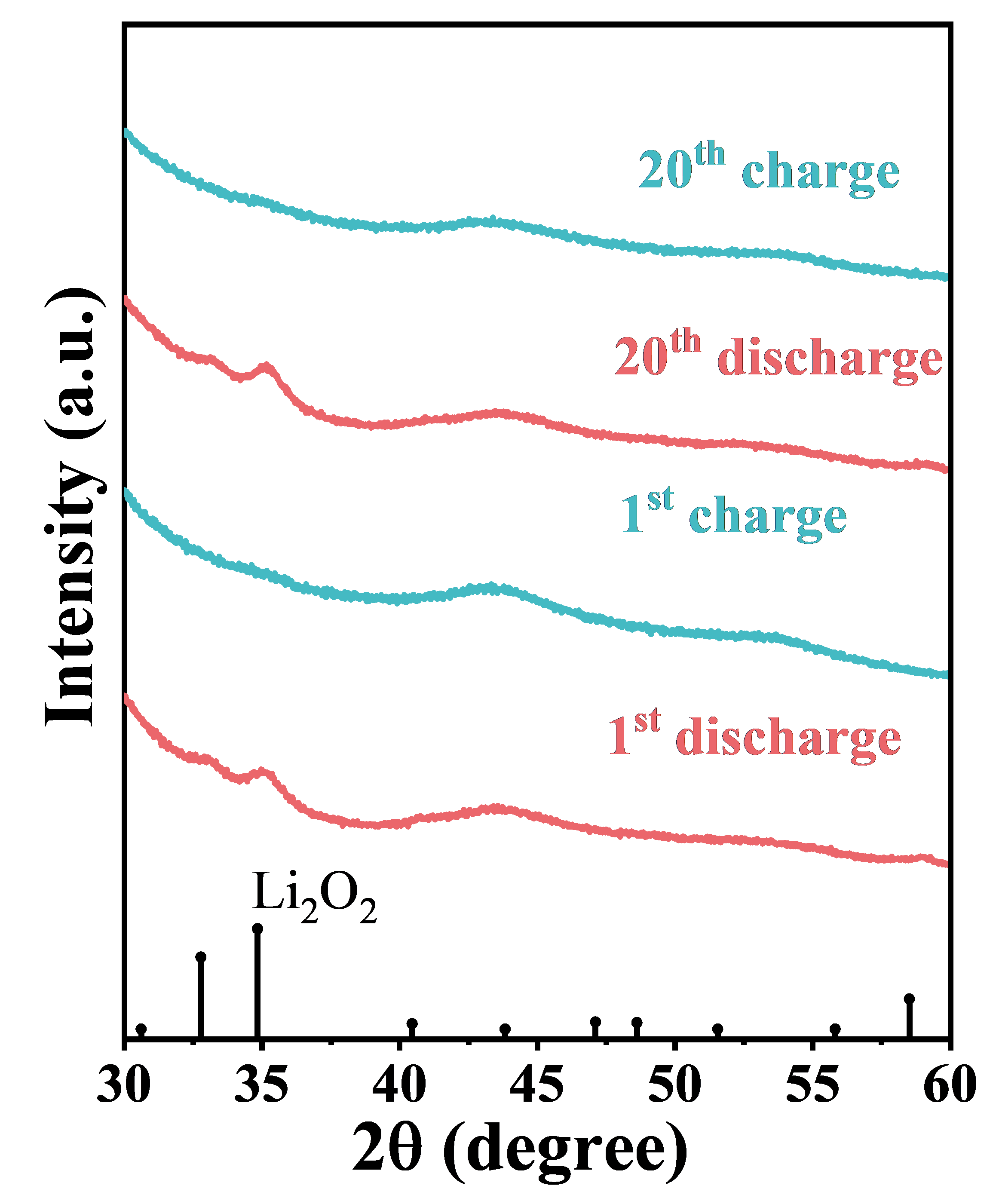


**Figure S28**. XRD of the cathode for 1^st^ discharge/charge and 20^th^ discharge/charge of SSLOBs based on PILS.


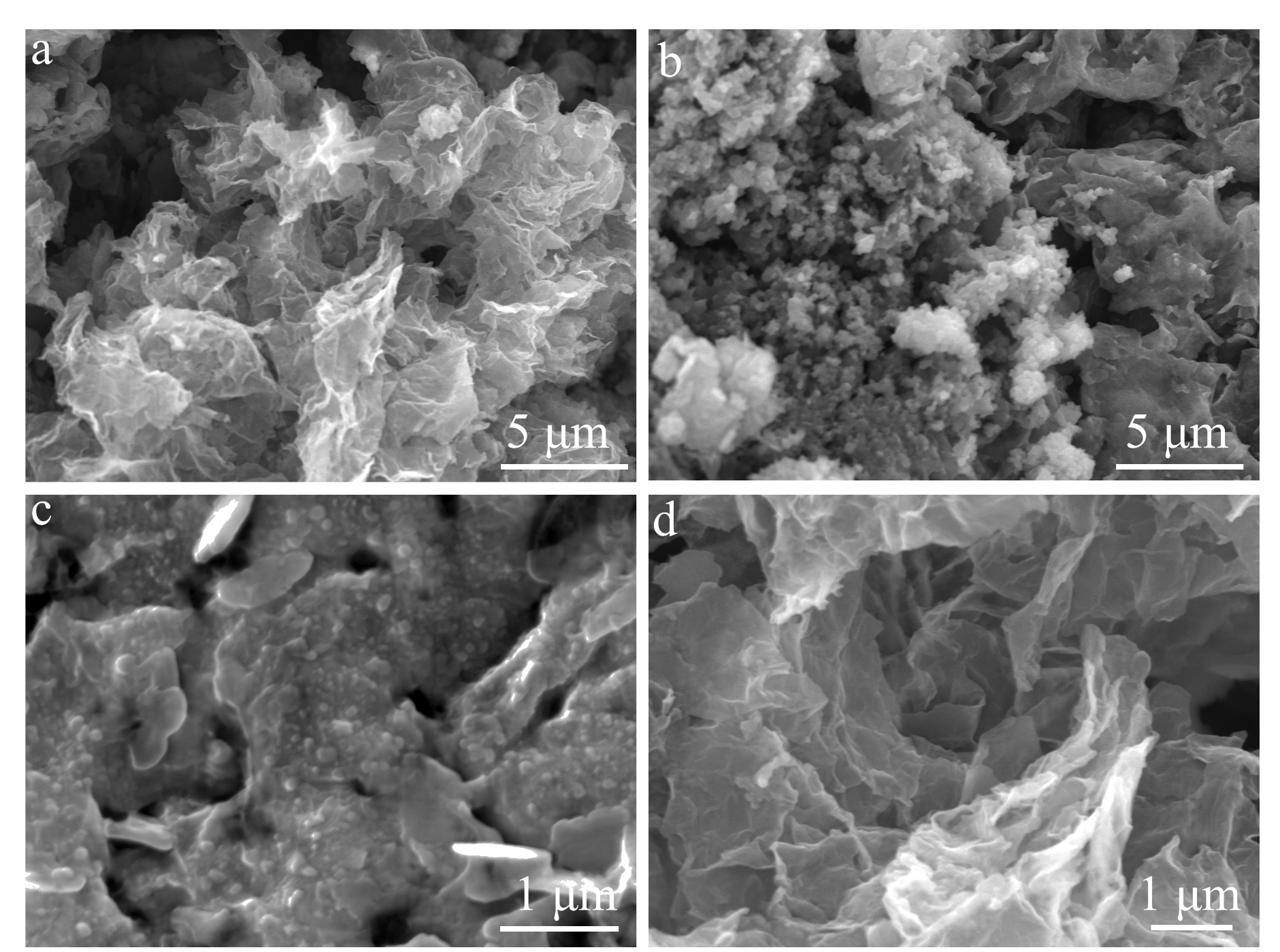


**Figure S29**. SEM image of the solid-state cathode for (a) pristine, (b-c) discharge and (d) charge of SSLOB based on PILS.


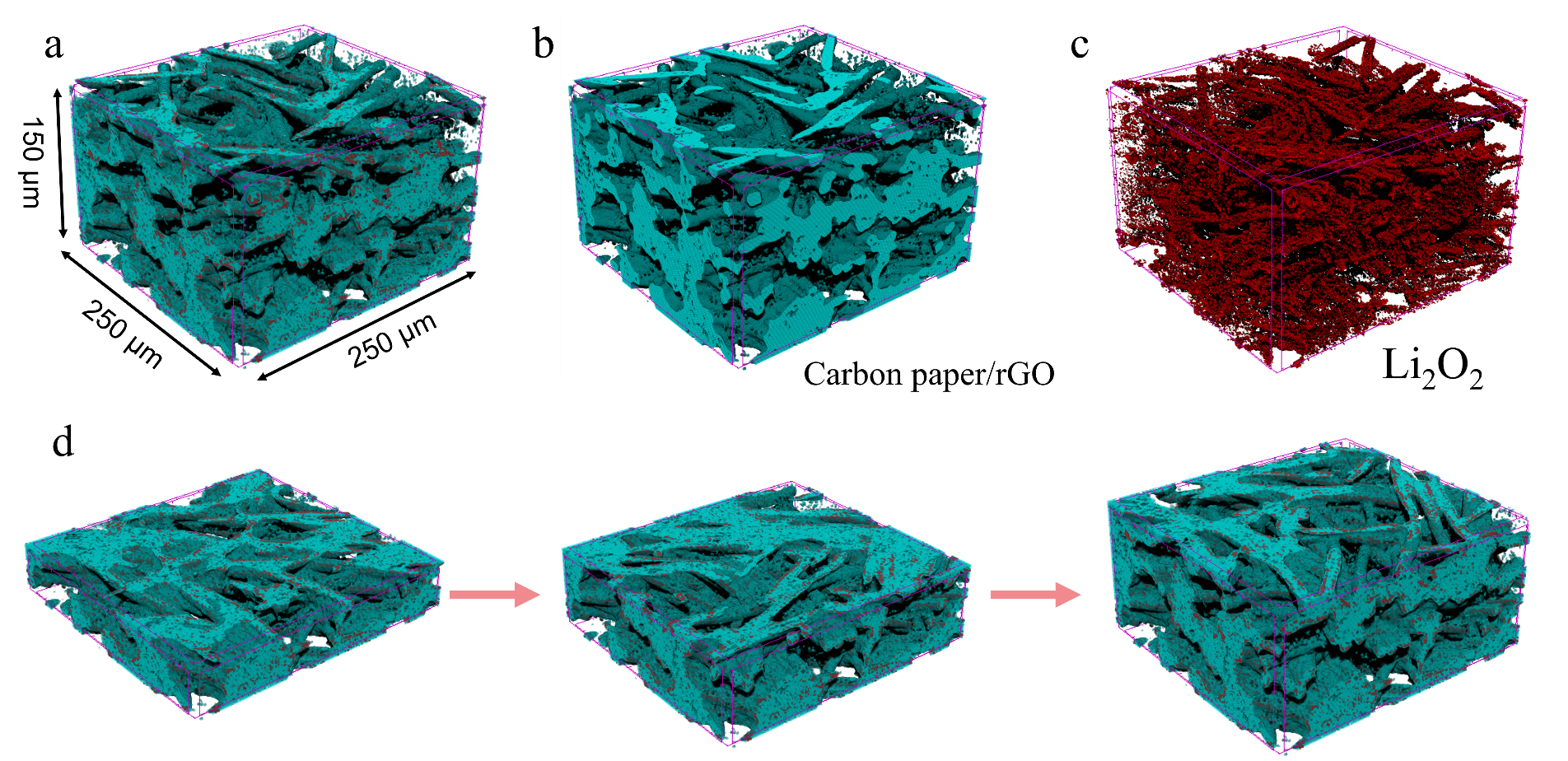


**Figure S30**. In-situ synchrotron CT tomography reconstruction with volume rendering reveals the 3D microstructure of the cathode after discharge. (a) rGO electrodes after discharging. (b) Carbon paper/rGO. (c) discharge products. (d) 3D reconstruction process.


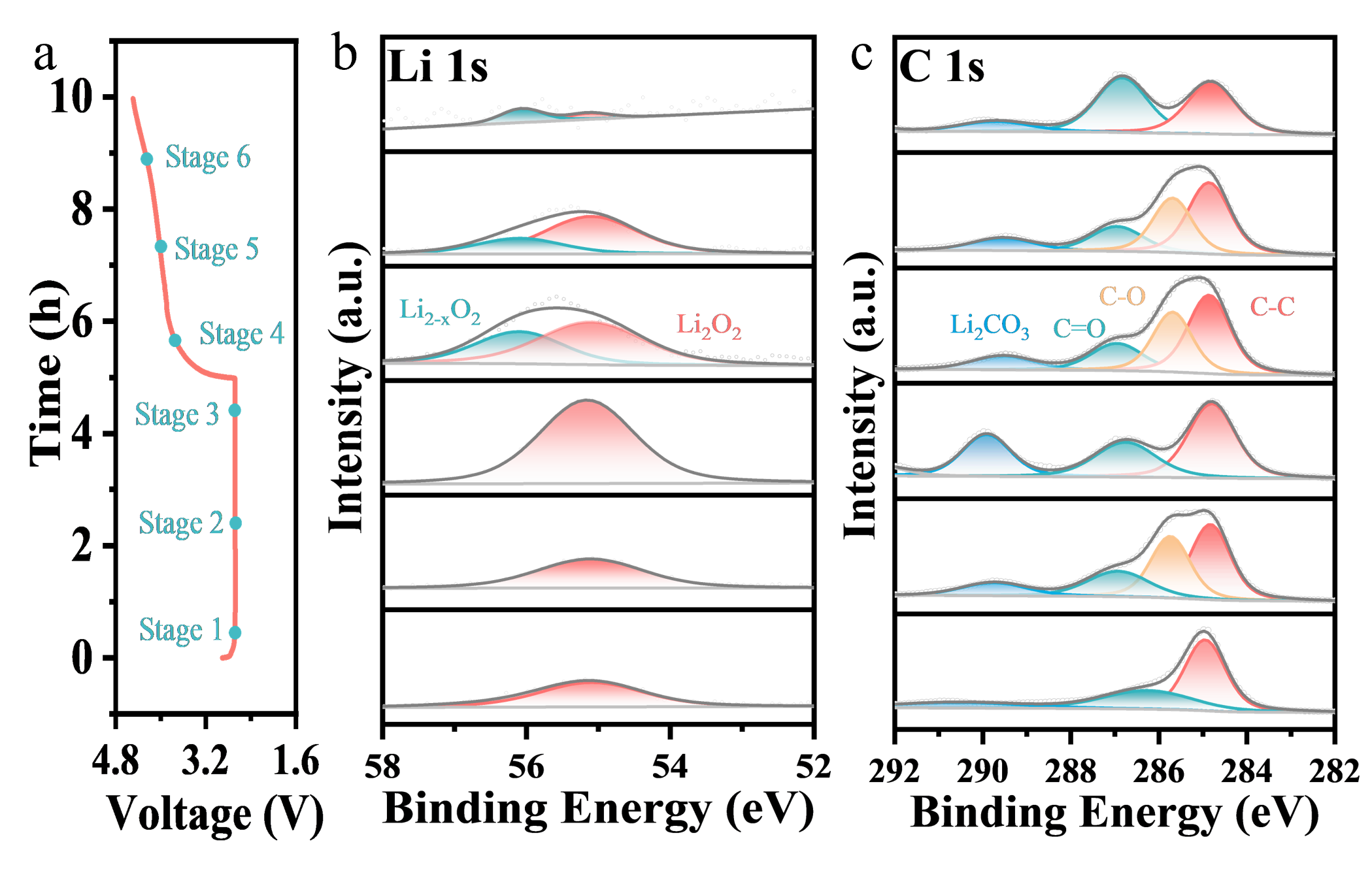


**Figure S31**. (a) Discharge/charge curve of LOB with PILS electrolyte. XPS spectra of (b) Li 1s and (c) C 1s.


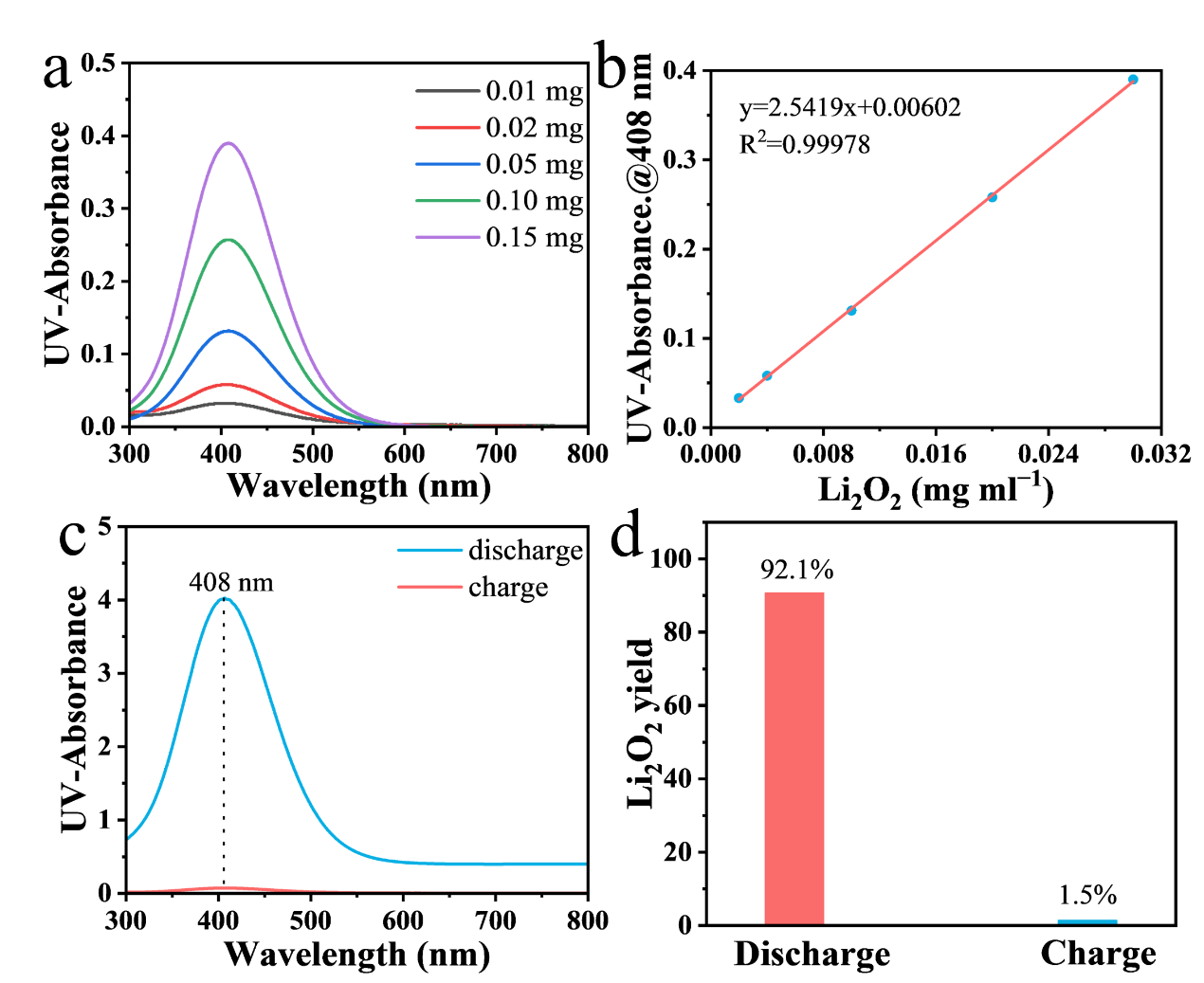


**Figure S32**. (a) UV-vis absorbance responses of various amounts for Li_2_O_2_ powder in TiOSO_4_ solution. (b) Calibration curve based on (a). (c) TiOSO_4_-based UV–vis titration in discharge and charge process, respectively. (d) The corresponding column chart of Li_2_O_2_ yield.

When titrating cathode after discharge, it can be clearly observed that the standard solution changes from colorless to yellow orange, indicating that Li_2_O_2_ reacts with TiOSO_4_ to form [TiO_2_]^2+^SO_4_^2−^, which is consistent with the characteristic peak at 408 nm in the UV spectrum (**Fig. S32**).^[8]^ A high Li_2_O_2_ yield of up to 92.1 % is obtained for SSLOB with PILS, and a residual Li_2_O_2_ yield of 1.5 % is calculated after the charging process, further validating the high reversibility and efficiency of the battery system.


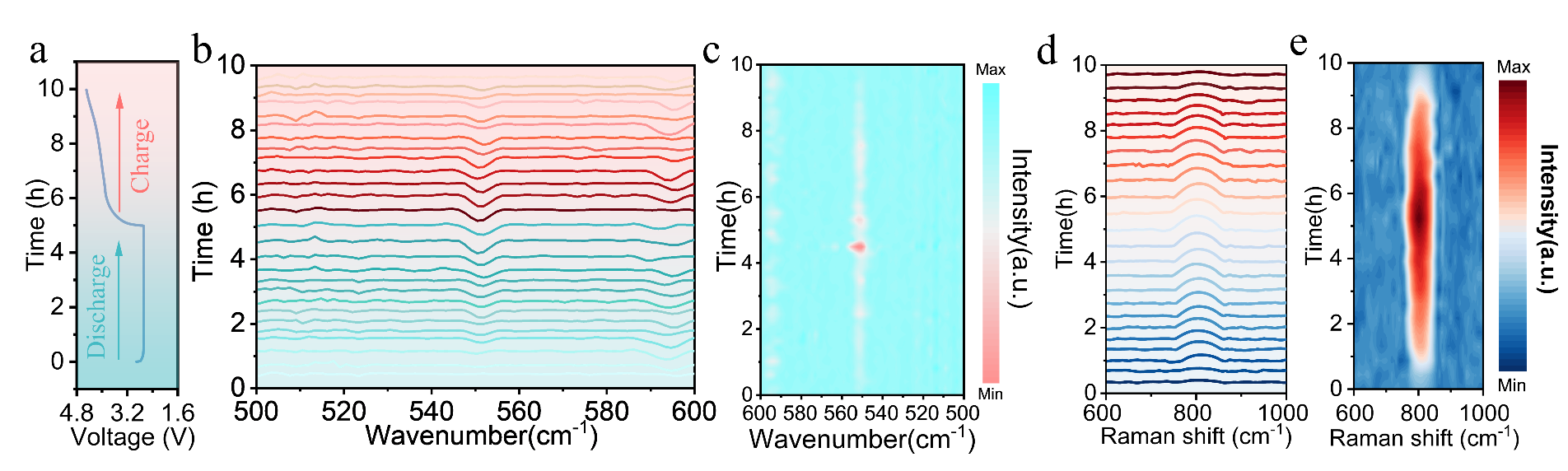


**Figure S33**. (a) Galvanostatic charge/discharge curves of SSLOBs for in situ FTIR and Raman. In situ FTIR of (b-c) and Raman (d-e) spectra, taken on the cathodes of SSLOBs based on PILS at different discharge/charge depths to illustrate the reversible Li_2_O_2_ formation/decomposition.


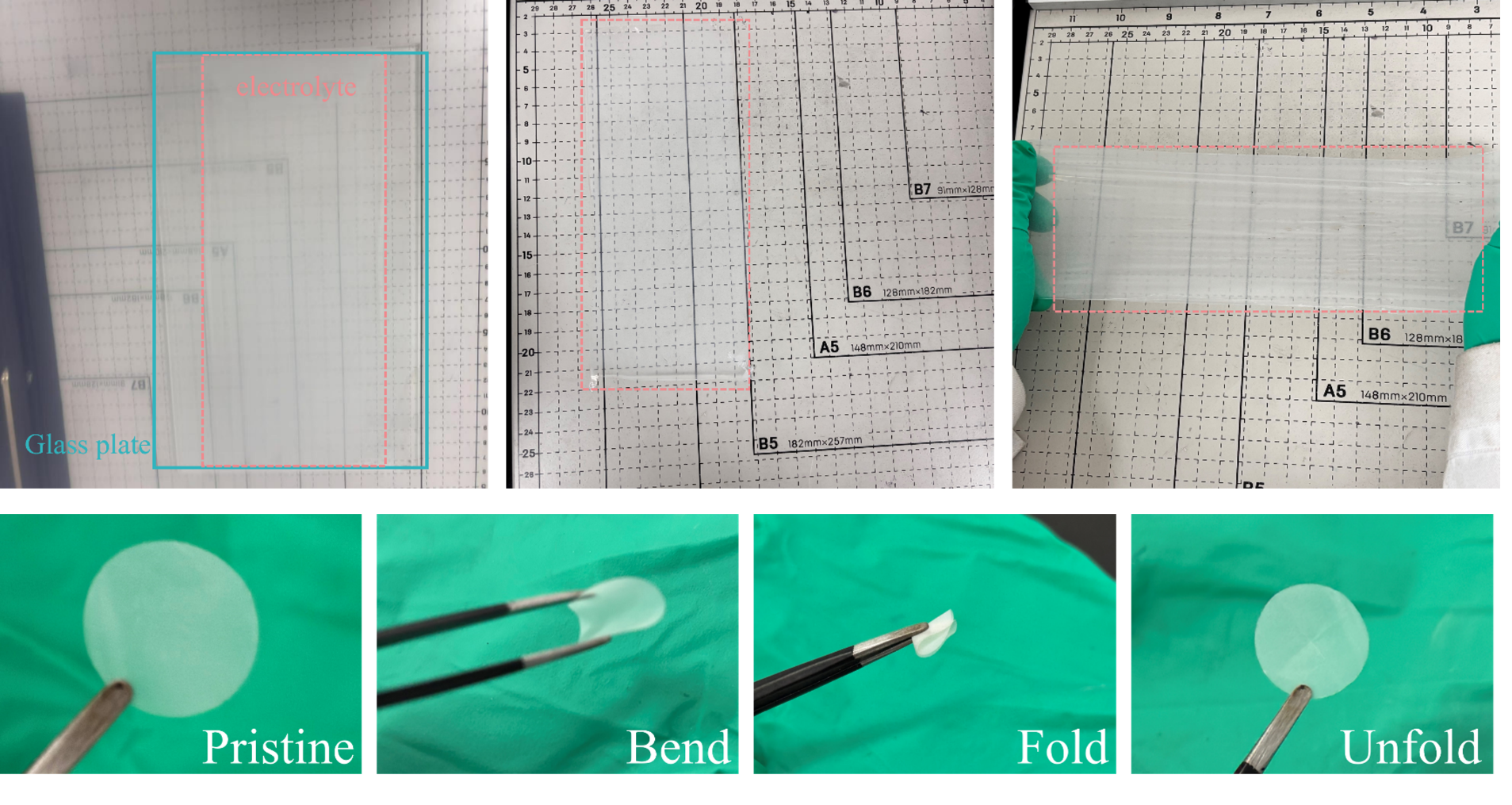


**Figure S34**. Photo images of PILS free-standing membranes being abused via bending, folding, and unfolding.


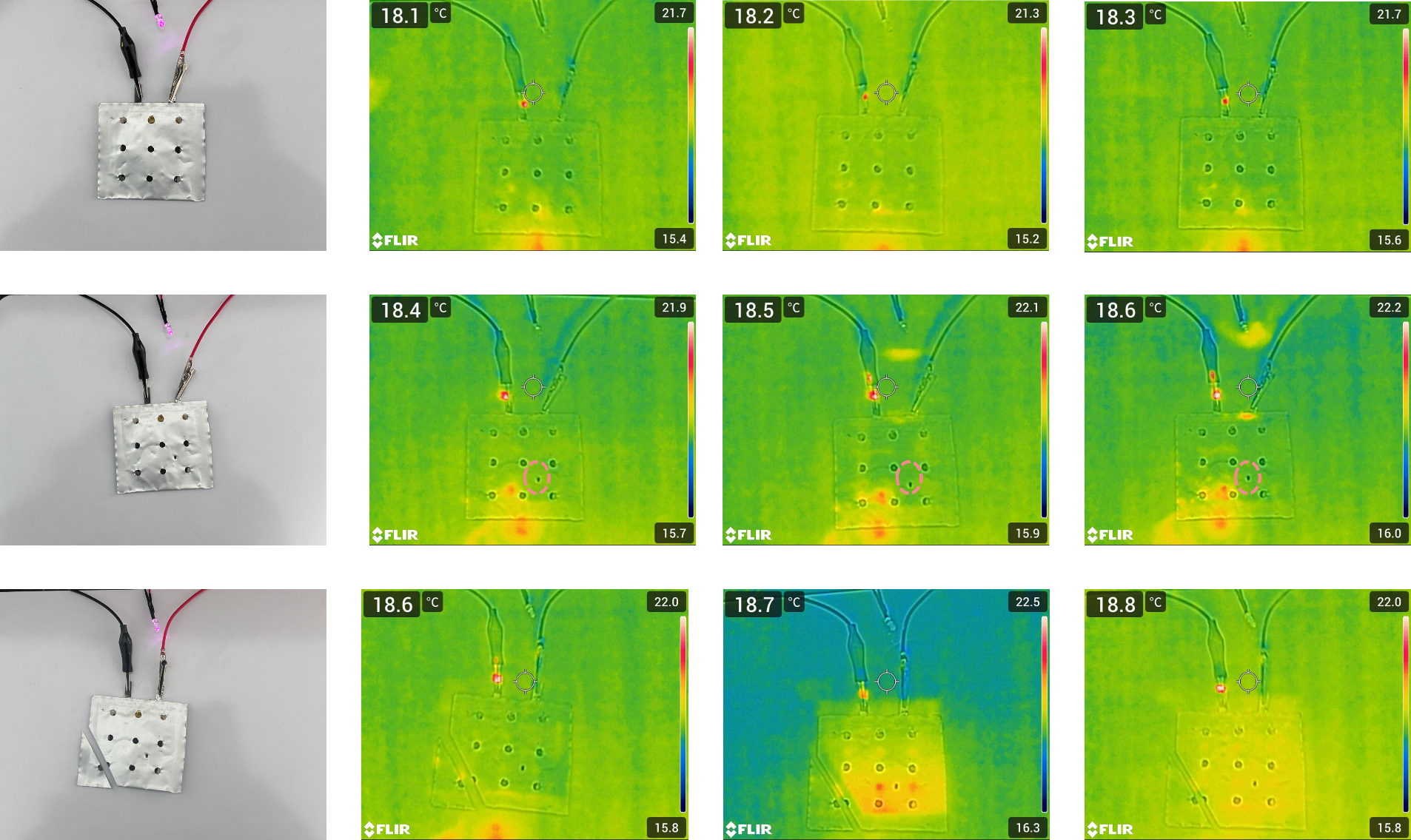


**Figure S35**. The battery temperature observed through an infrared camera.


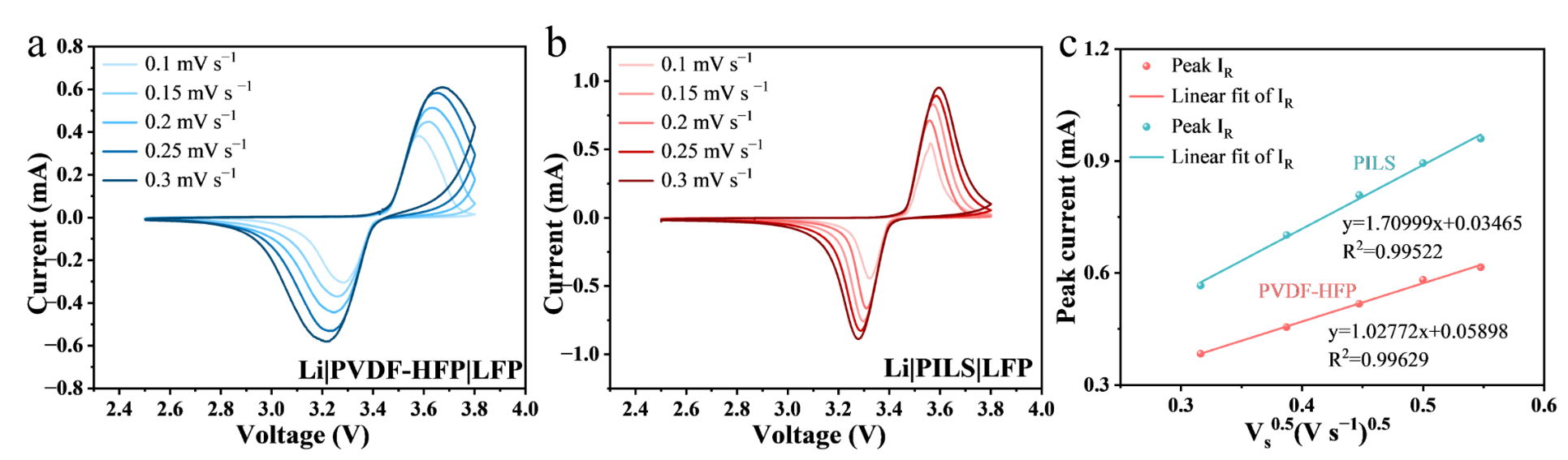


**Figure S36**. CV curves at different scan rates for (a) Li|PVDF-HFP|LiFePO_4_ and (b) Li|PILS|LiFePO_4_ full cell. (c) The linear fitting result of peak current as a function of the square root of the scan rate of PILS and PVDF-HFP.

As depicted in **Fig. S36**, the solid-state Li-metal battery integrated with the PILS displayed a pair of broad voltage peaks at 3.3/3.6 V, with consistent overlap across different cycles, signifying the reversible redox electrochemical reactions characteristic of LiFePO_4_.


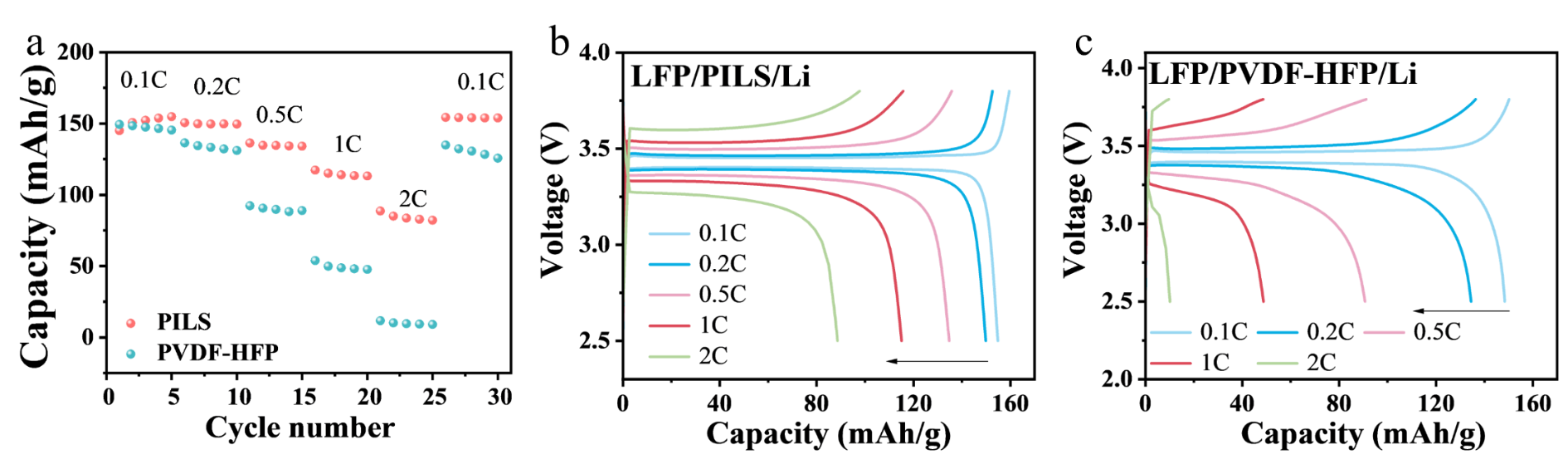


**Figure S37**. (a) The rate performance comparison of LiFePO_4_||Li cell assembled with PILS and PVDF-HFP at room temperature. Discharge/charge curves of LiFePO_4_||Li cell assembled with (b) PILS and (c) PVDF-HFP at different rates.

Furthermore, the rate capability of the Li/PILS/LiFePO_4_ battery was assessed over a voltage range of 2.5–3.8 V. **Fig. S37** illustrates that the battery achieved specific capacities of 155, 140, and 120 mAh g^−1^ at 0.1 C, 0.5 C, and 1 C current rates, respectively. Even when subjected to a high rate of 2 C, a commendable specific capacity of 90 mAh g^−1^ was retained. Upon reverting the current density to 0.1 C, a significant portion of the initial capacity was restored, reaching 155 mAh g^−1^. In contrast, Li-metal batteries employing PVDF-HFP as the electrolyte exhibited a rapid decline in capacity with increasing cycling rates.


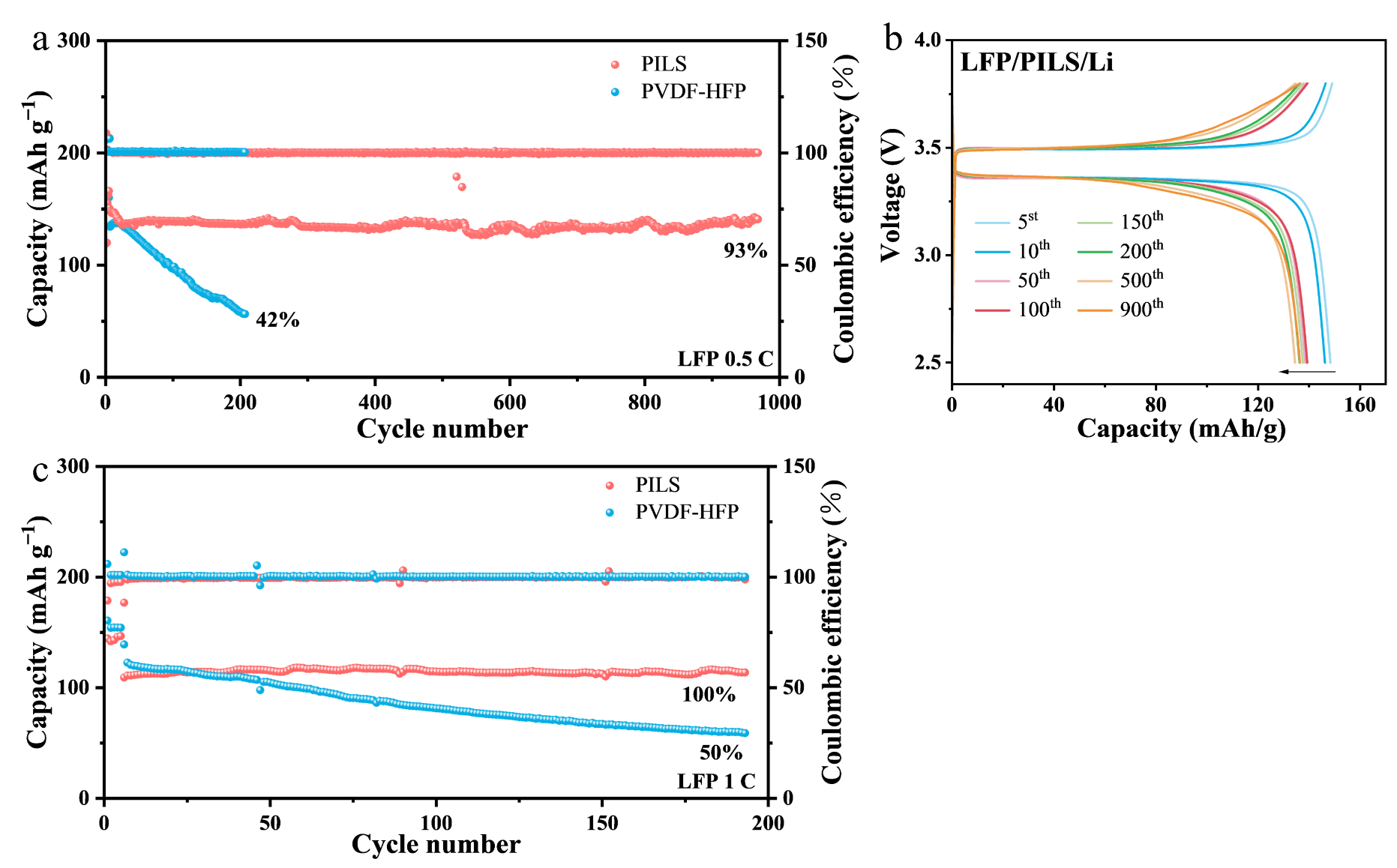


**Figure S38**. Capacity retention performance and coulombic efficiency of LiFePO_4_/Li cell assembled with PILS and PVDF-HFP at (a) 0.5 C and (c) 1 C. (b) Discharge/charge curves of LiFePO_4_/Li cell assembled with PILS at 0.5 C.

The cycling performance further highlighted the superior delivered capacity and outstanding cycling stability of the Li-metal battery with PILS, as evidenced in **Fig. S38**. The battery demonstrated a discharge specific capacity of 150.3 mAh g^–1^ and a capacity retention of 93% after 968 cycles at 0.5 C. At an elevated rate of 1 C, the cells maintained a retained capacity of 108 mAh g^–1^ and a capacity retention of 90% after 195 cycles. In stark contrast, Li-metal batteries with PVDF-HFP exhibited lower discharge capacities (134 and 120 mAh g^–1^) and inferior capacity retention (42% and 50%) after 207 and 195 cycles, respectively. These comparative results underscore the superior performance of the PILS in maintaining excellent electrochemical properties.


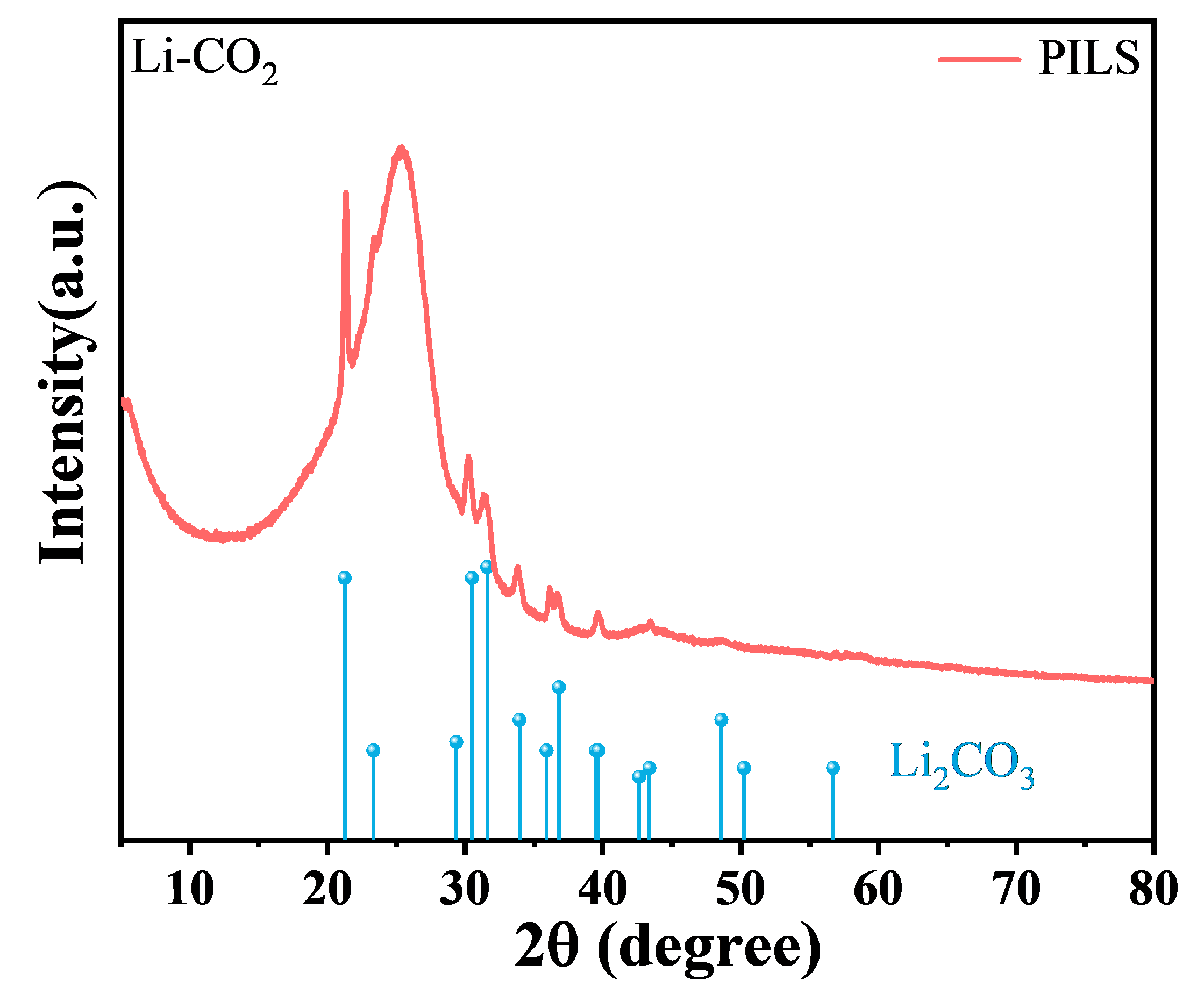


**Figure S39**. XRD patterns of the cathode for deep discharge of solid-state Li-CO_2_ batteries based on PILS

**Table S1**. Summary of sulfide solid-state electrolyte.

| Year | Inorganic components | Organic components | 𝜎 [S cm^−1^] | mg·cm^−2^ | Operating pressure | Interfacial resistance Ω • cm^-2^ | Cycles | Reference |
| --- | --- | --- | --- | --- | --- | --- | --- | --- |
| 2025 | Li_10_GeP_2_S_12_ | PVDF-HFP | 1.05×10^−4^ (25℃) | 0.2 (CNT) | O_2_ | 200-300 | LOB/120 (RT) | This work |
| 2018 | LLZT/Al_2_O_3_ | None | None | 0.5 (Super P) | O_2_ | None | LOB/43 (60℃) | ^9^ |
| 2020 | LLZTO | ILE | 1.71 ×10^−3^ (60℃) | 0.2 (MWCNT) | O_2_ | 87 | LOB/70 (60℃) | ^10^ |
| 2020 | LLZO | PEO | 9.2×10^−5^ | 1 (Ru/KB) | Air | 229 | LOB/50 | ^11^ |
| 2021 | None | SBC | 1.6×10^−3^ | 0.3 (MWCNT) | O_2_ | None | LOB/60 | ^12^ |
| 2023 | CD-COF-Li | None | 2.7×10^−3^ | 0.3-0.5 (CNT) | O_2_ | 1000 | LOB/100 | ^13^ |
| 2021 | Li_10_GeP_2_S_12_ | PTFE | 3.6×10^−4^  (RT) | 6 (LiNi_0.5_Mn_0.3_Co_0.2_O_2_) | Ar | None | 50 | ^14^ |
| 2021 | Li_6_PS_5_Cl | Polydopamine | 2×10^−4^  (RT) | 1.27 (Co_3_S_4_) | Ar | None | 100 | ^15^ |
| 2021 | Li_6_PS_5_Cl (with 0.2 w.t.% SiO_2_ nanoparticles) | PEO | 2.83×10^−4^  (40°C) | 2 (NCM721) | Ar | None | 1000 | ^16^ |
| 2023 | Li_6_PS_5_Cl | PEGDME | 4.5×10^−5^  (25°C) | 5 (NCM and LPSCl) | Ar | None | 100 | ^17^ |

**References**

1. Kresse, G.; Furthmüller, J., Efficient iterative schemes for ab initio total-energy calculations using a plane-wave basis set. 1996, Phys. Rev. B., 54, 11169, https://doi.org/10.1103/physrevb.54.11169

2. Grimme, S.; Ehrlich, S.; Goerigk, L., Effect of the damping function in dispersion corrected density functional theory. 2011, J. Comput. Chem., 32, 1456-1465, https://doi.org/10.1002/jcc.21759

3. Blöchl, P. E., Projector augmented-wave method. 1994, Phys. Rev. B., 50, 17953, https://doi.org/10.1103/PhysRevB.50.17953

4. Grimme, S.; Antony, J.; Ehrlich, S.; Krieg, H., A consistent and accurate ab initio parametrization of density functional dispersion correction (DFT-D) for the 94 elements H-Pu. 2010, J. Chem. Phys., 132, 154104, https://doi.org/10.1063/1.3382344

5. Wang, V.; Xu, N.; Liu, J.-C.; Tang, G.; Geng, W.-T., VASPKIT: A user-friendly interface facilitating high-throughput computing and analysis using VASP code. 2021, Comput. Phys. Commun. 267, 108033, https://doi.org/10.1016/j.cpc.2021.108033

6. Momma, K.; Izumi, F., VESTA 3 for three-dimensional visualization of crystal, volumetric and morphology data. 2011, J. Appl. Crystallogr., 44, 1272-1276, <https://doi.org/10.1107/S0021889811038970>

7. Tan, D. H. S.; Banerjee, A.; Deng, Z.; Wu, E. A.; Nguyen, H.; Doux, J.-M.; Wang, X.; Cheng, J.-h.; Ong, S. P.; Meng, Y. S.; Chen, Z., Enabling thin and flexible solid-state composite electrolytes by the scalable solution process. 2019, ACS Appl. Energy Mater., 2, 6542-6550, <https://doi.org/10.1021/acsaem.9b01111>

8. Kondori, A.; Esmaeilirad, M.; Harzandi, A. M.; Amine, R.; Saray, M. T.; Yu, L.; Liu, T.; Wen, J.; Shan, N.; Wang, H.-H.; Ngo, A. T.; Redfern, P. C.; Johnson, C. S.; Amine, K.; Shahbazian-Yassar, R.; Curtiss, L. A.; Asadi, M., A room temperature rechargeable Li_2_O-based lithium-air battery enabled by a solid electrolyte. 2023, Science, 379, 499-505, [https://doi.org/](https://doi.org/10.1021/acsaem.9b01111)[10.1126/science.abq1347](https://doi.org/10.1126/science.abq1347)

9. Wang, J.; Yin, Y.; Liu, T.; Yang, X.; Chang, Z.; Zhang, X., Hybrid electrolyte with robust garnet-ceramic electrolyte for lithium anode protection in lithium-oxygen batteries. 2018, Nano Res., 11, 3434-3441, https://doi.org/10.1007/s12274-018-1972-5

10. Gao, K.-N.; Wang, H.-R.; He, M.-H.; Li, Y.-Q.; Cui, Z.-H.; Mao, Y.; Zhang, T., Interfacial integration and roll forming of quasi-solid-state Li–O_2_ battery through solidification and gelation of ionic liquid. 2020, J. Power Sources, 463, 228179, <https://doi.org/10.1016/j.jpowsour.2020.228179>

11. Song, S.; Qin, X.; Ruan, Y.; Li, W.; Xu, Y.; Zhang, D.; Thokchom, J., Enhanced performance of solid-state lithium-air batteries with continuous 3D garnet network added composite polymer electrolyte. 2020, J. Power Sources, 461, 228146, https://doi.org/10.1016/j.jpowsour.2020.228146

12. Shi, L.; Wang, G.; Li, J.; Wu, M.; Wen, Z., Sulfonated bacterial cellulose-based functional gel polymer electrolyte for Li–O_2_ batteries with LiI as a redox mediator. 2021, ACS Sustain. Chem. & Eng., 9, 13883-13892, <https://doi.org/10.1021/acssuschemeng.1c05070>

13. Wang, X.-X.; Chi, X.-W.; Li, M.-L.; Guan, D.-H.; Miao, C.-L.; Xu, J.-J., An integrated solid-state lithium-oxygen battery with highly stable anionic covalent organic frameworks electrolyte. 2023, Chem, 9, 394-410, <https://doi.org/10.1016/j.chempr.2022.09.027>

14. Jiang, T.; He, P.; Liang, Y.; Fan, L.-Z., All-dry synthesis of self-supporting thin Li_10_GeP_2_S_12_ membrane and interface engineering for solid state lithium metal batteries. 2021, Chem. Eng. J., 421, 129965, <https://doi.org/10.1016/j.cej.2021.129965>

15. Liu, G.; Shi, J.; Zhu, M.; Weng, W.; Shen, L.; Yang, J.; Yao, X., Ultra-thin free-standing sulfide solid electrolyte film for cell-level high energy density all-solid-state lithium batteries. 2021, Energy Storage Mater., 38, 249-254, <https://doi.org/10.1016/j.ensm.2021.03.017>

16. Luo, S.; Wang, Z.; Fan, A.; Liu, X.; Wang, H.; Ma, W.; Zhu, L.; Zhang, X., A high energy and power all-solid-state lithium battery enabled by modified sulfide electrolyte film. 2021, J. Power Sources, 485, 229325, <https://doi.org/10.1016/j.jpowsour.2020.229325>

17. Huo, H.; Jiang, M.; Mogwitz, B.; Sann, J.; Yusim, Y.; Zuo, T.-T.; Moryson, Y.; Minnmann, P.; Richter, F. H.; Veer Singh, C.; Janek, J., Interface design enabling stable polymer/thiophosphate electrolyte separators for dendrite-free lithium metal batteries. 2023, Angew. Chem. Int. Ed., 62, e202218044, https://doi.org/10.1002/anie.202218044
